# A randomized controlled cross-over trial investigating the acute inflammatory and metabolic response after meals based on red meat, fatty fish, or soy protein: the postprandial inflammation in rheumatoid arthritis (PIRA) trial

**DOI:** 10.1007/s00394-024-03451-6

**Published:** 2024-06-27

**Authors:** Erik Hulander, Linnea Bärebring, Anna Winkvist, Inger Gjertsson, Helen M. Lindqvist

**Affiliations:** 1 Department of Internal Medicine and Clinical Nutrition, Institute of Medicine, Sahlgrenska Academy, University of Gothenburg, Gothenburg, EH, LB, AW, HL Sweden; 2 Department of Rheumatology and Inflammation Research, Institute of Medicine, Sahlgrenska Academy, University of Gothenburg, Gothenburg, IG Sweden

**Keywords:** Rheumatoid arthritis, Inflammation, Serum lipid profile, Postprandial, Red meat, Fatty fish, Soy protein

## Abstract

**Purpose:**

Rheumatoid Arthritis (RA) has a point prevalence of around 20 million people worldwide. Patients with RA often believe that food intake affects disease activity, and that intake of red meat aggravate symptoms. The main objective of the Postprandial Inflammation in Rheumatoid Arthritis (PIRA) trial was to assess whether postprandial inflammation and serum lipid profile are affected differently by a meal including red meat, fatty fish, or a soy protein (vegan) meal.

**Methods:**

Using a randomized controlled crossover design, 25 patients were assigned to eat isocaloric hamburger meals consisting of red meat (60% beef, 40% pork), fatty fish (salmon), or soy protein for breakfast. Blood samples were taken before meals and at intervals up to 5 h postprandial. The analysis included the inflammation marker interleukin 6 (IL-6) and serum lipids.

**Results:**

No significant differences in postprandial IL-6 or triglyceride concentrations were found between meals. However, the area under the curve of very low density lipoprotein (VLDL) particle counts, as well as VLDL-4-bound cholesterol, triglycerides, and phospholipids, was higher after the fatty fish compared to both red meat and soy protein.

**Conclusion:**

Postprandial inflammation assessed by IL-6 did not indicate any acute negative effects of red meat intake compared to fatty fish- or soy protein in patients with RA. The fatty fish meal resulted in a higher number of VLDL-particles and more lipids in the form of small VLDL particles compared to the other protein sources.

**Supplementary Information:**

The online version contains supplementary material available at 10.1007/s00394-024-03451-6.

## Introduction

Rheumatoid Arthritis (RA) is a common autoimmune disease that affects millions of people worldwide [1]. The disease is characterized by chronic pain, swelling, and reduced function in the peripheral joints, leading to a reduced quality of life, an increased risk of comorbidities and a reduced life expectancy. Patients with RA also have a higher risk of developing cardiovascular disease (CVD) compared to the general population. This increased risk is attributed to metabolic disturbances (blood lipids, blood pressure, and insulin sensitivity) and disease-related inflammation [2–4]. Long-term dietary interventions in RA have shown improvements in metabolic disturbances and in reducing disease activity and inflammation [5–8]. We have previously reported reductions in disease activity, inflammation markers, and blood lipids in weight stable patients after a Mediterranean-type diet intervention [9–11], in line with the findings of other research groups [12]. Compared to a typical Western diet, the Mediterranean diet contains less red meat and more vegetables and fish. Interestingly, many patients with RA report that red meat has adverse effects on their disease [13–15]. Furthermore, high intake of red meat is associated with increased risk of CVD in the general population in countries with a high sociodemographic index [16], possibly due to higher intake of saturated fat [17]. Considerably less is known about the postprandial metabolism and particularly in patients with RA. However, it is known that eating a meal results in an increase in inflammation and that the total fat intake has an impact on the magnitude of this increase [18, 19]. Interleukin-6 (IL-6) is one of the biomarkers of inflammation that is responsive during the postprandial phase [20]. Concurrent increases in triglycerides and glucose have been shown to be followed by a higher number of neutrophiles, and consequently increased production of pro-inflammatory cytokines [21, 22]. In fact, postprandial triglyceride concentrations have been suggested to be more predictive of future CVD development than fasting concentrations [23]. Further, the postprandial state has been suggested to be more informative than the fasting state for assessment of inflammatory response following dietary intervention [24]. In summary, patients with RA have chronic inflammation and an increased risk of CVD, factors that may be impacted by dietary intake. As postprandial metabolism provides important insights into inflammatory and metabolic response to food intake, this state may further our understanding of health effects of diet in RA and may provide a basis for future dietary treatment. To the best of our knowledge, there are no previous studies investigating the postprandial blood lipid or inflammatory response to different isocaloric meals in patients with RA.

## Aim

The main objective of the Postprandial Inflammation in Rheumatoid Arthritis (PIRA) trial was to determine if different sources of dietary protein in isocaloric meals have divergent effects on postprandial metabolism and inflammation in patients with RA.

## Methods

The PIRA trial is Registered at Clinicaltrials.gov (NCT04247009) and was approved by the Swedish Ethical Review Authority (Dnr 2019–05242) and was performed in accordance with the ethical standards laid down in the 1964 Declaration of Helsinki and its later amendments. Recruitment and study start was initiated in January 2019. Due to the Covid-19 pandemic, clinical studies at Sahlgrenska University Hospital and University of Gothenburg, Gothenburg, were halted for 18 months from March 2020 to August 2021, including the PIRA trial. Hence, the study was conducted in two periods: January to March 2019 and August to December 2021.

### Participants

Women with diagnosis of RA (ICD-code M05.9 or M05.8) since at least two years, aged 20–70 years living in the Gothenburg area and attending rheumatology specialist care at Sahlgrenska University Hospital were identified through the Swedish Rheumatology Quality Register. Additional inclusion criteria were body mass index (BMI) 18.5–30.0 kg/m^2^. Exclusion criteria were smoking, allergy or intolerance or unwillingness to consume any of the foods served in the study, pregnancy or breastfeeding, diagnosis of cancer, diabetes, inflammatory bowel disease or celiac disease, use of any lipid lowering medication, glucocorticoids or IL-6 inhibiting therapy during the past 4 weeks, hemoglobin levels < 100 g/L or glycated hemoglobin (HbA1c) above reference range.

### Screening

Weight was measured in standardized hospital gown without shoes, after bladder voidance. Height was measured at the closest cm and waist-hip ratio to the closest 0.5 cm. Body composition was assessed by bioimpedance spectroscopy and dual energy x-ray absorptiometry. Blood samples were collected and c-reactive protein (CRP), erythrocyte sediment rate (ESR), hemoglobin, and HbA1c were measured in fresh blood samples at the Sahlgrenska University Hospital central laboratory. Disease Activity Score 28-joint count (DAS28) was estimated by erythrocyte sedimentation rate and assessments by specially trained research nurses at the Department of Clinical Rheumatology Research Center at the Sahlgrenska University Hospital. Medication use was assessed during interviews and cross-referenced against medical records. Participants also completed a lifestyle questionnaire and a 53-item Food Frequency Questionnaire (FFQ) covering the previous 12 months. A dietary quality index (1–12 points), developed by the Swedish Food Agency to assess habitual quality of diet [25], was assessed based upon this FFQ. An index score of 0–4 points was considered poor dietary quality, 5–8 points fair dietary quality, and 9–12 points a high dietary quality. Physical activity was assessed using scales between 1 and 5 on habitual physical activity and intentional physical exercise. Based on this, a physical activity index between 1 and 4 was calculated, similar to that previously validated by Wareham et al. [26].

### Design

**Fig. 1:**
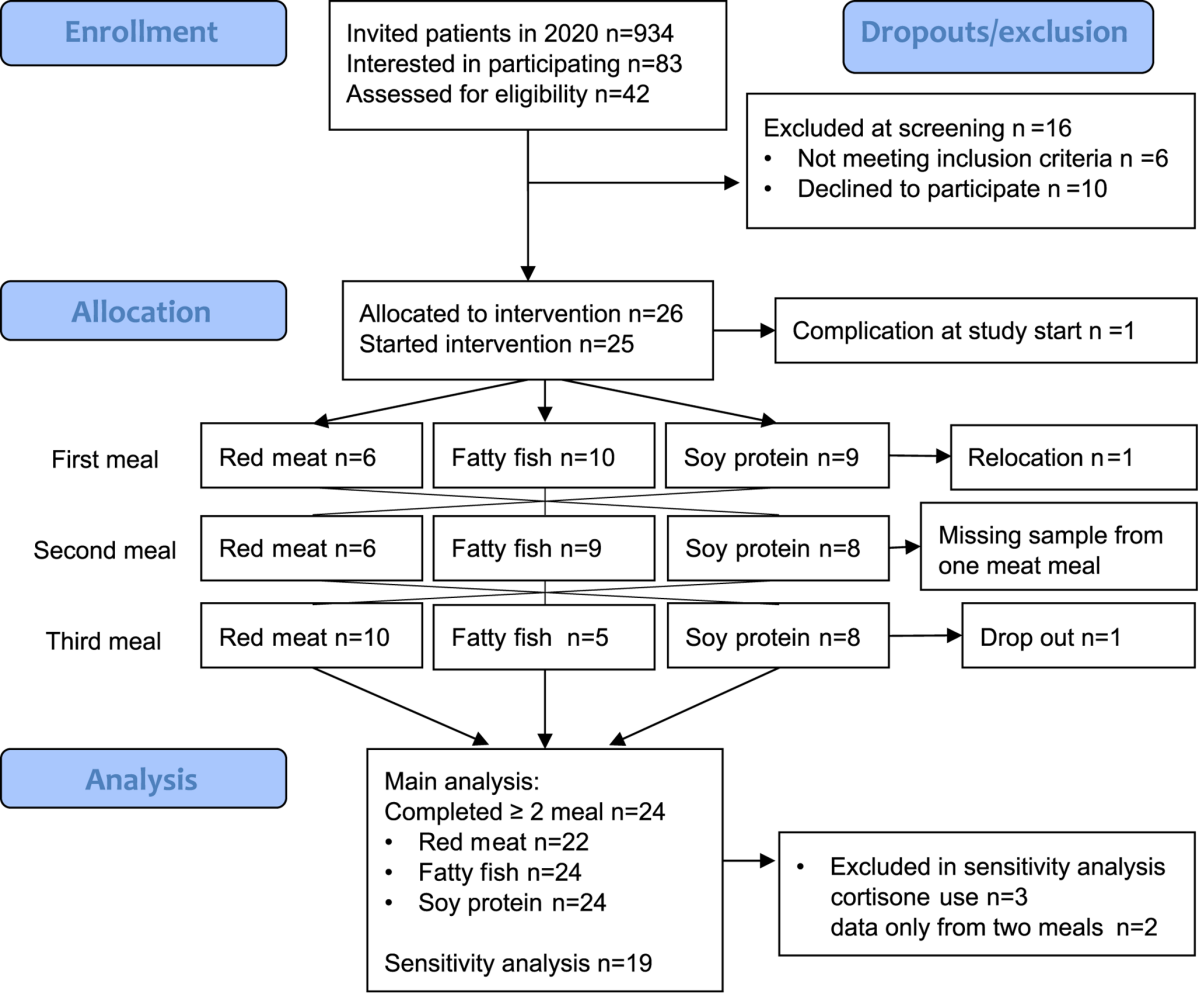
Flow chart of PIRA trial reported according to CONSORT Red meat = meal including minced red meat (60% beef and 40% pork), Fatty fish = meal including minced fatty fish (salmon), Soy protein = meal including a vegan soy protein mince with product name *Anamma Formbar Färs*, produced by Orkla Foods, Sweden

Meals were ingested early in the morning after an overnight fast. The meal sequence was randomized by computer generated randomization (Microsoft Excel using the RAND()-function). The meal sequence allocation was performed after screening, so that at the time of recruitment, the staff did not know which meal order was to be served to patients. There was a minimum one-week washout period between meals. The study design is shown in Fig. 1.

### Outcomes

The primary outcome of the PIRA-trial was differences in postprandial concentration of IL-6 between meals. Secondary outcomes were blood lipids, i.e., triglycerides, very low density lipoprotein (VLDL), high density lipoprotein (HDL), low density lipoprotein (LDL), intermediate density lipoprotein (IDL), VLDL particle concentration and composition of VLDL particles, apolipoprotein (Apo) A1, Apo A2 and Apo B100.

### Intervention meals

The meals consisted of two burgers made of either minced red meat (60% beef, 40% pork), minced fatty fish (salmon), or soy protein (Soy based vegan substitute with product name *Anamma Formbar Färs*, produced by Orkla Foods, Sweden), served on toast with salad, tomato, cucumber and dressing. The burger recipes were carefully composed to contain equal amounts of energy from protein, carbohydrate, and fat (Table 1, Supplemental Table 1). All burgers were cooked in non-stick pans with one teaspoon of canola oil. Fatty fish burgers were cooked until middle temperature reached 52 °C, soy protein burgers to 70 °C, and red meat burgers to 75 °C. Because batches of food commodities might differ slightly, and since nutritional content in different raw products might fare differently through the frying process, samples of cooked burgers were externally analyzed for macronutrients at *Eurofins Food & Feed Testing, Sweden*. Differences in the fat content of the burgers were adjusted for by enriching the fat content of the burger dressing with canola oil (Table 2, Supplemental Table 2). The burgers were produced within a few month before consumption, frozen and stored in -18 °C until served.

**Table 1:** Ingredients in the burgers served during meal challenges, standardized per 1 kg of mince

	Red meat^1^	Fatty fish^2^	Soy protein^3^
Mince (g)	1000	1000	1000
Egg (g)	194	100	0
Breadcrumbs (g)	65	67	23
Salt (ml)	8.5	8.5	1.5 ^4^
Pepper (ml)	3.3	3.3	3.3
Pre-cooked weight per burger (g)	81	73	92

^1^ Red meat (60% beef and 40% pork) produced by Scan, by Swedish meat products

^2^ Fatty fish (salmon) farmed by Salmar Farming AS, Norway

^3^ Soy based vegan substitute with product name *Anamma Formbar Färs*, produced by Orkla Foods, Sweden. Ingredients; water, soy protein (23%), canola oil, salt, spices, natural aromas, caramelized sugar, stabilizing agent (methylcellulose)

^4^ Soy based vegan substitute already included salt

**Table 2:** Nutritional content of the served meals during the meal challenges^1^

	Red meat^2^	Fatty fish^3^	Soy protein^4^
**Burgers**			
Energy (kcal)	336.5	326.7	300.7
Protein^5^ (g)	27.1	26.6	31.3
Carbohydrate^6^ (g)	5.9	5.9	5.6
Fat^5^ (g)	22.9	22.0	17.1
Saturated fat (%)^5^	38	20	7
Monounsaturated fat (%)^5^	49	42	64
Polyunsaturated fat (%)^5^	9	34	27
Eicosapentaenoic acid (%)^5^	0	4	0
Docosahexaenoic acid (%)^5^	0	7	0
**Bread and vegetables**			
Energy (kcal)	217.3	217.3	217.3
Protein^6^ (g)	7.5	7.5	7.5
Carbohydrate^6^ (g)	39.7	39.7	39.7
Fat^6^ (g)	2.9	2.9	2.9
**Hamburger dressing + canola oil**			
Energy (kcal)	137.7	137.7	190.8
Carbohydrate^6^ (g)	1.2	1.2	1.2
Protein^6^ (g)	0.08	0.08	0.08
Fat^6^ (g)	15	15	21
**Total**			
Energy (kcal)	692	682	709
Protein (g)	34.7	34.2	38.8
Carbohydrate (g)	46.8	46.8	46.4
Fat (g)	40.8	39.9	41.0

^1^ Table illustrates the batch produced in 2021, both batches of meals are displayed in Supplemental Table 2

^2^ Burger from red meat (60% beef and 40% pork) produced by Scan, by Swedish meat products

^3^ Burger from fatty fish (Salmon) farmed by Salmar Farming AS, Norway

^4^ Burger from soy based vegan substitute with product name *Anamma Formbar Färs*, produced by Orkla Foods, Sweden

^5^ Data analyzed by Eurofins Food & Feed Testing, Sweden

^6^ Data calculated based on food labels, fiber content not included

The side dishes were identical for all meals and consisted of two slices of wheat toast (84 g, Jättefranska, Pågen AB, wheat flour, water, canola oil, whole wheat sour dough and wheat, malt of barley, yeast, sugar, salt, wheat gluten), 2 leaves of romaine lettuce (~ 10 g), 4 slices of cucumber (~ 10–20 g), 2 slices of fresh tomato (~ 20–30 g), and vegan hamburger dressing (Rydbergs AB, Canola oil, water, cucumber, sugar, tomato puree, onion, cauliflower, vinegar, salt, mustard seeds, carrot, capsicum, wine vinegar, pepper, spices, spice extracts, potato protein, modified starch (corn and potato), stabilizing agent (xanthan gum), acidity regulator (citric acid), preservatives (nitrobenzoate, potassium sorbate), aroma).

### Blood sampling

During meal challenges, a venous catheter was placed and blood samples were first taken in the fasting state. Participants had 20 min to finish their meal, samples were then taken after 30 min, one, two, three, and five hours. Serum was isolated from serum separating tubes (BD Vacutainer, 5 mL, reference no 367,624), centrifuged for 10 min at 2600 g after 30 min at room temperature and 30 min in refrigerator. Serum was separated and immediately stored in -20 °C, and at earliest convenience transferred to -80 °C for storage until analysis in batch.

### Analysis method

**Fig. 2:**
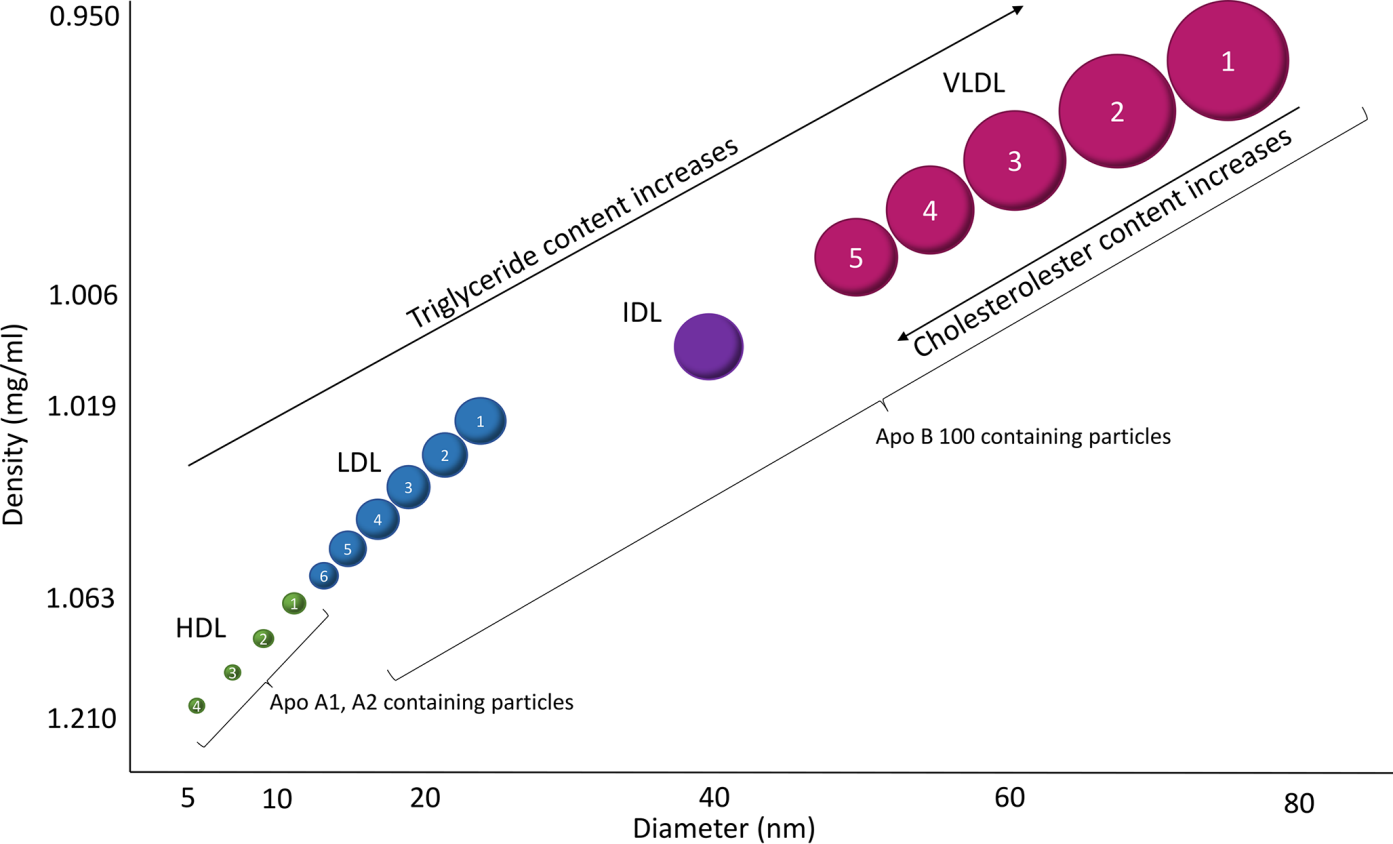
Lipoprotein particles analyzed by NMR. The figure is adapted from Feingold KR [46] Overview of the turnover of triglycerides and associated lipoproteins. Exogenous pathway from the intestine: Fat from food is taken up in apoB-48-containing chylomicrons and to a lesser extent VLDL. The triglyceride content is gradually hydrolyzed by LPL. The resulting remnants are taken up in the liver via lipoprotein receptors. Endogenous pathway from the liver: Fatty acids originate from de novo lipogenesis, FFA from the blood or recycling of remnants. They are secreted in the form of apoB-100-containing VLDL particles. Large VLDL1 particles are broken down by LPL into VLDL remnants in the size class of IDL. These can be further broken down into LDL, taken up by the liver or peripheral tissue, or infiltrate the blood vessel wall directly. The smaller VLDL2 particles are broken down by LPL and HL mainly into LDL. LDL has a long half-life in the blood and a high content of CE, giving them a particularly high atherogenic potential. Immature HDL particles are secreted from the intestine and liver. They exchange CE with both chylomicrons and VLDL particles via CETP and receive structural components from the remnants. The figure is based on ref. (20, 31) VLDL, Very low density lipoprotein; LPL, Lipoprotein lipase; FFA, Free fatty acids; HL, Hepatic lipase; IDL, Intermediate density lipoprotein; LDL, Low density lipoprotein; CE, Cholesteryl esters; CETP, Cholesteryl ester transfer protein

High-sensitive IL-6 was analyzed by Clinical Chemistry at Sahlgrenska University Hospital by.

electrochemiluminescence immunoassay on a Cobas. Serum lipids were quantified by Nuclear Magnetic Resonance (NMR)-analysis and were prepared according to In Vitro Diagnostics Research (IVDr) standard operating procedures (Bruker BioSpin; www.bruker.com/products/mr/nmr/avanceivdr.html) [10]. Serum lipids analyzed are described in Fig. 2. In brief, thawed serum was centrifuged at 3500 x g for 1 min at 4 °C, and 325 µL transferred with a SamplePro L liquid handler (Bruker BioSpin) to a deepwell plate (2 °C) (Porvair, cat. no 53.219030) containing the same amount NMR buffer per well. The plate was shaken for 5 min at 400 rotations per minute (12 °C) in a Thermomixer Comfort (Eppendorf). Finally, 600 µL sample was transferred to 5 mm SampleJet NMR tubes with the SamplePro L. H NMR data was acquired on a Bruker 600 MHz Avance III spectrometer equipped with a room temperature 5 mm BBI probe and a cooled SampleJet sample changer. 1D NOESY (‘noesygppr1d’ pulse sequence), 1D CPMG (‘cpmgpr1d’) and 2D J-resolved (‘jresgpprqf’) spectra were acquired according to the standard IVDr parameter settings at 37 °C. A pre-acquisition temperature stabilization time of 300 s was used. The 1D NOESY data was submitted for B.I.-Lisa lipoprotein profiling and B.I.Quant-PS 2.0.0 automatic quantification of a subset of metabolites through a remote secure Bruker server, generating in total 112 B.I.Lisa and 41 B.I.Quant-PS variables. Experimental parameters are available upon request.

**Fig. 3:**
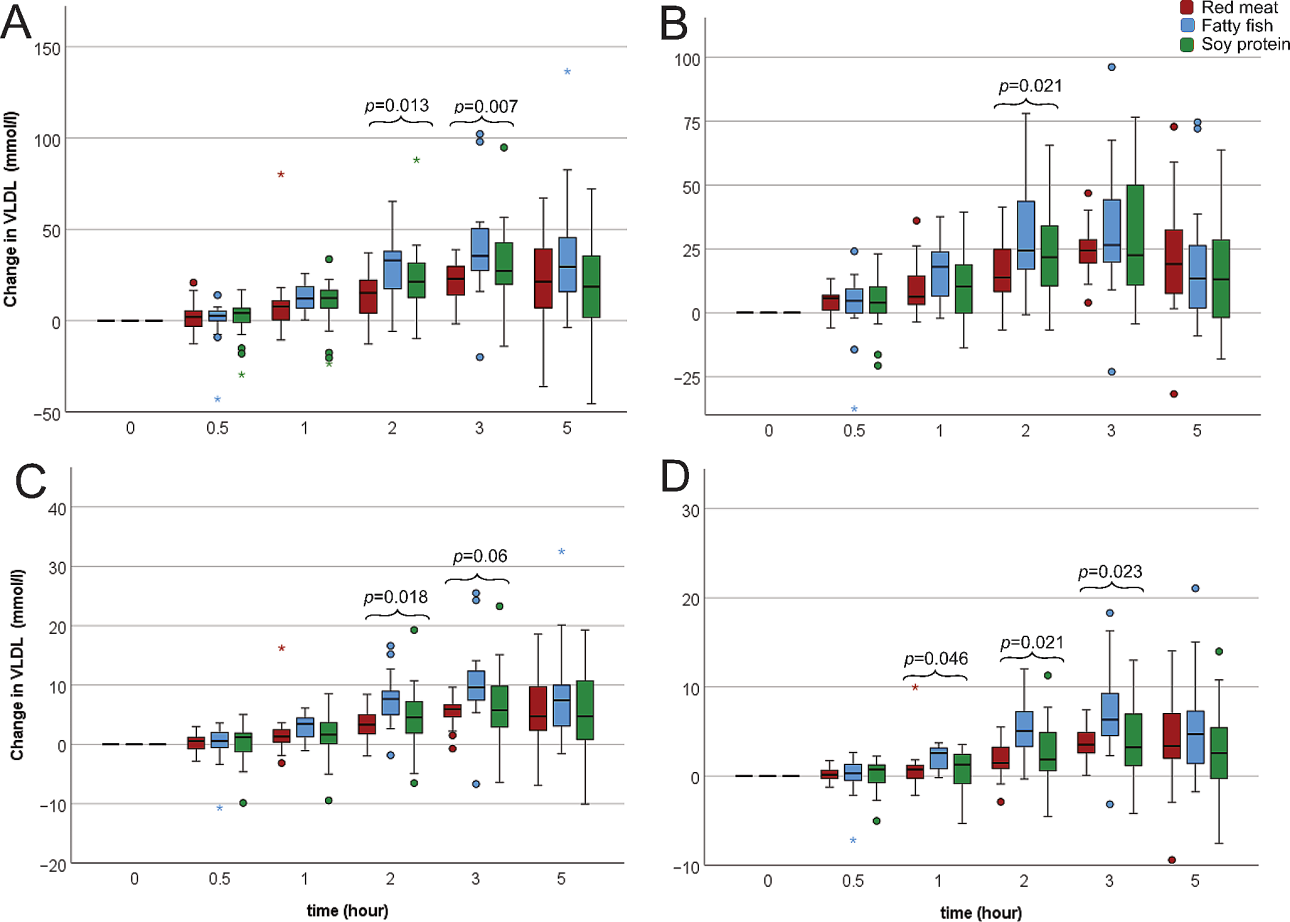
VLDL changes from fasting to postprandial concentrations. (**A**) VLDL particles, (**B**) VLDL triglycerides, (**C**) VLDL cholesterol esters and free cholesterol and (**D**) VLDL phospholipids. Red boxes represent meat meal including minced red meat (60% beef and 40% pork), blue boxes represent fish meal including minced fatty fish (salmon), green boxes represent soy protein meal including a vegan soy protein mince with product name *Anamma Formbar Färs*, produced by Orkla Foods, Sweden

### Statistical analysis

Statistical analyses were performed in IBM SPSS Statistics version 29.0. The main analysis was performed using a mixed model analysis of covariance (ANCOVA) regression with baseline value and treatment as fixed variables, and individual participants as random intercept. IL-6 concentration was included at the 5 h postprandial time point adjusted for the baseline value. For triglycerides and all lipid fractions, incremental area under the curve (AUC_min_), i.e., the area above the lowest value of all time points, adjusted for the baseline value, was analyzed. To decide what lipid data to include, the AUC_min_ was compared between meals by the Friedman test using the exact *P*-value. Those that were significantly different between meals after a Bonferroni correction (*p* < 0.000446 by accounting for 112 outcome variables) were included in the main analysis (Supplemental Table 3). This led to the inclusion of VLDL-4 particle concentrations. To improve clarity, the total VLDL-composition was also included.

All analyses were tested for model assumptions and variables were transformed by its logarithm (base of 10 and the natural logarithm), its square root, or raised to the power of -1, when needed. The models were also tested for confounding by the following variables: age, BMI, DAS28, dietary quality index, and physical activity index. If Beta coefficient of the regression was altered by > 10%, the variable was included as a confounder. For IL-6, the dietary quality index was indicated as a fixed variable. For all lipid data, age, BMI, DAS28, diet quality index, and physical activity index were included as fixed effects. All analyses were also performed without confounders.

As a sensitivity analysis, only participants without any medical changes during the study period and who completed all meal challenges, were included. As a secondary analysis, Friedman test was used to compare concentrations at different time points between meals.

### Power calculation

The power calculation was based on an earlier study indicating that a change in IL-6 of 1.5 pg/mL could be expected [27, 28], with a standard deviation (SD) of 2.0. Thus, with alpha = 0.05 and 80% power, 28 participants would be required. To account for dropouts, the PIRA-study was designed to recruit 40 patients with RA, with the expectation that 30 would complete the trial.

## Results

Of the 934 patients contacted by letter, 83 responded and of those 43 were invited to screening, of those 42 attended screening. In total, 26 participants were allocated, 24 participants completed at least two meal challenges and 22 participants completed all meal challenges (Fig. 1). Most of the participants (56%) were in remission according to DAS28, were highly educated and were mostly middle-aged or older (Table 3).

**Table 3:** Baseline demographical description of participants completing at least 2 meal challenges (*n* = 24)

	Median (p25, p75)
Age (year)	66 (60, 69)
Waist (cm)^1^	85 ± 9
Hip (cm)	102 (97, 108)
BMI (kg/m^2^)^1^	24.8 ± 3.1
Global Health^2^ (0–100 mm)	14 (3, 32)
Pain^2^(0–100 mm)	17 (0, 29)
Fatigue^2^(0–100 mm)	14 (2, 50)
Stiffness^2^ (0–100 mm)	18 (5, 36)
Morning stiffness (min)	22.8 (7.3, 58.1)
Tender joints (no)	1.0 (0.0, 4.5)
Swollen joints (no)	0.0 (0.0, 1.8)
ESR (mm/1 h)	11 (6, 22)
CRP (mg/L)	1.3 (0.6, 3.0)
DAS28 (score)	2.5 (1.9, 3.4)
IL-6 (ng/L)	1.67 (0.95, 3.64)
Triglycerides (mg/dL)	73.0 (64.3, 93.2)
	***n*** **(%)**
Physical activity^3^	
Inactive	3 (13)
Moderately inactive	5 (21)
Moderately active	4 (17)
Active	12 (50)
Educational level	
Junior high school	1 (4)
2-year senior high school	3 (13)
≥ 3-year senior high school	1 (4)
University	19 (79)
Working status	
Not working	10 (42)
< 15 h/week	2 (8)
16–30 h/week	2 (8)
31–40 h/week	6 (25)
> 40 h/week	4 (17)
Ethnic origin	
European	24 (100)
Medication	
Biologic DMARD	10 (42)
Conventional synthetic DMARD	22 (92)

^1^ Mean ± SD

^2^ Self-assessed on a visual analog scale, ranging from 0 to 100 mm

^3^ Based on scales between 1 and 5 on both habitual physical activity and intentional physical exercise, an activity index between 1 and 4 was calculated, resembling what was previously tried and validated by Wareham et al. [26], commonly referred to as the Cambridge Index

Abbreviations: BMI: Body mass index; CRP: C-reactive protein; DXA: dual energy X-ray; ESR: Erythrocyte sedimentation rate; DAS28: 28-joints disease activity score erythrocyte sedimentation rate; IL-6: Interleukin 6; DMARD: Disease modifying anti-rheumatic drugs; VLDL: Very low density lipoprotein

There were no differences in postprandial IL-6 or triglyceride concentrations between the three meals (Table 4, Supplemental Table 3, Supplemental Table 4). However, the number and composition of VLDL particles differed depending on the meal (Fig. 3). Concentrations of postprandial VLDL-particle count were higher after the fatty fish meal compared to both red meat and soy protein. The same pattern was observed for VLDL-bound cholesterol, free cholesterol, and phospholipids, but not for VLDL-bound triglycerides. Regarding the VLDL-4-bound lipids, cholesterol, free cholesterol, phospholipids, and triglycerides were all highest after the fish meal, and lowest after the red meat meal.

Although no significant differences for AUC_min_ for LDL, HDL, or apolipoproteins were found when comparing the three meals, some differences at specific time points or for certain fractions were noted (Supplemental Table 3). Most of the LDL-particles differed between the meals at one or more timepoints. Although the fish meal induced a slightly lower number for most LDL-particles, there was no significant difference in total LDL-particle number. The LDL-particles also differed slightly in triglyceride content at 1, 2, and 3 h with a slightly lower triglyceride content in the larger LDL-particles (size 1, 2, 3) after intake of fish and slightly higher content of cholesterol after intake of red meat in the smaller LDL-particles (size 4, 5, 6). The peak-time differed significantly for several of the measures. In addition, Apo-B100/Apo-A1 ratio was different after 3 and 5 h and had also a difference in peak time (*p* = 0.001) between the meals.

Analysis performed without confounders did not change any results (data not shown). Neither did the sensitivity analysis including only participants who completed all three meal challenges and who did not report any changes in pharmacological treatment (*n* = 19) (data not shown).

All outcomes examined (IL-6, triglycerides, and VLDL-lipid fractions) increased postprandially compared to concentrations in the fasted state (Supplemental Table 5)

**Table 4:** Effect of isocaloric meals with different protein sources on postprandial IL-6 5 h post meal consumption and AUC_min_ concentration of VLDL-lipids, *n* = 24

	Red meat vs. fatty fish		Red meat vs. soy protein		Soy protein vs. fatty fish	
	Median (25p, 75p)	*P*-value	Median (25p, 75p)	*P*-value	Median (25p, 75p)	*P*-value
Il-6^1^ (ng/L)	0.005 (-14.1, 6.31)	0.865	-1.51 (-9.10, 3.43)	0.592	1.00 (-12.9, 7.99)	0.704
Triglycerides^2^ (mg/dL)	-1 640 (-2 480, 2 290)	0.578	-194 (-5 000, 3 730)	0.995	771 (-5 040, 4 360)	0.567
VLDL-4 Cholesterol^2^ (mg/dL)	-168 (-257, -35.1)	< 0.001	-63 (-160, 24.1)	0.035	-35.5 (-238, 63.0)	0.035
VLDL-4 Free Cholesterol^2^ (mg/dL)	-87.3 (-215, -38.9)	< 0.001	-28.0 (-107, 2.29)	0.041	-39.1 (-159, 18.2)	< 0.001
VLDL-4 Phospholipids^2^ (mg/dL)	-171 (-299, -83.7)	< 0.001	-41.7 (-108, 24.2)	0.015	-98.6 (-216, -11.9)	< 0.001
VLDL-4 Triglycerides^2^ (mg/dL)	-247 (-519, -140)	< 0.001	-95.4 (-217, -32.8)	0.031	-159 (-330, -4.58)	0.001
VLDL Particle numbers (nmol/L)	-2 680 (-5 570, -61.0)	0.007	-1 710 (-2 420, 1 030)	0.467	-742 (-3 730, 1180)	0.038
VLDL Cholesterol^2^ (mg/dL)	-605 (-892, -378)	< 0.001	-229 (-681, 425)	0.522	-215 (-1 100, 93.7)	0.001
VLDL Free Cholesterol^2^ (mg/dL)	-173 (-345, -39.0)	0.004	-61.4 (-208, 119)	0.944	-107 (-334, -72.9)	0.004
VLDL Phospholipids^2^ (mg/dL)	-625 (-1 170, -134)	< 0.001	-186 (-579, 269)	0.512	-368 (-1 090, -0.900)	< 0.001
VLDL Triglycerides^2^ (mg/dL)	-1 490 (-4710, 851)	0.195	-1570 (-3420, 2100)	0.769	470 (-4860, 2650)	0.303

^1^Analyzed by a linear mixed model with treatment and baseline value, and diet quality index as fixed effects and subject as random intercepts. Values in table are the difference in median postprandial increase between meals

^2^Analyzed by a linear mixed model with treatment and baseline value, and diet quality index, age, BMI, physical activity index and DAS28 as fixed effects and subject as random intercepts. Values in table are the difference in median AUC_min_ between meals

## Discussion

This trial is the first to examine postprandial inflammatory and metabolic response to isocaloric meals based on different sources of protein, aiming to explore the long standing and mainly anecdotal perception that red meat aggravates symptoms in RA. We found no differences in postprandial IL-6 or triglyceride concentrations between meals. We have identified significant differences in VLDL particle counts and VLDL-composition after the fish meal compared to the meals with red meat or soy. Overall, the results do not support any negative effects of red meat intake in RA.

Differences in postprandial inflammation response after intake of red meat compared to other foods have been reported in few previous studies in different populations. Arya et al. studied the effect of a meal containing 100 g of beef compared to lean kangaroo meat among healthy individuals (*n* = 10) and found that IL-6 increased significantly more after intake of beef [29]. Li et al. investigated the inflammatory response to a hamburger meal (250 g) with or without avocado [30], and noticed an attenuated response in IL-6 after 4 h in the meal with avocado, but found no differences at other time points. However, none of these studies have given any detailed overview of the nutritional composition of the meals, and it seems unlikely that the meals contain the same amount of fat, protein, or carbohydrates. Consequently, effects in these studies probably cannot be ascribed to the individual foods.

A few studies have investigated effects of different protein sources on postprandial triglycerides. In accordance to our findings, red meat did not result in different effects on postprandial triglycerides compared to baked herring [31] or dairy [32]. In addition, the postprandial effect on triglycerides did not differ between salmon and whey protein meals, although the study time of one hour was likely too short to detect any differences [33].

However, we found significant differences in postprandial VLDL-concentrations and compositions between the meals. Our data indicate that VLDL-cholesterol increased most after the fatty fish meal, and the higher cholesterol content the smaller and denser the particles. In addition, VLDL-phospholipids were higher after the fatty fish compared to after the other protein sources. A higher content of VLDL-phospholipids indicates a larger number of particles, since phospholipids only constitute the outer layer of the particles. Indeed, all lipids bound to VLDL-4-particles were higher after the fatty fish compared to the other meals, and VLDL-4 is a relatively small VLDL particle. Thus, our data may indicate that the fatty fish meal resulted in an increased number of smaller VLDL particles compared to the other protein sources. A recent postprandial study on fat quality also found that VLDL-cholesterol was higher after ingestion of a meal with less SFA (similar to the fatty fish and soy protein meal in the current study) than meals with higher SFA content (like our red meat meal) [34]. Burdge et al. found that a high fish oil meal compared to a meal with similar fat content but without n-3 fatty acids, resulted in a higher VLDL concentration and smaller VLDL particle size in the postprandial period, but did not influence triglyceride-, HDL-, or LDL-concentrations [35]. These findings are in line with ours and indicate that n-3 fatty acids from fatty fish cause changes in VLDL number and size. Smaller VLDL particles are associated with lower risk of development of CVD in healthy populations [36, 37] potentially via modification of other lipoproteins such as reduction of highly atherogenic small dense LDL particles [38, 39]. NMR analysis of metabolic biomarkers associated with type 2 diabetes demonstrated strong positive associations between VLDL triglyceride content and VLDL particle size [40]. In addition, NMR analysis of lipoproteins showed that patients with premature CVD had higher concentrations of small dense LDL-cholesterol particles, higher concentrations of large VLDL particles and higher triglyceride content of LDL and HDL particles, and [41]. Thus, our findings are consistent with established beneficial effects of fatty fish intake on CVD risk.

Our results in Supplemental Table 3 show that there are some differences particularly in the number of different sizes and content of LDL-particles, but since the AUC_min_ was not significantly different between the meals for these, we will not speculate about these findings. However, it seems important to be aware of that the peak-time for several particles and their content of lipids may differ depending on the food source.

### Strengths

The PIRA trial, being the first single meal postprandial study on patients with RA, adds considerably to the current knowledge base on dietary impact of health in patients with RA. Earlier studies have shown that total energy content in a meal affect the postprandial response regarding levels of glucose, triglycerides, HDL-cholesterol, and inflammatory markers such as CRP and IL-6 [42, 43]. We have designed an isocaloric intervention with standardized macronutrient composition, which allows for interpretations of the results without confounding by energy content, which has often been the case in previous postprandial trials. Further, the complete hamburger-meal, with a size of around 700 kcal, reflect a realistic meal composition and food matrix with similar matrix (mince) for the compared foods red meat, fatty fish, and soy protein, thereby enabling us to study effects of common foods in free living individuals. Another strength is that we excluded individuals on pharmacological treatment that specifically decrease IL-6 and/or blood lipids. The cross-over setting also reduces the risk that differences in pharmacological treatment would interfere with the results.

Lastly, for the analysis of blood lipids we utilized NMR-spectroscopy according to IVDr standard operating procedures [10]. This is a rapid method that allows the simultaneous quantification of a multitude of lipids and lipoprotein particles without the need to fractionate the sample prior to analysis. As previously demonstrated, this method has been found to be highly reliable and reproducible with low interlaboratory variance [44].

### Limitations

Blood samples were drawn before the meal and at 30 min, 1, 2, 3, and 5 h after. These time points might not capture the full postprandial response. Furthermore, to increase participants’ comfort, we opted to use a venous catheter for blood sampling instead of venipunctures at each time point. This procedure may erroneously cause an increase in IL-6 concentration due to local venous irritation [45]. Still, the randomized controlled crossover design enables comparisons between the meals.

With the pandemic in 2020, and the subsequent resumption of the trial 18 months later, we lost participants. Therefore, the PIRA-trial did not reach the number of participants required based on the power calculation. Consequently, there is an increased risk of type-2 error and the negative results on postprandial inflammation need to be interpreted cautiously. Furthermore, some planned analyses such as adjustment for body composition measured at inclusion, was no longer meaningful at resumption 18 months later. Additionally, we have pooled the data from the two study batches.

To avoid comparing different amount of fat, we added some canola oil (6 g) to the soy protein meal. Although the amounts of oil added was small, it constituted about 15% of the total fat content in the soy protein meal and may have had a minor effect on the outcomes. However, the red meat (pork and beef) and the fatty fish (salmon) meals had similar macronutrient composition and no adjustment had to be done. The generalizability is also limited since we have a sample of mainly middle-aged women with high educational level, with about half of the participants in remission according to the DAS28 score.

## Conclusions

The PIRA trial found no differences in postprandial concentration of IL-6 or triglycerides after intake of red meat, fatty fish, or soy protein. Studies including additional markers of postprandial inflammation are warranted. Intake of fatty fish increased all lipids in the smaller VLDL particles compared to soy protein and red meat. Our data indicate no detrimental postprandial effects of consuming red meat in patients with RA.

## Electronic supplementary material

Below is the link to the electronic supplementary material.

Supplementary Material 1

## Data Availability

Data described in the manuscript will be made available upon reasonable request. Because of Swedish law, data could not be shared publicly.

## Declarations

### Clinicaltrials.gov registration

NCT04247009.

## References

[1] Safiri S, Kolahi AA, Hoy D, Smith E, Bettampadi D, Mansournia MA, Almasi-Hashiani A, Ashrafi-Asgarabad A, Moradi-Lakeh M, Qorbani M, Collins G, Woolf AD, March L, Cross M (2019) Global, regional and national burden of rheumatoid arthritis 1990–2017: a systematic analysis of the Global Burden of Disease study 2017. Ann Rheum Dis 78(11):1463–1471. 10.1136/annrheumdis-2019-215920 31511227 10.1136/annrheumdis-2019-215920

[2] Eriksson JK, Jacobsson L, Bengtsson K, Askling J (2017) Is ankylosing spondylitis a risk factor for cardiovascular disease, and how do these risks compare with those in rheumatoid arthritis? Ann Rheum Dis 76(2):364–370. 10.1136/annrheumdis-2016-209315 27283333 10.1136/annrheumdis-2016-209315

[3] Sundstrom B, Johansson G, Johansson I, Wallberg-Jonsson S (2014) Modifiable cardiovascular risk factors in patients with ankylosing spondylitis. Clin Rheumatol 33(1):111–117. 10.1007/s10067-013-2410-4 24135890 10.1007/s10067-013-2410-4

[4] Han C, Robinson DW Jr., Hackett MV, Paramore LC, Fraeman KH, Bala MV (2006) Cardiovascular disease and risk factors in patients with rheumatoid arthritis, psoriatic arthritis, and ankylosing spondylitis. J Rheumatol 33(11):2167–2172 16981296

[5] Elkan AC, Sjoberg B, Kolsrud B, Ringertz B, Hafstrom I, Frostegard J (2008) Gluten-free vegan diet induces decreased LDL and oxidized LDL levels and raised atheroprotective natural antibodies against phosphorylcholine in patients with rheumatoid arthritis: a randomized study. Arthritis Res Therapy 10(2):R34. 10.1186/ar2388 10.1186/ar2388 PMC2453753 18348715

[6] Holst-Jensen SE, Pfeiffer-Jensen M, Monsrud M, Tarp U, Buus A, Hessov I, Thorling E, Stengaard-Pedersen K (1998) Treatment of rheumatoid arthritis with a peptide diet: a randomized, controlled trial. Scand J Rheumatol 27(5):329–336. 10.1080/03009749850154339 9808394 10.1080/03009749850154339

[7] Kjeldsen-Kragh J, Haugen M, Borchgrevink CF, Laerum E, Eek M, Mowinkel P, Hovi K, Forre O (1991) Controlled trial of fasting and one-year vegetarian diet in rheumatoid arthritis. Lancet (London England) 338(8772):899–902. 10.1016/0140-6736(91)91770-u 1681264 10.1016/0140-6736(91)91770-u

[8] Skoldstam L, Hagfors L, Johansson G (2003) An experimental study of a Mediterranean diet intervention for patients with rheumatoid arthritis. Ann Rheum Dis 62(3):208–214 12594104 10.1136/ard.62.3.208 PMC1754463

[9] Hulander E, Bärebring L, Turesson Wadell A, Gjertsson I, Calder PC, Winkvist A, Lindqvist HM (2021) Proposed anti-inflammatory Diet reduces inflammation in compliant, weight-stable patients with rheumatoid arthritis in a randomized controlled crossover trial. J Nutr. 10.1093/jn/nxab313 34587253 10.1093/jn/nxab313 PMC8643575

[10] Hulander E, Bärebring L, Turesson Wadell A, Gjertsson I, Calder PC, Winkvist A, Lindqvist HM (2021) Diet intervention improves cardiovascular profile in patients with rheumatoid arthritis: results from the randomized controlled cross-over trial ADIRA. Nutr J 20(1):9. 10.1186/s12937-12021-00663-y 33485336 10.1186/s12937-021-00663-y PMC7827982

[11] Vadell AKE, Barebring L, Hulander E, Gjertsson I, Lindqvist HM, Winkvist A (2020) Anti-inflammatory Diet in Rheumatoid Arthritis (ADIRA)-a randomized, controlled crossover trial indicating effects on disease activity. Am J Clin Nutr. 10.1093/ajcn/nqaa019 32055820 10.1093/ajcn/nqaa019 PMC7266686

[12] Forsyth C, Kouvari M, D’Cunha NM, Georgousopoulou EN, Panagiotakos DB, Mellor DD, Kellett J, Naumovski N (2018) The effects of the Mediterranean diet on rheumatoid arthritis prevention and treatment: a systematic review of human prospective studies. Rheumatol Int 38(5):737–747. 10.1007/s00296-017-3912-1 29256100 10.1007/s00296-017-3912-1

[13] Tedeschi SK, Frits M, Cui J, Zhang ZZ, Mahmoud T, Iannaccone C, Lin TC, Yoshida K, Weinblatt ME, Shadick NA, Solomon DH (2017) Diet and rheumatoid arthritis symptoms: Survey results from a Rheumatoid Arthritis Registry. Arthritis Care Res 69(12):1920–1925. 10.1002/acr.23225 10.1002/acr.23225 PMC5563270 28217907

[14] Haugen M, Kjeldsen-Kragh J, Nordvag BY, Forre O (1991) Diet and disease symptoms in rheumatic diseases–results of a questionnaire based survey. Clin Rheumatol 10(4):401–407 1802495 10.1007/BF02206660

[15] Salminen E, Heikkilä S, Poussa T, Lagström H, Saario R, Salminen S (2002) Female patients tend to Alter their Diet following the diagnosis of rheumatoid arthritis and breast Cancer. Prev Med 34(5):529–535. 10.1006/pmed.2002.1015 11969354 10.1006/pmed.2002.1015

[16] Zhang B, Pu L, Zhao T, Wang L, Shu C, Xu S, Sun J, Zhang R, Han L (2023) Global burden of cardiovascular disease from 1990 to 2019 attributable to dietary factors. J Nutr. 10.1016/j.tjnut.2023.03.031 37003507 10.1016/j.tjnut.2023.03.031

[17] Gupta R, Wood DA (2019) Primary prevention of ischaemic heart disease: populations, individuals, and health professionals. Lancet 394(10199):685–696. 10.1016/s0140-6736(19)31893-8 31448740 10.1016/S0140-6736(19)31893-8

[18] Rocha DM, Bressan J, Hermsdorff HH (2017) The role of dietary fatty acid intake in inflammatory gene expression: a critical review. Sao Paulo Med J = Revista paulista de Med 135(2):157–168. 10.1590/1516-3180.2016.008607072016 10.1590/1516-3180.2016.008607072016 PMC9977342 28076613

[19] Monfort-Pires M, Crisma AR, Bordin S, Ferreira SRG (2018) Greater expression of postprandial inflammatory genes in humans after intervention with saturated when compared to unsaturated fatty acids. Eur J Nutr 57(8):2887–2895. 10.1007/s00394-017-1559-z 29098425 10.1007/s00394-017-1559-z

[20] Mazidi M, Valdes AM, Ordovas JM, Hall WL, Pujol JC, Wolf J, Hadjigeorgiou G, Segata N, Sattar N, Koivula R, Spector TD, Franks PW, Berry SE (2021) Meal-induced inflammation: postprandial insights from the personalised REsponses to DIetary composition trial (PREDICT) study in 1000 participants. Am J Clin Nutr 114(3):1028–1038. 10.1093/ajcn/nqab132 34100082 10.1093/ajcn/nqab132 PMC8408875

[21] van Oostrom AJ, Sijmonsma TP, Verseyden C, Jansen EH, de Koning EJ, Rabelink TJ, Castro Cabezas M (2003) Postprandial recruitment of neutrophils may contribute to endothelial dysfunction. J Lipid Res 44(3):576–583. 10.1194/jlr.M200419-JLR200 12562833 10.1194/jlr.M200419-JLR200

[22] van Oostrom AJ, Sijmonsma TP, Verseyden C, Jansen EH, de Koning EJ, Rabelink TJ, Castro Cabezas M (2003) Postprandial recruitment of neutrophils may contribute to endothelial dysfunction. J Lipid Res 44 (3):576–583. doi: 510.1194/jlr.M200419-JLR200200. Epub 202002 Dec 200416 10.1194/jlr.M200419-JLR200 12562833

[23] Bansal S, Buring JE, Rifai N, Mora S, Sacks FM, Ridker PM (2007) Fasting compared with nonfasting triglycerides and risk of cardiovascular events in women. JAMA 298(3):309–316. 10.1001/jama.298.3.309 17635891 10.1001/jama.298.3.309

[24] Telle-Hansen VH, Christensen JJ, Ulven SM, Holven KB (2017) Does dietary fat affect inflammatory markers in overweight and obese individuals?—a review of randomized controlled trials from 2010 to 2016. Genes Nutr 12(1). 10.1186/s12263-017-0580-4 10.1186/s12263-017-0580-4 PMC5628471 29043006

[25] Becker W (2007) Indikatorer för bra matvanor: resultat från intervjuundersökningar 2005 och 2006. Rapport / Livsmedelsverket, 1104–7089 ; 2007:3. Livsmedelsverket, Uppsala

[26] Wareham NJ, Jakes RW, Rennie KL, Schuit J, Mitchell J, Hennings S, Day NE (2003) Validity and repeatability of a simple index derived from the short physical activity questionnaire used in the European prospective investigation into Cancer and Nutrition (EPIC) study. Public Health Nutr 6(4):407–413. 10.1079/phn2002439 12795830 10.1079/PHN2002439

[27] Demmer E, Van Loan MD, Rivera N, Rogers TS, Gertz ER, German JB, Zivkovic AM, Smilowitz JT (2016) Consumption of a high-fat meal containing cheese compared with a vegan alternative lowers postprandial C-reactive protein in overweight and obese individuals with metabolic abnormalities: a randomised controlled cross-over study. J Nutr Sci 5:e9. 10.1017/jns.2015.1040eCollection 2016 27313852 10.1017/jns.2015.40 PMC4791521

[28] Schmid A, Petry N, Walther B, Butikofer U, Luginbuhl W, Gille D, Chollet M, McTernan PG, Gijs MA, Vionnet N, Pralong FP, Laederach K, Vergeres G (2015) Inflammatory and metabolic responses to high-fat meals with and without dairy products in men. Br J Nutr 113(12):1853–1861. 10.1017/s0007114515000677 25990454 10.1017/S0007114515000677 PMC4498462

[29] Arya F, Egger S, Colquhoun D, Sullivan D, Pal S, Egger G (2010) Differences in postprandial inflammatory responses to a ‘modern’ v. traditional meat meal: a preliminary study. Br J Nutr 104(5):724–728. 10.1017/s0007114510001042 20377925 10.1017/S0007114510001042

[30] Li Z, Wong A, Henning SM, Zhang Y, Jones A, Zerlin A, Thames G, Bowerman S, Tseng CH, Heber D (2013) Hass avocado modulates postprandial vascular reactivity and postprandial inflammatory responses to a hamburger meal in healthy volunteers. Food Funct 4(3):384–391. 10.1039/c2fo30226h 23196671 10.1039/c2fo30226h

[31] Svelander C, Gabrielsson BG, Almgren A, Gottfries J, Olsson J, Undeland I, Sandberg AS (2015) Postprandial lipid and insulin responses among healthy, overweight men to mixed meals served with baked herring, pickled herring or baked, minced beef. Eur J Nutr 54(6):945–958. 10.1007/s00394-014-0771-3 25416681 10.1007/s00394-014-0771-3

[32] Turner KM, Keogh JB, Clifton PM (2016) Acute effect of red meat and dairy on glucose and insulin: a randomized crossover study. Am J Clin Nutr 103(1):71–76. 10.3945/ajcn.115.123505 26675776 10.3945/ajcn.115.123505

[33] Hjorth M, Galigniana NM, Ween O, Ulven SM, Holven KB, Dalen KT, Sæther T (2022) Postprandial effects of Salmon Fishmeal and Whey on metabolic markers in serum and gene expression in liver cells. Nutrients 14(8). 10.3390/nu14081593 10.3390/nu14081593 PMC9027870 35458155

[34] Furuta Y, Manita D, Hirowatari Y, Shoji K, Ogata H, Tanaka A, Kawabata T (2023) Postprandial fatty acid metabolism with coconut oil in young females: a randomized, single-blind, crossover trial. Am J Clin Nutr. 10.1016/j.ajcnut.2023.03.015 36948274 10.1016/j.ajcnut.2023.03.015

[35] Burdge GC, Powell J, Dadd T, Talbot D, Civil J, Calder PC (2009) Acute consumption of fish oil improves postprandial VLDL profiles in healthy men aged 50–65 years. Br J Nutr 102(1):160–165. 10.1017/s0007114508143550 19138437 10.1017/S0007114508143550

[36] Colhoun HM, Otvos JD, Rubens MB, Taskinen MR, Underwood SR, Fuller JH (2002) Lipoprotein subclasses and particle sizes and their relationship with coronary artery calcification in men and women with and without type 1 diabetes. Diabetes 51(6):1949–1956. 10.2337/diabetes.51.6.1949 12031985 10.2337/diabetes.51.6.1949

[37] Freedman DS, Otvos JD, Jeyarajah EJ, Barboriak JJ, Anderson AJ, Walker JA (1998) Relation of lipoprotein subclasses as measured by Proton nuclear magnetic resonance spectroscopy to coronary artery disease. Arterioscler Thromb Vasc Biol 18(7):1046–1053. 10.1161/01.atv.18.7.1046 9672064 10.1161/01.atv.18.7.1046

[38] Schaefer EJ (2002) Lipoproteins, nutrition, and heart disease. Am J Clin Nutr 75(2):191–212. 10.1093/ajcn/75.2.191 11815309 10.1093/ajcn/75.2.191

[39] Packard CJ (2003) Triacylglycerol-rich lipoproteins and the generation of small, dense low-density lipoprotein. Biochem Soc Trans 31(Pt 5):1066–1069. 10.1042/bst0311066 14505481 10.1042/bst0311066

[40] Bragg F, Kartsonaki C, Guo Y, Holmes M, Du H, Yu C, Pei P, Yang L, Jin D, Chen Y, Schmidt D, Avery D, Lv J, Chen J, Clarke R, Hill MR, Li L, Millwood IY, Chen Z (2022) The role of NMR-based circulating metabolic biomarkers in development and risk prediction of new onset type 2 diabetes. Sci Rep 12(1):15071. 10.1038/s41598-022-19159-8 36064959 10.1038/s41598-022-19159-8 PMC9445062

[41] Fernández-Cidón B, Candás-Estébanez B, Gil-Serret M, Amigó N, Corbella E, Rodríguez-Sánchez M, Padró-Miquel A, Brotons C, Hernández-Mijares A, Calmarza P, Jarauta E, Brea AJ, Mauri M, Guijarro C, Vila À, Valdivielso P, Corbella X, Pintó X (2021) Physicochemical Properties of Lipoproteins assessed by Nuclear Magnetic Resonance as a predictor of premature Cardiovascular Disease. PRESARV-SEA study. J Clin Med 10(7). 10.3390/jcm10071379 10.3390/jcm10071379 PMC8037702 33805580

[42] Melanson KJ, Greenberg AS, Ludwig DS, Saltzman E, Dallal GE, Roberts SB (1998) Blood glucose and hormonal responses to small and large meals in healthy young and older women. J Gerontol Biol Sci Med Sci 53(4):B299–305 10.1093/gerona/53a.4.b299 18314561

[43] Schwander F, Kopf-Bolanz KA, Buri C, Portmann R, Egger L, Chollet M, McTernan PG, Piya MK, Gijs MA, Vionnet N, Pralong F, Laederach K, Vergeres G (2014) A dose-response strategy reveals differences between normal-weight and obese men in their metabolic and inflammatory responses to a high-fat meal. J Nutr 144 (10):1517–1523. doi: 1510.3945/jn.1114.193565. Epub 192014 May 193568 10.3945/jn.114.193565 PMC4162475 24812072

[44] Monsonis Centelles S, Hoefsloot HCJ, Khakimov B, Ebrahimi P, Lind MV, Kristensen M, de Roo N, Jacobs DM, van Duynhoven J, Cannet C, Fang F, Humpfer E, Schäfer H, Spraul M, Engelsen SB, Smilde AK (2017) Toward Reliable Lipoprotein particle predictions from NMR Spectra of Human blood: an Interlaboratory Ring Test. Anal Chem 89(15):8004–8012. 10.1021/acs.analchem.7b01329 28692288 10.1021/acs.analchem.7b01329 PMC5541326

[45] Haack M, Kraus T, Schuld A, Dalal M, Koethe D, Pollmacher T (2002) Diurnal variations of interleukin-6 plasma levels are confounded by blood drawing procedures. Psychoneuroendocrinology 27(8):921–931 12383453 10.1016/s0306-4530(02)00006-9

[46] Feingold KR, Grunfeld C (2000) Introduction to lipids and lipoproteins. In: De Groot LJ, Chrousos G, Dungan K et al (eds) Endotext. MDText.com, Inc., South Dartmouth (MA) 26247089

