# Supplementary material for: A randomized controlled cross-over trial investigating the acute inflammatory and metabolic response after meals based on red meat, fatty fish, or soy protein: the postprandial inflammation in rheumatoid arthritis (PIRA) trial
Source: Eur J Nutr. 2024 Jun 27;63(7):2631–42. doi: 10.1007/s00394-024-03451-6 (PMC11490451; doi:10.1007/s00394-024-03451-6)
Supplement: Supplementary file 1 — Supplementary Material 1 [file 394_2024_3451_MOESM1_ESM.docx]

**A randomized controlled cross-over trial investigating the acute inflammatory and metabolic response after meals based on red meat, fatty fish, or soy protein: The Postprandial Inflammation in Rheumatoid Arthritis (PIRA) trial**

**Supplemental tables**

**Supplemental Table 1**. Ingredients in the burgers served during meal challenges, standardized per 1 kg of red meat, fatty fish or soy protein.

|  | **Red Meat^1^** | **Fatty fish^2^** | **Soy protein^3^** | |
| --- | --- | --- | --- | --- |
| Batch (year) | 2019 & 2021 | 2019 & 2021 | 2019 | 2021 |
| Mince(g) | 1000 | 1000 | 1000 | 1000 |
| Egg (g) | 194 | 100 | 0 | 0 |
| Breadcrumbs (g) | 65 | 67 | 23 | 23 |
| Canola oil (g) | 0 | 0 | 65 | 0 |
| Salt (ml) | 8.5 | 8.5 | 1.5 | 1.5 |
| Pepper (ml) | 3.3 | 3.3 | 3.3 | 3.3 |
| Pre-cooked weight/burger (g) | 81 | 73 | 98 | 92 |

^1^ Red meat (60% beef and 40% pork) produced by Scan, by Swedish meat products.

^2.^ Farmed by Salmar Farming AS, Norway.

^3^ Soy based vegan substitute with product name *Anamma Formbar Färs,* produced by Orkla Foods, Sweden. Ingredients; water, soy protein (23%), canola oil, salt, spices, natural aromas, caramelized sugar, stabilizing agent (methylcellulose).

**Supplemental Table 2**. Nutritional content of the served meals

|  | Year 2019 batch | | | Year 2021 batch | | |
| --- | --- | --- | --- | --- | --- | --- |
|  | | | | | | |
| *Burgers* | Red meat^1^ | Fatty fish^2^ | Soy protein^3^ | Red meat^1^ | Fatty fish^2^ | Soy protein^3^ |
| Energy (kcal) | 323.7 | 347.1 | 405.0 | 336.5 | 326.7 | 300.7 |
| Protein^1^ (g) | 29.4 | 29.1 | 31.7 | 27.1 | 26.6 | 31.3 |
| Carbohydrate^2^ (g) | 5.9 | 5.9 | 5.6 | 5.9 | 5.9 | 5.6 |
| Fat^1^ (g) | 20.4 | 23.2 | 28.7 | 22.9 | 22.0 | 17.1 |
| Saturated fat (%)^5^ | 38.0 | 16.1 | 7.5 | 37.7 | 19.6 | 7.3 |
| Monounsaturated fat (%)^5^ | 46.6 | 49.2 | 58.5 | 49.4 | 41.5 | 64.4 |
| Polyunsaturated fat (%)^5^ | 8.5 | 31.6 | 32.9 | 8.9 | 34.1 | 27.3 |
| Eicosapentaenoic acid (%)^5^ | <0.1 | 2.8 | <0.1 | <0.1 | 3.5 | <0.1 |
| Docosahexaenoic acid (%)^5^ | <0.1 | 3.5 | <0.1 | 0.1 | 6.6 | <0.1 |
| *Bread and vegetables* | | | |  | | |
| Protein^2^ (g) | 7.5 | 7.5 | 7.5 | 7.5 | 7.5 | 7.5 |
| Fat^2^ (g) | 2.9 | 2.9 | 2.9 | 2.9 | 2.9 | 2.9 |
| Carbohydrate^2^ (g) | 39.7 | 39.7 | 39.7 | 39.7 | 39.7 | 39.7 |
| Energy (kcal) | 217.3 | 217.3 | 217.3 | 217.3 | 217.3 | 217.3 |
| *Hamburger dressing + canola oil* | | | |  | | |
| Protein^2^ (g) | 0.08 | 0.08 | 0.08 | 0.08 | 0.08 | 0.08 |
| Fat^2^ (g) | 17 | 14 | 9 | 15 | 15 | 21 |
| Carbohydrate^2^ (g) | 1.2 | 1.2 | 1.2 | 1.2 | 1.2 | 1.2 |
| Energy (kcal) | 155.4 | 128.9 | 84.7 | 137.7 | 137.7 | 190.8 |
| *Per total meal* | | | |  | | |
| Protein (g) | 37.0 | 36.7 | 39.3 | 34.7 | 34.2 | 38.8 |
| Fat (g) | 40.3 | 40.1 | 40.6 | 40.8 | 39.9 | 41.0 |
| Carbohydrate (g) | 46.8 | 46.8 | 46.4 | 46.8 | 46.8 | 46.4 |
| Energy (kcal) | 696 | 693 | 707 | 692 | 682 | 709 |

1 Red meat (60% beef and 40% pork) produced by Scan, by Swedish meat products.

2 Farmed by Salmar Farming AS, Norway.

3 Soy based vegan substitute with product name *Anamma Formbar Färs,* produced by Orkla Foods, Sweden.

4 Data analyzed by Eurofins Food & Feed Testing, Sweden.

5 Data calculated based on food labels, fiber content not included.

**Supplemental Table 3.** Metabolites quantified by ^1^H Nuclear Magnetic Resonance (NMR) analysis. Values presented are median (25p. 75p)

|  | Fasting (*n* = 23) | 30 min (*n* = 23) | 60 min (*n* = 23) | 120 min (*n* = 22) | 180 min (*n* = 22) | 300 min (*n* = 22) | Peak (*n* = 22)^1^ | Peak time (min)^2^ | Area under the curve (*n* = 22)^3^ |
| --- | --- | --- | --- | --- | --- | --- | --- | --- | --- |
| *Triglycerides (mg/dL)* | | | | | | | | |  |
| Soy protein | 83.68 (66.09. 104.42) | 91.45 (73.25. 119.98) | 94.02 (80.28. 131.96) | 122.66 (93.43. 153.29) | 126.11 (87.79. 154.58) | 108.67 (87.21. 127.86) | 137.39 (96.27. 159.89) | 180 (165. 180) | 6753 (4645. 12177) |
| Fatty fish | 76.6 (65.19. 91.54) | 89.68 (72.66. 97.3) | 98.7 (78.68. 118.7) | 113.34 (94.17. 136.81) | 113.91 (96.48. 134.6) | 96.87 (78.6. 125.11) | 125.19 (98.3. 146.16) | 180 (120. 180) | 7616 (5131. 11010) |
| Red meat | 77.61 (66.7. 102.31) | 79.75 (68.89. 108.07) | 92.74 (74.89. 110.56) | 109.32 (85.94. 122.22) | 111.5 (96.99. 138.95) | 100.12 (80.55. 137.59) | 125.36 (104.57. 150.28) | 180 (165. 300) | 8723 (6475. 9828) |
| P-value | 0.486 | 0.486 | 0.191 | 0.060 | 0.301 | 0.853 | 0.301 | 0.114 | 0.917 |
| *Cholesterol (mg/dL)* | | | | | | | | |  |
| Soy protein | 195 (177. 235) | 205 (176. 250) | 200 (169. 243) | 199 (172. 251) | 188 (163. 244) | 193 (161. 259) | 216 (185. 263) | 30 (23. 180) | 5709 (2783. 12824) |
| Fatty fish | 196 (155. 236) | 197 (161. 217) | 195 (173. 228) | 215 (157. 231) | 199 (174. 227) | 209 (165. 234) | 223 (181. 243) | 90 (30. 180) | 5532 (3075. 14466) |
| Red meat | 197 (164. 228) | 202 (165. 241) | 208 (170. 242) | 218 (172. 232) | 207 (178. 237) | 193 (160. 241) | 227 (186. 254) | 60 (30. 180) | 5144 (3304. 11980) |
| P-value | 0.339 | 0.486 | 0.799 | 0.590 | 0.470 | 0.301 | 0.470 | 0.682 | 0.917 |
| *LDL cholesterol (mg/dL)* | | | | | | | | |  |
| Soy protein | 102 (92. 139) | 118 (98. 147) | 111 (96. 140) | 110 (94. 151) | 106 (93. 154) | 106 (87. 142) | 121 (102. 158) | 45 (30. 120) | 3320 (1941. 7740) |
| Fatty fish | 113 (88. 131) | 111 (92. 138) | 105 (89. 148) | 112 (91. 142) | 108 (88. 126) | 107 (80. 128) | 121 (102. 152) | 30 (0. 120) | 3023 (2178. 8795) |
| Red meat | 113 (83. 138) | 112 (90. 146) | 121 (85. 150) | 113 (79. 145) | 111 (98. 139) | 106 (81. 146) | 127 (100. 155) | 60 (23. 135) | 3207 (1889. 6681) |
| P-value | 0.522 | 0.486 | 0.859 | 0.506 | 0.506 | 0.151 | 0.633 | 0.387 | 0.917 |
| *HDL cholesterol (mg/dL)* | | | | | | | | |  |
| Soy protein | 72 (60. 74) | 70 (59. 76) | 72 (56. 75) | 70 (64. 77) | 65 (55. 78) | 69 (60. 78) | 77 (68. 81) | 120 (30. 180) | 2237 (940. 4165) |
| Fatty fish | 69 (56. 73) | 67 (57. 75) | 69 (61. 74) | 69 (61. 77) | 68 (61. 74) | 66 (59. 80) | 72 (65. 84) | 60 (30. 180) | 1707 (938. 4578) |
| Red meat | 68 (60. 77) | 66 (58. 81) | 69 (65. 74) | 72 (62. 78) | 73 (62. 80) | 69 (56. 75) | 75 (66. 83) | 150 (30. 180) | 1594 (871. 4179) |
| P-value | 0.317 | 0.694 | 0.988 | 0.506 | 0.406 | 0.917 | 0.633 | 0.180 | 0.791 |
| *Apolipoprotein A1 (mg/dL)* | | | | | | | | |  |
| Soy protein | 164 (144. 176) | 165 (151. 175) | 159 (132. 171) | 162 (153. 172) | 158 (130. 175) | 163 (137. 179) | 174 (163. 185) | 30 (0. 180) | 2418 (1827. 8491) |
| Fatty fish | 153 (137. 175) | 150 (141. 173) | 154 (140. 167) | 156 (141. 178) | 153 (144. 170) | 154 (139. 179) | 172 (151. 183) | 60 (0. 180) | 3053 (2409. 9813) |
| Red meat | 159 (141. 171) | 157 (144. 177) | 161 (146. 169) | 164 (146. 174) | 163 (146. 180) | 159 (134. 176) | 170 (156. 194) | 60 (23. 180) | 3563 (1830. 7697) |
| P-value | 0.646 | 0.364 | 0.603 | 0.633 | 0.120 | 0.917 | 0.988 | 0.664 | 0.988 |
| *Apolipoprotein A2 (mg/dL)* | | | | | | | | |  |
| Soy protein | 31 (26. 36) | 32 (26. 35) | 31 (23. 35) | 30 (26. 34) | 29 (25. 34) | 31 (26. 36) | 33 (29. 38) | 30 (0. 210) | 1046 (651. 2016) |
| Fatty fish | 28 (25. 33) | 28 (25. 32) | 29 (26. 33) | 27 (26. 35) | 28 (25. 33) | 28 (25. 31) | 32 (28. 37) | 60 (23. 180) | 929 (657. 2284) |
| Red meat | 29 (24. 33) | 28 (25. 32) | 28 (25. 32) | 28 (26. 33) | 28 (27. 33) | 28 (25. 31) | 32 (29. 34) | 60 (23. 210) | 1028 (552. 1693) |
| P-value | 0.339 | 0.220 | 0.694 | 0.506 | 0.590 | 0.120 | 0.151 | 0.638 | 0.633 |
| *Apolipoprotein B100 (mg/dL)* | | | | | | | | |  |
| Soy protein | 76 (70. 111) | 83 (74. 112) | 79 (70. 108) | 82 (71. 114) | 78 (70. 111) | 79 (67. 115) | 89 (76. 117) | 45 (30. 210) | 1975 (1200. 3556) |
| Fatty fish | 79 (67. 99) | 78 (70. 96) | 76 (69. 99) | 80 (68. 102) | 80 (66. 98) | 77 (67. 99) | 85 (75. 109) | 45 (0. 120) | 1901 (993. 4789) |
| Red meat | 81 (66. 99) | 88 (68. 100) | 88 (66. 100) | 86 (65. 102) | 85 (74. 102) | 82 (68. 101) | 92 (76. 108) | 120 (60. 180) | 1868 (1145. 3796) |
| P-value | 0.603 | 0.603 | 0.799 | 0.683 | 0.069 | **0.034** | 0.120 | 0.222 | 0.917 |
| *LDL / HDL cholesterol quote* | | | | | | | | |  |
| Soy protein | 1.81 (1.28. 2.31) | 1.86 (1.28. 2.34) | 1.84 (1.26. 2.33) | 1.81 (1.31. 2.35) | 1.73 (1.34. 2.31) | 1.68 (1.27. 2.22) | 1.88 (1.36. 2.39) | 30 (22.5. 60) | 16 (10. 31) |
| Fatty fish | 1.7 (1.32. 2) | 1.64 (1.41. 2.39) | 1.64 (1.37. 2.38) | 1.62 (1.32. 2.34) | 1.54 (1.26. 2.27) | 1.54 (1.18. 2.22) | 1.72 (1.39. 2.41) | 30 (0. 30) | 21 (13. 37) |
| Red meat | 1.55 (1.4. 2.29) | 1.61 (1.44. 2.31) | 1.58 (1.47. 2.34) | 1.62 (1.32. 2.26) | 1.59 (1.4. 2.23) | 1.52 (1.33. 2.24) | 1.69 (1.51. 2.37) | 0 (0. 37.5) | 16 (6. 35) |
| P-value | 0.954 | 0.527 | 0.424 | **0.048** | **0.018** | 0.291 | 0.794 | 0.148 | 0.988 |
| *Apolipoprotein-B100/Apolipoprotein-A1 quote* | | | | | | | | |  |
| Soy protein | 0.52 (0.45. 0.7) | 0.53 (0.44. 0.71) | 0.53 (0.45. 0.71) | 0.54 (0.44. 0.71) | 0.56 (0.45. 0.71) | 0.52 (0.43. 0.71) | 0.57 (0.45. 0.72) | 60 (30. 180) | 4 (3. 5) |
| Fatty fish | 0.52 (0.46. 0.71) | 0.52 (0.45. 0.7) | 0.52 (0.45. 0.7) | 0.52 (0.44. 0.71) | 0.51 (0.43. 0.69) | 0.5 (0.42. 0.69) | 0.53 (0.45. 0.72) | 30 (0. 120) | 4 (2. 6) |
| Red meat | 0.52 (0.46. 0.66) | 0.53 (0.46. 0.67) | 0.53 (0.47. 0.69) | 0.55 (0.46. 0.7) | 0.54 (0.48. 0.7) | 0.53 (0.46. 0.66) | 0.55 (0.48. 0.7) | 120 (60. 180) | 4 (3. 7) |
| P-value | 0.799 | 0.539 | 0.471 | 0.671 | **0.019** | **0.016** | 0.748 | **0.001** | 0.623 |
| *Total particle number (nmol/L)* | | | | | | | | |  |
| Soy protein | 1384 (1267. 2012) | 1513 (1337. 2029) | 1442 (1264. 1967) | 1487 (1284. 2065) | 1425 (1275. 2020) | 1442 (1218. 2092) | 1613 (1380. 2120) | 45 (30. 210) | 35922 (21830. 64665) |
| Fatty fish | 1433 (1215. 1806) | 1413 (1270. 1750) | 1377 (1248. 1791) | 1462 (1243. 1856) | 1457 (1204. 1775) | 1409 (1213. 1808) | 1538 (1370. 1977) | 45 (0. 120) | 34556 (18070. 87057) |
| Red meat | 1467 (1205. 1798) | 1601 (1237. 1813) | 1604 (1208. 1826) | 1564 (1181. 1861) | 1541 (1337. 1848) | 1495 (1242. 1845) | 1677 (1390. 1966) | 120 (60. 180) | 33963 (20830. 69020) |
| P-value | 0.603 | 0.603 | 0.799 | 0.683 | 0.069 | **0.034** | 0.120 | 0.222 | 0.917 |
| *VLDL particle number (nmol/L)* | | | | | | | | |  |
| Soy protein | 102 (82. 132) | 106 (81. 135) | 119 (78. 145) | 140 (91. 162) | 143 (100. 164) | 127 (87. 150) | 154 (104. 172) | 180 (165. 300) | 5535 (4575. 8372) |
| Fatty fish | 92 (75. 120) | 97 (75. 119) | 104 (82. 130) | 127 (97. 154) | 135 (109. 162) | 123 (88. 157) | 142 (109. 170) | 180 (180. 300) | 8020 (5355. 10520) |
| Red meat | 99 (69. 112) | 103 (64. 112) | 105 (71. 115) | 117 (72. 128) | 121 (83. 139) | 118 (87. 144) | 132 (89. 145) | 300 (180. 300) | 5915 (3430. 7656) |
| P-value | 0.274 | 0.141 | 0.132 | **0.013** | **0.007** | 0.258 | **0.027** | 0.251 | 0.051 |
| *IDL particle number (nmol/L)* | | | | | | | | |  |
| Soy protein | 64 (50. 119) | 64 (44. 88) | 60 (43. 95) | 67 (51. 97) | 67 (51. 96) | 76 (63. 110) | 82 (69. 123) | 120 (0. 300) | 5277 (3303. 9855) |
| Fatty fish | 51 (29. 86) | 52 (29. 77) | 53 (31. 77) | 63 (31. 90) | 58 (39. 83) | 66 (59. 102) | 66 (59. 102) | 300 (165. 300) | 5137 (3191. 10449) |
| Red meat | 60 (37. 90) | 62 (38. 78) | 62 (38. 82) | 57 (43. 87) | 64 (43. 85) | 67 (40. 92) | 75 (56. 98) | 30 (0. 210) | 4258 (2835. 7387) |
| P-value | 0.132 | 0.068 | 0.921 | 0.151 | 0.177 | 0.205 | 0.205 | **0.021** | 0.406 |
| *LDL particle number (nmol/L)* | | | | | | | | |  |
| Soy protein | 1340 (1080. 1597) | 1431 (1162. 1776) | 1345 (1144. 1695) | 1329 (1132. 1732) | 1297 (1073. 1678) | 1330 (1069. 1735) | 1478 (1192. 1798) | 30 (30. 75) | 24513 (16698. 65465) |
| Fatty fish | 1315 (1068. 1613) | 1314 (1152. 1556) | 1283 (1106. 1574) | 1336 (1129. 1582) | 1282 (1068. 1398) | 1296 (1002. 1505) | 1431 (1213. 1709) | 30 (0. 75) | 31123 (18115. 78663) |
| Red meat | 1350 (1016. 1577) | 1395 (1109. 1646) | 1431 (1062. 1709) | 1411 (992. 1636) | 1367 (1217. 1594) | 1299 (1064. 1632) | 1481 (1219. 1740) | 60 (23. 180) | 33934 (13444. 65070) |
| P-value | 0.694 | 0.274 | 0.799 | 0.323 | 0.177 | 0.470 | 0.791 | 0.205 | 0.917 |
| *LDL-1 particle number (nmol/L)* | | | | | | | | |  |
| Soy protein | 261 (214. 299) | 251 (180. 287) | 228 (182. 288) | 240 (193. 298) | 227 (177. 284) | 248 (187. 314) | 273 (209. 334) | 0 (0. 165) | 5760 (3303. 7948) |
| Fatty fish | 242 (191. 308) | 223 (185. 272) | 227 (194. 274) | 213 (175. 273) | 211 (175. 274) | 255 (195. 327) | 256 (209. 327) | 60 (0. 300) | 5904 (4257. 7394) |
| Red meat | 271 (190. 303) | 256 (186. 315) | 255 (184. 292) | 252 (184. 293) | 246 (185. 305) | 238 (193. 311) | 285 (195. 326) | 0 (0. 300) | 4345 (3203. 6485) |
| P-value | 0.422 | 0.603 | **0.011** | **< 0.001** | **0.001** | 0.348 | 0.853 | 0.413 | 0.470 |
| *LDL-2 particle number (nmol/L)* | | | | | | | | |  |
| Soy protein | 204 (160. 310) | 231 (178. 303) | 246 (214. 336) | 285 (236. 374) | 263 (230. 320) | 223 (197. 283) | 292 (251. 379) | 120 (120. 180) | 13776 (8077. 19118) |
| Fatty fish | 203 (159. 277) | 232 (180. 287) | 239 (208. 281) | 226 (200. 303) | 214 (185. 280) | 200 (143. 286) | 239 (217. 310) | 60 (60. 120) | 8703 (5322. 12025) |
| Red meat | 209 (175. 278) | 222 (193. 304) | 244 (194. 327) | 256 (189. 322) | 269 (202. 304) | 212 (189. 297) | 276 (214. 337) | 120 (60. 180) | 10254 (6778. 14743) |
| P-value | 0.603 | 0.317 | 0.091 | **< 0.001** | **< 0.001** | **< 0.001** | **< 0.001** | **< 0.001** | 0.095 |
| *LDL-3 particle number (nmol/L)* | | | | | | | | |  |
| Soy protein | 187 (156. 304) | 191 (157. 295) | 201 (165. 303) | 213 (168. 329) | 190 (167. 313) | 181 (140. 271) | 220 (185. 331) | 120 (120. 135) | 7783 (4360. 12088) |
| Fatty fish | 173 (149. 274) | 188 (146. 257) | 195 (161. 267) | 175 (140. 268) | 171 (127. 239) | 188 (139. 247) | 206 (165. 299) | 60 (0. 120) | 6253 (4682. 10207) |
| Red meat | 212 (144. 258) | 203 (138. 273) | 203 (138. 293) | 195 (132. 268) | 189 (138. 270) | 180 (130. 285) | 218 (157. 297) | 30 (0. 90) | 4351 (2455. 9506) |
| P-value | 0.603 | 0.364 | 0.274 | **0.005** | **< 0.001** | 0.205 | 0.081 | **0.013** | **0.013** |
| *LDL-4 particle number (nmol/L)* | | | | | | | | |  |
| Soy protein | 152 (97. 227) | 173 (92. 228) | 126 (71. 209) | 160 (62. 211) | 127 (79. 213) | 137 (72. 222) | 200 (97. 247) | 30 (0. 135) | 6418 (2225. 12078) |
| Fatty fish | 137 (102. 189) | 121 (77. 204) | 94 (73. 191) | 120 (50. 187) | 83 (44. 200) | 127 (65. 223) | 174 (108. 223) | 30 (0. 120) | 7423 (5392. 13398) |
| Red meat | 135 (87. 247) | 145 (80. 220) | 119 (59. 213) | 130 (57. 187) | 121 (56. 193) | 119 (33. 214) | 161 (95. 247) | 15 (0. 38) | 6245 (3125. 8834) |
| P-value | 0.433 | 0.151 | 0.470 | 0.108 | 0.108 | **0.034** | 0.593 | 0.512 | 0.761 |
| *LDL-5 particle number (nmol/L)* | | | | | | | | |  |
| Soy protein | 156 (125. 235) | 192 (142. 264) | 168 (100. 246) | 150 (110. 220) | 162 (96. 265) | 194 (122. 245) | 198 (156. 283) | 60 (0. 300) | 14240 (8919. 18272) |
| Fatty fish | 154 (88. 212) | 169 (127. 259) | 177 (126. 227) | 177 (119. 266) | 162 (114. 247) | 146 (84. 227) | 196 (156. 280) | 45 (30. 135) | 11825 (7156. 24946) |
| Red meat | 150 (100. 210) | 174 (97. 241) | 163 (104. 245) | 151 (96. 241) | 168 (130. 221) | 151 (102. 234) | 210 (140. 276) | 120 (30. 180) | 10559 (5015. 16760) |
| P-value | 0.068 | 0.317 | 0.486 | 0.120 | 0.506 | 0.120 | 0.258 | 0.605 | 0.590 |
| *LDL-6 particle number (nmol/L)* | | | | | | | | |  |
| Soy protein | 319 (267. 373) | 346 (304. 457) | 321 (227. 375) | 253 (186. 366) | 287 (201. 359) | 327 (284. 407) | 366 (319. 476) | 30 (30. 300) | 20572 (14725. 30610) |
| Fatty fish | 326 (243. 364) | 342 (257. 384) | 349 (284. 430) | 361 (286. 401) | 354 (262. 433) | 313 (254. 401) | 376 (331. 480) | 120 (53. 180) | 17496 (10736. 30360) |
| Red meat | 298 (259. 368) | 337 (271. 398) | 329 (257. 391) | 351 (251. 413) | 367 (292. 416) | 337 (278. 405) | 389 (345. 463) | 180 (60. 180) | 16945 (10763. 31792) |
| P-value | 0.799 | 0.694 | 0.132 | **0.002** | **< 0.001** | 0.205 | 0.348 | 0.097 | 0.853 |
| *VLDL-triglycerides (mg/dL)* | | | | | | | | |  |
| Soy protein | 44 (29. 61) | 49 (37. 72) | 51 (39. 81) | 72 (45. 91) | 76 (48. 95) | 57 (42. 84) | 84 (50. 104) | 180 (165. 180) | 5915 (3895. 9259) |
| Fatty fish | 37 (29. 60) | 42 (31. 56) | 57 (36. 80) | 74 (52. 92) | 75 (50. 97) | 54 (36. 75) | 80 (55. 100) | 150 (120. 180) | 6088 (4372. 10553) |
| Red meat | 39 (24. 50) | 41 (31. 55) | 43 (35. 58) | 57 (34. 69) | 59 (50. 81) | 55 (36. 78) | 71 (50. 91) | 180 (165. 300) | 5795 (4471. 6897) |
| P-value | 0.603 | 0.220 | 0.091 | **0.021** | 0.120 | 0.791 | 0.120 | **0.013** | 0.129 |
| *IDL-triglycerides (mg/dL)* | | | | | | | | |  |
| Soy protein | 5.7 (2.9. 9) | 6.5 (3.8. 12.3) | 7.2 (5.6. 14.3) | 11.9 (7.2. 16.1) | 11.3 (5.8. 16.8) | 8.6 (5.8. 12.5) | 13.3 (7.5. 17.5) | 180 (120. 180) | 1039 (696. 1923) |
| Fatty fish | 3.8 (2.6. 7.2) | 6 (4.2. 8.2) | 8 (5. 13) | 10.1 (6.9. 15.6) | 9.6 (6.9. 13.4) | 6.7 (4.2. 11.5) | 11.7 (7.3. 15.9) | 120 (120. 180) | 1150 (792. 1951) |
| Red meat | 4.3 (3. 8.1) | 5.5 (3.6. 9.4) | 6.8 (4.5. 10.1) | 10 (6.1. 12.2) | 10.6 (8. 15.4) | 8.4 (5.3. 14) | 12.1 (9. 18) | 180 (120. 210) | 1587 (1083. 1730) |
| P-value | 0.537 | 0.636 | 0.422 | 0.095 | 0.590 | 0.332 | 1.000 | 0.299 | 0.988 |
| *LDL-triglycerides (mg/dL)* | | | | | | | | |  |
| Soy protein | 20.7 (16.5. 24) | 19.5 (15.1. 22.9) | 18.5 (14.9. 22.4) | 18.4 (14.5. 21.3) | 17.9 (12.7. 20.9) | 17.5 (13.4. 21.8) | 21.4 (17.6. 24.3) | 0 (0. 37.5) | 561 (262. 844) |
| Fatty fish | 18.6 (15.4. 21.8) | 17.5 (15. 21.9) | 17.9 (14.5. 20.3) | 16 (12.9. 19.6) | 14.5 (11.2. 19.5) | 17.5 (13.7. 22.8) | 20.5 (17. 23.7) | 0 (0. 30) | 704 (550. 1031) |
| Red meat | 18.9 (16.8. 23.7) | 20.1 (17.5. 21.8) | 19.9 (17.8. 21.8) | 19.7 (16.8. 22.7) | 20.1 (16.5. 21.3) | 18.7 (15.9. 21.9) | 20.5 (18.3. 25) | 60 (0. 180) | 414 (203. 819) |
| P-value | 0.422 | 0.164 | **0.009** | **0.004** | **< 0.001** | 0.348 | 0.120 | **0.013** | 0.051 |
| *HDL-triglycerides (mg/dL)* | | | | | | | | |  |
| Soy protein | 12.5 (8.8. 13.3) | 12.3 (8.4. 13.4) | 11.9 (8.9. 12.9) | 12.2 (9.8. 13.9) | 12.4 (9.6. 14.4) | 12.2 (9.2. 14.6) | 13.5 (10. 15.1) | 240 (120. 300) | 370 (236. 475) |
| Fatty fish | 11.2 (9.1. 13.2) | 11.1 (8.8. 11.8) | 10.8 (8.8. 11.6) | 11.3 (9.2. 12.4) | 11.5 (9. 12.4) | 11.7 (10.2. 14.2) | 12.4 (11.2. 14.2) | 300 (120. 300) | 320 (215. 670) |
| Red meat | 11.1 (8.8. 13.2) | 10.9 (8.8. 12.1) | 11.2 (9.5. 12.4) | 12 (10.5. 13.3) | 13.1 (10. 14.5) | 12.5 (9.3. 14.5) | 13.3 (11.6. 14.8) | 180 (180. 300) | 422 (311. 618) |
| P-value | 0.808 | 0.078 | 0.522 | 0.120 | **0.040** | 0.791 | 0.506 | 0.575 | 0.258 |
| *VLDL-Cholesterol (mg/dL)* | | | | | | | | |  |
| Soy protein | 10 (7. 14) | 11 (7. 15) | 12 (7. 16) | 15 (10. 20) | 16 (11. 20) | 16 (10. 18) | 18 (11. 22) | 180 (165. 300) | 970 (569. 1483) |
| Fatty fish | 9 (6. 14) | 10 (7. 12) | 10 (9. 14) | 15 (12. 19) | 17 (13. 20) | 15 (11. 19) | 17 (13. 23) | 180 (180. 300) | 1467 (1001. 1917) |
| Red meat | 10 (7. 13) | 10 (7. 13) | 11 (8. 14) | 13 (8. 15) | 15 (9. 17) | 13 (10. 17) | 16 (11. 18) | 180 (180. 300) | 931 (675. 1156) |
| P-value | 0.646 | 0.486 | 0.220 | **0.018** | **0.006** | 0.300 | **0.009** | 0.697 | **0.003** |
| *IDL-Cholesterol (mg/dL)* | | | | | | | | |  |
| Soy protein | 7.5 (3. 15.4) | 6.6 (3.8. 11.9) | 7.4 (4.1. 11.5) | 7.8 (5.8. 13.7) | 9 (4.8. 14) | 10.1 (6.1. 14.6) | 10.9 (7.4. 18.2) | 180 (0. 300) | 889 (482. 1497) |
| Fatty fish | 4.8 (1.6. 9.9) | 4.1 (0.8. 8.9) | 5.1 (2. 9) | 7.2 (2.9. 11.1) | 5.5 (3.6. 10.5) | 7.8 (5.3. 12.6) | 8.5 (5.8. 13.2) | 240 (105. 300) | 805 (480. 1720) |
| Red meat | 4.9 (3.2. 12) | 6.6 (2.8. 11.1) | 5.6 (2.4. 10.6) | 6.2 (2.9. 10.3) | 6.8 (2.6. 10) | 6.8 (2.4. 11.9) | 8.4 (4.9. 12.7) | 30 (0. 180) | 609 (456. 857) |
| P-value | 0.349 | 0.274 | 0.213 | **0.045** | **0.032** | **0.027** | **0.006** | **0.029** | 0.120 |
| *LDL cholesterol (mg/dL)* | | | | | | | | |  |
| Soy protein | 102 (92. 139) | 118 (98. 147) | 111 (96. 140) | 110 (94. 151) | 106 (93. 154) | 106 (87. 142) | 121 (102. 158) | 45 (30. 120) | 3320 (1941. 7740) |
| Fatty fish | 113 (88. 131) | 111 (92. 138) | 105 (89. 148) | 112 (91. 142) | 108 (88. 126) | 107 (80. 128) | 121 (102. 152) | 30 (0. 120) | 3023 (2178. 8795) |
| Red meat | 113 (83. 138) | 112 (90. 146) | 121 (85. 150) | 113 (79. 145) | 111 (98. 139) | 106 (81. 146) | 127 (100. 155) | 60 (23. 135) | 3207 (1889. 6681) |
| P-value | 0.522 | 0.486 | 0.859 | 0.506 | 0.506 | 0.151 | 0.633 | 0.387 | 0.917 |
| *HDL cholesterol (mg/dL)* | | | | | | | | |  |
| Soy protein | 72 (60. 74) | 70 (59. 76) | 72 (56. 75) | 70 (64. 77) | 65 (55. 78) | 69 (60. 78) | 77 (68. 81) | 120 (30. 180) | 2237 (940. 4165) |
| Fatty fish | 69 (56. 73) | 67 (57. 75) | 69 (61. 74) | 69 (61. 77) | 68 (61. 74) | 66 (59. 80) | 72 (65. 84) | 60 (30. 180) | 1707 (938. 4578) |
| Red meat | 68 (60. 77) | 66 (58. 81) | 69 (65. 74) | 72 (62. 78) | 73 (62. 80) | 69 (56. 75) | 75 (66. 83) | 150 (30. 180) | 1594 (871. 4179) |
| P-value | 0.317 | 0.694 | 0.988 | 0.506 | 0.406 | 0.917 | 0.633 | 0.180 | 0.791 |
| *VLDL-Free Cholesterol (mg/dL)* | | | | | | | | |  |
| Soy protein | 6 (4. 8) | 6 (4. 8) | 6 (5. 9) | 7 (5. 10) | 8 (6. 10) | 8 (6. 9) | 8 (6. 11) | 180 (180. 300) | 327 (267. 599) |
| Fatty fish | 6 (4. 7) | 6 (4. 7) | 6 (5. 8) | 8 (7. 10) | 8 (7. 10) | 8 (6. 10) | 9 (7. 11) | 180 (180. 300) | 599 (407. 720) |
| Red meat | 5 (5. 7) | 6 (4. 7) | 6 (4. 7) | 7 (5. 8) | 7 (6. 8) | 7 (6. 9) | 8 (6. 9) | 240 (180. 300) | 397 (286. 494) |
| P-value | 0.317 | 0.274 | 0.054 | **0.027** | **0.003** | 0.470 | **0.009** | 0.265 | **0.003** |
| *IDL-Free Cholesterol (mg/dL)* | | | | | | | | |  |
| Soy protein | 2 (1. 4) | 2 (1. 4) | 2 (1. 4) | 2 (2. 4) | 3 (2. 4) | 3 (2. 4) | 3 (2. 6) | 180 (0. 300) | 295 (163. 413) |
| Fatty fish | 1 (0. 3) | 1 (0. 3) | 2 (0. 3) | 2 (1. 4) | 2 (1. 3) | 2 (1. 4) | 2 (2. 4) | 180 (105. 300) | 241 (135. 532) |
| Red meat | 1 (1. 3) | 2 (1. 3) | 2 (1. 3) | 2 (1. 3) | 2 (1. 3) | 2 (1. 3) | 2 (1. 4) | 150 (23. 300) | 168 (134. 260) |
| P-value | 0.301 | 0.148 | 0.207 | **0.029** | 0.069 | 0.060 | 0.051 | 0.707 | 0.129 |
| *LDL-Free Cholesterol (mg/dL)* | | | | | | | | |  |
| Soy protein | 34 (28. 45) | 36 (29. 45) | 34 (29. 40) | 33 (28. 45) | 32 (26. 46) | 33 (26. 42) | 37 (29. 48) | 30 (0. 120) | 681 (372. 1855) |
| Fatty fish | 35 (28. 40) | 33 (28. 41) | 32 (29. 42) | 33 (28. 41) | 32 (27. 38) | 36 (25. 40) | 37 (29. 45) | 30 (0. 120) | 784 (564. 2282) |
| Red meat | 36 (28. 41) | 36 (29. 42) | 36 (26. 45) | 34 (24. 41) | 34 (28. 41) | 32 (25. 44) | 37 (30. 47) | 30 (0. 60) | 831 (525. 1648) |
| P-value | 0.799 | 0.522 | 0.799 | 0.791 | 0.301 | 0.177 | 0.988 | 0.571 | 0.791 |
| *HDL-Free Cholesterol (mg/dL)* | | | | | | | | |  |
| Soy protein | 17 (14. 19) | 17 (14. 20) | 16 (14. 19) | 17 (14. 20) | 16 (14. 19) | 17 (15. 19) | 18 (16. 21) | 180 (30. 300) | 336 (195. 820) |
| Fatty fish | 16 (14. 20) | 16 (14. 20) | 16 (15. 21) | 17 (15. 21) | 17 (16. 20) | 17 (15. 21) | 18 (16. 22) | 180 (120. 300) | 424 (171. 1011) |
| Red meat | 17 (16. 20) | 17 (16. 20) | 18 (15. 20) | 17 (16. 20) | 17 (15. 21) | 17 (15. 20) | 18 (16. 22) | 60 (23. 210) | 243 (138. 697) |
| P-value | 0.266 | 0.274 | 0.317 | 0.470 | 0.406 | 0.917 | 0.840 | 0.081 | 0.120 |
| *VLDL-Phospholipids (mg/dL)* | | | | | | | | |  |
| Soy protein | 13 (10. 18) | 14 (9. 20) | 15 (9. 20) | 16 (11. 22) | 19 (13. 24) | 17 (12. 20) | 19 (13. 25) | 180 (165. 300) | 794 (527. 1339) |
| Fatty fish | 12 (10. 16) | 12 (9. 17) | 14 (12. 19) | 19 (14. 22) | 19 (16. 24) | 18 (13. 22) | 21 (16. 25) | 180 (180. 210) | 1407 (1008. 1843) |
| Red meat | 13 (9. 15) | 13 (9. 16) | 13 (9. 16) | 14 (11. 17) | 16 (12. 19) | 16 (12. 21) | 18 (13. 21) | 240 (180. 300) | 855 (581. 1020) |
| P-value | 0.694 | 0.274 | **0.046** | **0.021** | **0.023** | 0.129 | **0.009** | 0.077 | **0.001** |
| *IDL-Phospholipids (mg/dL)* | | | | | | | | |  |
| Soy protein | 5.24 (2.79. 7.79) | 5.03 (3.19. 7.67) | 5.09 (2.66. 8.28) | 6.12 (3.57. 8.42) | 6.02 (3.63. 8.97) | 5.44 (4.11. 8.07) | 7.17 (5.1. 9.24) | 180 (30. 300) | 623 (311. 976) |
| Fatty fish | 3.72 (1.55. 5.98) | 4.15 (2.63. 5.78) | 4.79 (3.04. 6.83) | 5.7 (3.29. 8.28) | 5.29 (3.95. 7.08) | 5 (3.33. 8.51) | 6.48 (4.7. 8.94) | 150 (60. 210) | 542 (309. 1288) |
| Red meat | 4.2 (2.32. 6.86) | 5 (2.69. 6.73) | 5.08 (2.22. 7.78) | 5.01 (3.62. 7.82) | 5.88 (3.82. 7.89) | 5.86 (3.37. 7.14) | 6.66 (5.03. 9.33) | 180 (120. 300) | 538 (424. 871) |
| P-value | 0.466 | 0.364 | 0.954 | **0.032** | 0.406 | 0.395 | 0.120 | 0.598 | 0.917 |
| *LDL-Phospholipids (mg/dL)* | | | | | | | | |  |
| Soy protein | 62.6 (55.3. 79) | 66.7 (56.9. 81.4) | 65.5 (55.6. 75.4) | 64 (55.6. 83.3) | 62 (53.3. 85.1) | 62.6 (51.6. 79.8) | 69.7 (58.7. 86.7) | 30 (22.5. 60) | 1302 (869. 3817) |
| Fatty fish | 66.1 (52.8. 74.6) | 63.4 (54.4. 77.3) | 63.1 (54.5. 79.2) | 63.8 (54.4. 77) | 61.4 (53.1. 69.7) | 65.6 (49.8. 73.3) | 70.3 (59.4. 83.6) | 30 (22.5. 120) | 1487 (975. 3848) |
| Red meat | 66.9 (52.8. 78.7) | 66.6 (57. 81.4) | 69.1 (51.2. 82.1) | 67 (48. 78.1) | 64.6 (56.7. 76.9) | 62.2 (50.2. 81.3) | 71.7 (58. 85.8) | 30 (0. 135) | 1428 (1034. 2871) |
| P-value | 0.646 | 0.317 | 0.799 | 0.406 | 0.258 | 0.095 | 0.683 | 0.936 | 0.917 |
| *HDL-Phospholipids (mg/dL)* | | | | | | | | |  |
| Soy protein | 99.8 (87.1. 103.8) | 98.1 (87.9. 103.4) | 95.8 (83.5. 100) | 97.2 (88.4. 103.3) | 94.6 (77.5. 105.9) | 101.7 (88. 109.9) | 105.9 (95.5. 110.8) | 300 (30. 300) | 1744 (1431. 5120) |
| Fatty fish | 93.4 (79.6. 102.9) | 90.9 (78. 101) | 86.8 (80.2. 99.7) | 94.2 (82.1. 104.8) | 94.6 (84.2. 100) | 96.1 (82.3. 109.9) | 99.3 (91.1. 115.1) | 300 (105. 300) | 1957 (1440. 5558) |
| Red meat | 95.2 (85.3. 106.2) | 92.3 (80. 108.1) | 95.3 (89.9. 105.8) | 100.4 (84.5. 107.1) | 99.1 (84.6. 112.8) | 98 (82.8. 110.9) | 106.5 (91.3. 114.9) | 180 (0. 300) | 1590 (1325. 4376) |
| P-value | 0.220 | 0.339 | 0.486 | 0.406 | 0.301 | 0.470 | 0.177 | 0.365 | 0.470 |
| *HDL-Apo-A1 (mg/dL)* | | | | | | | | |  |
| Soy protein | 168.3 (147.5. 180.2) | 166.6 (156.8. 180.6) | 162.5 (138.4. 176.5) | 167.3 (158.7. 177.9) | 164.5 (136.2. 181.8) | 170.2 (142.4. 183.6) | 175.2 (167.3. 191.3) | 60 (30. 180) | 3020 (2204. 8891) |
| Fatty fish | 156.5 (141.6. 178.6) | 157.5 (141.5. 178.8) | 158.2 (143.7. 172) | 157.5 (145.3. 183.3) | 157.9 (146.9. 174.3) | 157.4 (142. 183.4) | 175.8 (156.4. 188.6) | 60 (22.5. 180) | 3219 (2281. 9751) |
| Red meat | 164.6 (141.1. 175.9) | 159.1 (144.9. 184.5) | 166.9 (146.1. 175.4) | 170 (147.4. 179) | 169.1 (151.3. 187.2) | 162.4 (139.6. 180.4) | 174.8 (155.3. 198.6) | 180 (30. 210) | 3520 (1506. 7904) |
| P-value | 0.646 | 0.603 | 0.422 | 0.633 | 0.060 | 0.791 | 0.791 | 0.705 | 0.590 |
| *HDL-Apo-A2 (mg/dL)* | | | | | | | | |  |
| Soy protein | 31.5 (27. 36.1) | 32.4 (26.2. 34.7) | 31.9 (23.7. 34.6) | 30.6 (26.7. 34.2) | 29.8 (25.6. 34.6) | 31.1 (26.5. 35.7) | 32.9 (29.8. 38) | 30 (0. 135) | 1053 (611. 1818) |
| Fatty fish | 28.9 (25.5. 33.7) | 28.8 (25.7. 32.2) | 29.3 (26.2. 33.2) | 28.1 (26.6. 34.5) | 28.4 (25.8. 33.4) | 28.4 (25.6. 31.7) | 32.7 (28.8. 36.9) | 60 (30. 180) | 855 (588. 2060) |
| Red meat | 29.7 (24.4. 33.3) | 28.5 (25.9. 32.6) | 28.4 (26.1. 32.1) | 29 (26.4. 33.4) | 28.7 (27.1. 33.2) | 28.4 (25.4. 31.8) | 32.7 (29.5. 34.8) | 60 (22.5. 180) | 961 (518. 1562) |
| P-value | 0.220 | 0.220 | 0.694 | 0.506 | 0.791 | 0.069 | 0.151 | 0.638 | 0.633 |
| *VLDL-Apo-B (mg/dL)* | | | | | | | | |  |
| Soy protein | 5.6 (4.5. 7.2) | 5.8 (4.4. 7.4) | 6.6 (4.3. 8) | 7.7 (5. 8.9) | 7.9 (5.5. 9) | 7 (4.8. 8.2) | 8.4 (5.7. 9.4) | 180 (165. 300) | 304 (252. 461) |
| Fatty fish | 5.1 (4.1. 6.6) | 5.3 (4.2. 6.6) | 5.7 (4.5. 7.2) | 7 (5.3. 8.5) | 7.4 (6. 8.9) | 6.8 (4.8. 8.6) | 7.8 (6. 9.4) | 180 (180. 300) | 442 (294. 579) |
| Red meat | 5.4 (3.8. 6.2) | 5.7 (3.6. 6.2) | 5.8 (3.9. 6.3) | 6.4 (3.9. 7) | 6.7 (4.6. 7.7) | 6.5 (4.8. 7.9) | 7.3 (4.9. 8) | 300 (180. 300) | 325 (189. 420) |
| P-value | 0.274 | 0.141 | 0.132 | **0.013** | **0.007** | 0.258 | **0.027** | 0.251 | 0.051 |
| *IDL-Apo-B (mg/dL)* | | | | | | | | |  |
| Soy protein | 3.5 (2.7. 6.6) | 3.5 (2.5. 4.8) | 3.3 (2.4. 5.2) | 3.7 (2.8. 5.4) | 3.7 (2.8. 5.3) | 4.2 (3.5. 6.1) | 4.5 (3.8. 6.8) | 120 (0. 300) | 290 (182. 542) |
| Fatty fish | 2.8 (1.6. 4.7) | 2.9 (1.6. 4.2) | 2.9 (1.7. 4.2) | 3.4 (1.7. 5) | 3.2 (2.1. 4.6) | 3.6 (3.2. 5.6) | 3.7 (3.3. 5.6) | 300 (165. 300) | 283 (175. 574) |
| Red meat | 3.3 (2. 5) | 3.4 (2.1. 4.3) | 3.4 (2.1. 4.5) | 3.2 (2.4. 4.8) | 3.5 (2.3. 4.7) | 3.7 (2.2. 5.1) | 4.1 (3.1. 5.4) | 30 (0. 210) | 234 (156. 406) |
| P-value | 0.132 | 0.079 | 0.921 | 0.151 | 0.177 | 0.205 | 0.194 | **0.021** | 0.406 |
| *LDL-Apo-B (mg/dL)* | | | | | | | | |  |
| Soy protein | 73.7 (59.4. 87.8) | 78.7 (63.9. 97.7) | 74 (62.9. 93.2) | 73.1 (62.2. 95.3) | 71.3 (59. 92.3) | 73.1 (58.8. 95.4) | 81.3 (65.6. 98.9) | 30 (30. 75) | 1348 (919. 3600) |
| Fatty fish | 72.3 (58.7. 88.7) | 72.3 (63.4. 85.6) | 70.6 (60.8. 86.6) | 73.5 (62.1. 87) | 70.5 (58.8. 76.9) | 71.3 (55.1. 82.8) | 78.7 (66.7. 94) | 30 (0. 75) | 1711 (997. 4326) |
| Red meat | 74.3 (55.9. 86.7) | 76.7 (61. 90.5) | 78.7 (58.4. 94) | 77.6 (54.5. 90) | 75.2 (67. 87.7) | 71.5 (58.5. 89.7) | 81.4 (67.1. 95.7) | 60 (22.5. 180) | 1866 (739. 3579) |
| P-value | 0.694 | 0.274 | 0.799 | 0.323 | 0.177 | 0.470 | 0.791 | 0.205 | 0.917 |
| *VLDL-1-Triglycerides (mg/dL)* | | | | | | | | |  |
| Soy protein | 18.9 (12.1. 25) | 23.4 (15.6. 29.1) | 27.4 (19.8. 47.1) | 46.7 (29.9. 60.8) | 49.7 (30.4. 64.9) | 30.9 (26. 57.1) | 57 (31.7. 67.6) | 180 (120. 180) | 5699 (3706. 8492) |
| Fatty fish | 17.6 (8.1. 25.7) | 21.5 (13.6. 29.1) | 30.4 (20.9. 44.6) | 37.3 (31.3. 58.5) | 41.8 (24.7. 52.8) | 22.4 (15.1. 36.6) | 43.4 (32.8. 62.2) | 120 (120. 180) | 4866 (3544. 7006) |
| Red meat | 15.2 (7.3. 21.7) | 18.2 (12.9. 27) | 22.1 (16.3. 28.6) | 32.1 (18.3. 43.4) | 35.5 (26.6. 46) | 30 (17.8. 46.8) | 39.3 (28.6. 56.4) | 180 (120. 300) | 4803 (3754. 5651) |
| P-value | 0.191 | 0.694 | **0.027** | **0.032** | **0.040** | **0.023** | **0.040** | **0.011** | **0.040** |
| *VLDL-2-Triglycerides (mg/dL)* | | | | | | | | |  |
| Soy protein | 7.2 (4.3. 9.5) | 8.3 (6.8. 11.8) | 8.5 (6.1. 11.5) | 8.5 (5.5. 12) | 8.4 (5.3. 11.8) | 7 (3.5. 9.4) | 9.7 (7.3. 14.4) | 120 (30. 210) | 773 (325. 1162) |
| Fatty fish | 5.4 (4.4. 9.3) | 7.6 (5.6. 10.5) | 9.1 (6.1. 12.5) | 10.7 (6.7. 15.1) | 11.9 (7. 15) | 8.3 (4.7. 12.8) | 13.3 (8.1. 16.3) | 180 (120. 210) | 983 (492. 1754) |
| Red meat | 6.1 (4.4. 7.8) | 6.4 (4.7. 9.3) | 6.7 (5.1. 9.9) | 7.1 (5.4. 11.9) | 8.9 (6.7. 13.1) | 8.4 (3.9. 11.7) | 11.7 (7. 13.9) | 180 (120. 210) | 799 (683. 1043) |
| P-value | 0.522 | 0.274 | 0.191 | **0.032** | 0.220 | 0.151 | **0.032** | 0.110 | 0.590 |
| *VLDL-3-Triglycerides (mg/dL)* | | | | | | | | |  |
| Soy protein | 6.1 (3.9. 8.5) | 6.1 (4.4. 9.3) | 6.3 (4.2. 9.1) | 7.4 (4.3. 9.1) | 6.4 (4.2. 10.2) | 5.7 (2.5. 7.8) | 8.4 (5.7. 11.4) | 60 (22.5. 180) | 616 (369. 739) |
| Fatty fish | 4.4 (3.2. 8.1) | 5.1 (4.2. 7.3) | 6.2 (4. 8.8) | 7.2 (5. 11.4) | 7.4 (5.4. 11.3) | 7.8 (4. 11.2) | 10.2 (6.5. 13.7) | 180 (120. 300) | 671 (418. 1377) |
| Red meat | 5.3 (3.6. 6.7) | 5 (3.4. 7.3) | 5.2 (4. 8.1) | 5.5 (2.8. 8.3) | 7 (4. 9) | 6.1 (3.3. 8.3) | 7.9 (5.2. 10.6) | 180 (60. 300) | 553 (322. 750) |
| P-value | 0.078 | 0.164 | 0.191 | **0.047** | 0.323 | **0.001** | **0.032** | 0.094 | 0.095 |
| *VLDL-4-Triglycerides (mg/dL)* | | | | | | | | |  |
| Soy protein | 5.9 (3.5. 8.8) | 5.7 (3.5. 7.8) | 6.8 (3.4. 7.6) | 6.7 (3.8. 8.8) | 6 (3.9. 10) | 6.9 (4.1. 8.8) | 8.1 (4.9. 10) | 150 (22.5. 300) | 333 (227. 395) |
| Fatty fish | 4.7 (3.7. 7.2) | 5 (3.7. 6.6) | 5.4 (3.9. 7) | 6 (5. 7.5) | 6.8 (5.3. 8.6) | 7 (5.5. 10.8) | 7.1 (6.7. 11.6) | 300 (180. 300) | 543 (325. 745) |
| Red meat | 6.2 (3.5. 6.8) | 6.2 (3.3. 6.9) | 5.9 (3.1. 6.9) | 5.6 (3.3. 6.8) | 6 (3.9. 6.9) | 6.3 (3.4. 7.5) | 6.9 (4.1. 8.3) | 240 (120. 300) | 189 (116. 371) |
| P-value | 0.522 | **0.016** | **0.046** | 0.069 | 0.258 | **0.004** | **0.011** | **0.038** | **< 0.001** |
| *VLDL-5-Triglycerides (mg/dL)* | | | | | | | | |  |
| Soy protein | 2.7 (2.3. 3.3) | 2.6 (2.3. 3) | 2.9 (2.5. 3.2) | 3 (2.5. 3.5) | 3.3 (2.8. 3.8) | 3.4 (2.9. 3.8) | 3.7 (3. 4) | 180 (165. 300) | 162 (104. 242) |
| Fatty fish | 2.5 (2.3. 3.3) | 2.6 (2.3. 3) | 2.7 (2.2. 3.1) | 3.2 (2.8. 3.4) | 3.3 (2.8. 3.7) | 3.4 (2.8. 3.9) | 3.7 (3. 4) | 180 (165. 300) | 212 (143. 260) |
| Red meat | 2.8 (2.4. 3) | 2.6 (2.3. 3.2) | 2.6 (2.3. 3.1) | 2.8 (2.3. 3) | 2.8 (2.4. 3.1) | 2.9 (2.4. 3.6) | 3.1 (2.8. 3.6) | 300 (97.5. 300) | 115 (50. 187) |
| P-value | 0.422 | 0.859 | 0.522 | **0.008** | **< 0.001** | 0.177 | **0.015** | 0.882 | **0.009** |
| *VLDL-1-Cholesterol (mg/dL)* | | | | | | | | |  |
| Soy protein | 3 (1.9. 4.2) | 3.1 (2. 4) | 4.1 (2.5. 5.1) | 6.1 (4.2. 8) | 6.8 (3.9. 8) | 4.8 (3.7. 8.3) | 7.3 (4.5. 9.4) | 180 (180. 180) | 619 (471. 1105) |
| Fatty fish | 3 (1.9. 3.8) | 3.1 (2. 3.9) | 3.5 (2.9. 4.3) | 5 (3.8. 7.2) | 5.7 (3.7. 7.3) | 4.1 (3.1. 5.5) | 5.8 (4.7. 8.2) | 180 (120. 180) | 616 (456. 787) |
| Red meat | 2.7 (1.9. 3.1) | 2.8 (1.9. 3.6) | 3.4 (2.4. 4.5) | 4.9 (3.5. 6.1) | 5.4 (4.7. 7.4) | 5 (3.3. 7.6) | 6.2 (4.8. 8.8) | 180 (165. 300) | 661 (567. 781) |
| P-value | 0.799 | 0.694 | 0.317 | 0.177 | 0.470 | 0.151 | 0.590 | 0.193 | 0.323 |
| *VLDL-2-Cholesterol (mg/dL)* | | | | | | | | |  |
| Soy protein | 1.6 (0.9. 2) | 1.8 (1.1. 2.3) | 1.3 (1.1. 2) | 1.2 (0.9. 1.7) | 1.2 (0.9. 1.8) | 1.3 (1. 1.8) | 1.8 (1.3. 2.5) | 30 (0. 300) | 122 (102. 198) |
| Fatty fish | 1.1 (1. 2) | 1.5 (1.1. 1.8) | 1.6 (1.2. 1.9) | 1.8 (1.3. 2.8) | 2.1 (1.5. 2.9) | 1.9 (1. 2.7) | 2.6 (1.8. 3.3) | 180 (165. 300) | 189 (102. 309) |
| Red meat | 1.3 (0.9. 1.8) | 1.3 (1. 1.7) | 1.6 (1. 2) | 1.8 (1.1. 2.3) | 1.8 (1.3. 2.7) | 1.7 (1.2. 2) | 2.1 (1.6. 2.7) | 180 (165. 300) | 159 (115. 227) |
| P-value | 0.636 | 0.141 | 0.606 | **0.009** | **< 0.001** | **0.002** | **0.002** | **0.002** | 0.506 |
| *VLDL-3-Cholesterol (mg/dL)* | | | | | | | | |  |
| Soy protein | 1.7 (0.4. 3) | 1.4 (0.6. 2.7) | 1.3 (0.3. 2.8) | 1.4 (0.7. 2.9) | 1.5 (0.8. 3.2) | 1.7 (0.9. 2.9) | 2.4 (1.4. 3.5) | 180 (0. 300) | 229 (98. 290) |
| Fatty fish | 1.1 (0.3. 2.7) | 1 (0.3. 1.4) | 1.2 (0.2. 2) | 1.5 (0.7. 2.7) | 2 (1. 3) | 2.2 (1.2. 3.6) | 2.6 (1.8. 4) | 300 (180. 300) | 273 (199. 522) |
| Red meat | 1.4 (0.5. 2.2) | 0.9 (0.4. 1.5) | 1.1 (0.5. 1.8) | 1.3 (0.4. 1.9) | 1.9 (0.7. 2.7) | 1.5 (0.9. 2.4) | 2.2 (1. 3) | 240 (165. 300) | 166 (110. 301) |
| P-value | 0.667 | **0.047** | 0.527 | 0.204 | 0.795 | **0.022** | **0.040** | **0.032** | 0.129 |
| *VLDL-4-Cholesterol (mg/dL)* | | | | | | | | |  |
| Soy protein | 3 (1.5. 5.1) | 3.2 (1.7. 4.8) | 2.7 (1.4. 4.3) | 2.9 (1.7. 5) | 3.1 (1.9. 5.5) | 3.9 (2.9. 6) | 4.4 (3.3. 6) | 300 (0. 300) | 235 (156. 331) |
| Fatty fish | 2.6 (1.5. 3.5) | 2.5 (1.9. 3.2) | 2.4 (2.1. 3.4) | 3 (2.3. 4.1) | 3.5 (2.8. 4.7) | 4.5 (3.1. 5.3) | 4.8 (3.4. 5.3) | 300 (180. 300) | 327 (201. 450) |
| Red meat | 3 (1.6. 4.2) | 3 (2.1. 4) | 2.8 (2.1. 3.7) | 2.9 (2. 3.6) | 3.1 (2.1. 4.1) | 3.4 (2.2. 4.7) | 3.7 (2.7. 4.8) | 300 (180. 300) | 168 (129. 209) |
| P-value | 0.486 | **0.022** | 0.316 | 0.060 | **0.032** | 0.081 | **0.047** | 0.144 | **0.001** |
| *VLDL-5-Cholesterol (mg/dL)* | | | | | | | | |  |
| Soy protein | 1.5 (1. 1.9) | 1.4 (1.1. 1.6) | 1.5 (1.4. 2.4) | 2 (1.4. 2.6) | 2.3 (1.6. 3) | 2.4 (2. 2.7) | 2.6 (2.1. 3) | 180 (180. 300) | 197 (146. 260) |
| Fatty fish | 1.4 (1.1. 1.9) | 1.4 (0.9. 1.8) | 1.5 (0.9. 1.9) | 2 (1.1. 2.3) | 2.1 (1.4. 2.4) | 2.1 (1.6. 2.6) | 2.4 (1.7. 2.7) | 180 (120. 300) | 183 (152. 249) |
| Red meat | 1.5 (0.9. 1.8) | 1.3 (1. 1.7) | 1.5 (1.1. 2) | 1.6 (1.1. 2.2) | 1.7 (1.3. 2.2) | 1.8 (1.5. 2.4) | 2.1 (1.7. 2.6) | 300 (120. 300) | 168 (91. 203) |
| P-value | 0.646 | 0.921 | 0.364 | 0.116 | **0.001** | **0.006** | **0.013** | 0.949 | 0.205 |
| *VLDL-1-Free Cholesterol (mg/dL)* | | | | | | | | |  |
| Soy protein | 1.1 (0.3. 1.8) | 1.5 (0.4. 1.9) | 1.5 (0.8. 2.8) | 2.7 (1.2. 3.7) | 2.8 (1.3. 3.9) | 2 (1.1. 3.4) | 3.3 (1.4. 4.1) | 180 (165. 180) | 306 (207. 443) |
| Fatty fish | 0.6 (0.2. 1.6) | 1 (0.6. 1.8) | 1.5 (0.8. 2.9) | 2.3 (1.4. 3.2) | 2.1 (1.1. 3.7) | 1.3 (0.7. 2.8) | 2.5 (1.5. 3.8) | 180 (120. 180) | 310 (212. 502) |
| Red meat | 0.7 (0.1. 1.4) | 1.1 (0.4. 1.9) | 1.1 (0.6. 2) | 1.9 (0.9. 2.6) | 2.1 (1.5. 3.1) | 1.7 (0.8. 3.3) | 2.6 (1.6. 3.6) | 180 (120. 300) | 308 (234. 381) |
| P-value | 0.622 | 0.316 | 0.236 | **0.032** | 0.406 | 0.177 | 0.427 | 0.408 | 0.633 |
| *VLDL-2-Free Cholesterol (mg/dL)* | | | | | | | | |  |
| Soy protein | 0.5 (0.2. 0.6) | 0.6 (0.2. 0.8) | 0.7 (0.2. 0.9) | 0.7 (0.3. 0.9) | 0.7 (0.4. 0.9) | 0.4 (0.1. 0.7) | 0.8 (0.4. 1.1) | 120 (60. 180) | 51 (29. 107) |
| Fatty fish | 0.3 (0.2. 0.5) | 0.4 (0.2. 0.6) | 0.6 (0.3. 1) | 0.7 (0.3. 1.2) | 0.7 (0.5. 1.3) | 0.6 (0.3. 1.1) | 1 (0.6. 1.5) | 180 (120. 300) | 89 (55. 150) |
| Red meat | 0.3 (0.3. 0.5) | 0.3 (0.2. 0.6) | 0.5 (0.3. 0.7) | 0.6 (0.3. 1) | 0.8 (0.4. 1.1) | 0.5 (0.3. 0.9) | 0.8 (0.4. 1.2) | 180 (120. 300) | 80 (59. 109) |
| P-value | 0.921 | 0.232 | 0.646 | 0.205 | **0.040** | **0.002** | 0.060 | **0.025** | 0.348 |
| *VLDL-3-Free Cholesterol (mg/dL)* | | | | | | | | |  |
| Soy protein | 0.5 (0.2. 1.1) | 0.6 (0.2. 1.1) | 0.8 (0.2. 1.3) | 1 (0.5. 1.7) | 1.2 (0.7. 1.7) | 0.9 (0.5. 1.5) | 1.6 (0.7. 1.9) | 180 (120. 300) | 119 (76. 162) |
| Fatty fish | 0.4 (0.2. 1) | 0.4 (0.2. 0.6) | 0.6 (0.3. 1.1) | 0.9 (0.6. 1.5) | 1 (0.6. 1.5) | 1 (0.5. 1.6) | 1.2 (0.8. 1.9) | 180 (165. 300) | 130 (96. 237) |
| Red meat | 0.6 (0.2. 0.8) | 0.4 (0.2. 0.7) | 0.4 (0.3. 0.9) | 0.6 (0.2. 1) | 1 (0.5. 1.2) | 0.8 (0.4. 1.2) | 1.2 (0.6. 1.4) | 180 (120. 300) | 94 (65. 144) |
| P-value | 0.141 | 0.155 | 0.220 | 0.069 | 0.111 | **0.018** | 0.080 | 0.574 | 0.120 |
| *VLDL-4-Free Cholesterol (mg/dL)* | | | | | | | | |  |
| Soy protein | 1.1 (0.1. 1.8) | 0.8 (0. 1.6) | 0.8 (0. 1.7) | 1.1 (0.3. 2.1) | 1.2 (0.6. 2.4) | 1.6 (0.8. 2.5) | 1.8 (0.9. 2.5) | 300 (180. 300) | 128 (73. 192) |
| Fatty fish | 0.4 (0. 1.3) | 0.5 (0.2. 1) | 0.6 (0.2. 1.2) | 1 (0.5. 1.6) | 1.5 (0.7. 1.9) | 1.4 (0.6. 2.3) | 1.8 (1.1. 2.3) | 300 (180. 300) | 196 (103. 296) |
| Red meat | 0.9 (0.1. 1.2) | 0.8 (0.2. 1.2) | 0.7 (0.1. 1.2) | 0.7 (0.1. 1.3) | 1 (0.3. 1.4) | 1 (0.4. 1.8) | 1.3 (0.5. 1.9) | 180 (120. 300) | 77 (61. 123) |
| P-value | 0.461 | **0.037** | 0.211 | **0.006** | **0.009** | **0.023** | **0.007** | 0.099 | **< 0.001** |
| *VLDL-5-Free Cholesterol (mg/dL)* | | | | | | | | |  |
| Soy protein | 0.7 (0.6. 1) | 0.5 (0.2. 0.6) | 0.4 (0.3. 0.7) | 0.5 (0.3. 0.6) | 0.7 (0.4. 0.9) | 0.9 (0.6. 1) | 1 (0.8. 1.3) | 240 (0. 300) | 76 (45. 120) |
| Fatty fish | 0.6 (0.4. 1) | 0.5 (0.2. 0.8) | 0.4 (0.1. 0.7) | 0.7 (0.2. 0.8) | 0.8 (0.4. 1) | 0.9 (0.7. 1.1) | 1 (0.7. 1.3) | 180 (105. 300) | 111 (62. 125) |
| Red meat | 0.7 (0.3. 0.9) | 0.5 (0.3. 0.6) | 0.5 (0.2. 0.7) | 0.6 (0.2. 0.9) | 0.6 (0.4. 0.8) | 0.8 (0.6. 1) | 0.9 (0.7. 1.2) | 300 (90. 300) | 73 (36. 107) |
| P-value | 0.724 | 0.878 | 0.944 | 0.473 | 0.097 | 0.665 | 0.385 | 0.571 | 0.258 |
| *VLDL-1-Phospholipids (mg/dL)* | | | | | | | | |  |
| Soy protein | 3.3 (1.8. 3.8) | 3.7 (2.4. 4.8) | 4 (3.5. 7.4) | 7.3 (4.4. 9.2) | 7.3 (4.3. 10.5) | 4.8 (3.8. 8.8) | 8.9 (5. 10.7) | 180 (120. 180) | 830 (572. 1253) |
| Fatty fish | 2.3 (1.7. 4.1) | 3.6 (2.3. 4.2) | 4.6 (3.1. 6.6) | 5.8 (4.7. 9) | 6.8 (3.9. 8.5) | 4.1 (2.6. 5.8) | 7 (4.8. 9.4) | 120 (120. 180) | 732 (489. 1061) |
| Red meat | 2.4 (1.7. 3.6) | 2.9 (2. 3.9) | 3.6 (2.6. 4.5) | 5.1 (3. 6.5) | 5.5 (4.2. 7.4) | 4.9 (2.9. 7.1) | 6.1 (4.5. 8.8) | 180 (120. 300) | 730 (490. 849) |
| P-value | 0.166 | 0.921 | 0.164 | 0.060 | 0.151 | 0.151 | 0.120 | 0.057 | 0.081 |
| *VLDL-2-Phospholipids (mg/dL)* | | | | | | | | |  |
| Soy protein | 1.9 (1.3. 2.7) | 2.2 (1.7. 3) | 2.1 (1.4. 3) | 2.1 (1.4. 2.9) | 2.1 (1.4. 3) | 1.9 (1.1. 2.6) | 2.5 (1.7. 3.7) | 90 (30. 210) | 193 (63. 261) |
| Fatty fish | 1.6 (1.2. 2.4) | 1.8 (1.4. 2.7) | 2.3 (1.4. 3.1) | 2.6 (1.5. 3.7) | 2.9 (1.6. 3.6) | 2.2 (1.4. 3.3) | 3.5 (2. 4.1) | 180 (120. 300) | 217 (108. 408) |
| Red meat | 1.6 (1.1. 2.3) | 1.6 (1.3. 2.5) | 1.6 (1.3. 2.4) | 1.9 (1.3. 3) | 2.3 (1.7. 3.4) | 2.3 (1.3. 3.1) | 2.9 (1.7. 3.6) | 180 (120. 300) | 222 (145. 251) |
| P-value | 0.132 | 0.236 | 0.184 | 0.129 | 0.205 | **0.018** | 0.060 | 0.068 | 1.000 |
| *VLDL-3-Phospholipids (mg/dL)* | | | | | | | | |  |
| Soy protein | 2.5 (0.8. 3.1) | 2.2 (1.1. 3) | 2.2 (0.8. 3.1) | 2.5 (1.3. 3.5) | 2.4 (1.4. 4) | 2.3 (1.4. 3.1) | 3.2 (1.7. 4.3) | 150 (22.5. 210) | 243 (99. 320) |
| Fatty fish | 1.2 (0.9. 2.7) | 1.5 (0.9. 2.3) | 2 (1. 2.8) | 2.3 (1.4. 3.7) | 2.6 (1.6. 3.9) | 2.8 (1.6. 4.1) | 3.1 (2.1. 4.6) | 180 (120. 300) | 288 (202. 555) |
| Red meat | 1.7 (0.9. 2.4) | 1.5 (1. 2.4) | 1.6 (0.8. 2.5) | 1.8 (0.8. 2.8) | 2.3 (1.2. 3.1) | 2.3 (1.1. 2.8) | 2.7 (1.4. 3.7) | 180 (180. 300) | 209 (143. 302) |
| P-value | 0.274 | 0.141 | 0.164 | 0.081 | 0.258 | **0.007** | 0.051 | 0.108 | 0.060 |
| *VLDL-4-Phospholipids (mg/dL)* | | | | | | | | |  |
| Soy protein | 3.2 (1.9. 4.4) | 3.3 (1.7. 4.2) | 3 (1.5. 4) | 3.1 (1.8. 4.3) | 3 (2.3. 5.1) | 3.9 (2.6. 5.1) | 4.2 (2.9. 5.1) | 180 (22.5. 300) | 170 (110. 214) |
| Fatty fish | 2.3 (1.9. 3.5) | 2.6 (2. 3.3) | 2.6 (2.1. 3.7) | 3.2 (2.7. 4) | 3.9 (2.9. 4.5) | 3.9 (2.9. 5.6) | 4.3 (3.5. 5.6) | 300 (180. 300) | 287 (195. 408) |
| Red meat | 3.1 (1.9. 3.5) | 3.1 (1.9. 3.6) | 2.9 (1.8. 3.6) | 2.9 (1.7. 3.4) | 3.2 (1.9. 3.7) | 3.4 (2. 4.2) | 3.6 (2.3. 4.4) | 240 (120. 300) | 113 (85. 168) |
| P-value | 0.236 | **0.032** | 0.191 | **0.001** | **0.009** | **0.003** | **0.002** | 0.076 | **< 0.001** |
| *VLDL-5-Phospholipids (mg/dL)* | | | | | | | | |  |
| Soy protein | 1.8 (1.3. 2) | 1.7 (1.3. 1.9) | 1.7 (1.4. 2.4) | 2.1 (1.6. 2.8) | 2.6 (1.7. 3.1) | 2.6 (2.2. 2.9) | 2.8 (2.3. 3.1) | 180 (180. 300) | 197 (141. 259) |
| Fatty fish | 1.5 (1.2. 2.1) | 1.5 (1.1. 1.9) | 1.7 (1.1. 2.1) | 2.3 (1.4. 2.5) | 2.4 (1.8. 2.8) | 2.4 (1.7. 2.9) | 2.7 (2.1. 3.1) | 240 (165. 300) | 213 (180. 299) |
| Red meat | 1.7 (1.1. 2.1) | 1.4 (1.1. 1.9) | 1.6 (1.2. 2.1) | 1.7 (1.2. 2.4) | 1.8 (1.5. 2.5) | 2 (1.8. 2.7) | 2.4 (1.8. 2.9) | 300 (120. 300) | 183 (106. 238) |
| P-value | 0.603 | 0.977 | 0.859 | 0.135 | **< 0.001** | 0.095 | 0.061 | 0.832 | 0.151 |
| *LDL-1-Triglycerides (mg/dL)* | | | | | | | | |  |
| Soy protein | 6 (5.2. 7.6) | 5.5 (5.2. 7.1) | 5.9 (5.4. 6.9) | 6 (5.4. 6.9) | 5.6 (4.8. 6.7) | 5.7 (4.5. 6.8) | 6.5 (5.6. 7.9) | 60 (0. 180) | 144 (112. 299) |
| Fatty fish | 6.2 (5.3. 7.1) | 5.7 (4.7. 6.3) | 5.4 (4.5. 6.1) | 4.5 (3.9. 5.4) | 4 (3.4. 5.3) | 5.6 (4.3. 6.8) | 6.6 (5.2. 7.3) | 0 (0. 75) | 285 (194. 313) |
| Red meat | 6.3 (5.2. 7.4) | 6 (5.1. 7) | 6.1 (5.4. 7.1) | 6.3 (5.6. 7.2) | 6.1 (5.2. 7.4) | 6.6 (5.3. 7.6) | 7.1 (5.9. 7.8) | 150 (52.5. 300) | 178 (122. 250) |
| P-value | 0.988 | 0.422 | **< 0.001** | **< 0.001** | **< 0.001** | 0.120 | **0.002** | **0.013** | **0.032** |
| *LDL-2-Triglycerides (mg/dL)* | | | | | | | | |  |
| Soy protein | 2.8 (2.2. 3.3) | 2.9 (2. 3.2) | 2.8 (2.1. 3.4) | 2.9 (2.3. 3.2) | 2.7 (1.9. 3) | 2.4 (1.9. 3.2) | 3.1 (2.3. 3.6) | 60 (0. 120) | 99 (58. 131) |
| Fatty fish | 2.7 (2. 3.3) | 2.5 (2. 3) | 2.6 (2.1. 3) | 2.4 (1.8. 2.8) | 2.2 (1.8. 2.7) | 2.7 (2. 3.6) | 3 (2.1. 3.6) | 180 (30. 300) | 114 (58. 144) |
| Red meat | 2.9 (2.1. 3.3) | 2.8 (2.2. 3.2) | 2.9 (2.1. 3.3) | 2.9 (2.3. 3.2) | 2.8 (2.2. 3.3) | 2.6 (2.1. 3.2) | 3.1 (2.3. 3.6) | 60 (0. 180) | 58 (34. 121) |
| P-value | 0.603 | 0.404 | 0.099 | **< 0.001** | **0.001** | **0.004** | 0.416 | 0.243 | 0.069 |
| *LDL-3-Triglycerides (mg/dL)* | | | | | | | | |  |
| Soy protein | 3.4 (2.6. 3.8) | 3.3 (2.7. 3.7) | 3.3 (2.5. 3.6) | 3.2 (2.4. 3.5) | 3.1 (2.4. 3.5) | 3.4 (2.7. 3.7) | 3.5 (2.8. 3.9) | 30 (0. 300) | 56 (38. 83) |
| Fatty fish | 3.2 (2.7. 3.6) | 3.1 (2.7. 3.6) | 3.1 (2.5. 3.6) | 3.1 (2.5. 3.5) | 3.1 (2.5. 3.5) | 3.2 (2.6. 3.8) | 3.4 (2.8. 3.8) | 15 (0. 300) | 41 (32. 51) |
| Red meat | 3.3 (2.7. 3.7) | 3.2 (2.8. 3.9) | 3.2 (2.7. 3.6) | 3.2 (2.8. 3.8) | 3.2 (2.5. 3.8) | 3.3 (2.9. 3.9) | 3.4 (2.9. 4) | 120 (0. 300) | 40 (25. 53) |
| P-value | 0.081 | **0.034** | **0.027** | **0.002** | **0.012** | **0.047** | 0.153 | 0.331 | 0.120 |
| *LDL-4-Triglycerides (mg/dL)* | | | | | | | | |  |
| Soy protein | 2.2 (1.6. 3.2) | 2.1 (1.3. 3) | 1.5 (1.2. 3) | 1.6 (1. 2.7) | 1.5 (0.7. 2.6) | 1.8 (0.9. 2.7) | 2.4 (1.6. 3.3) | 0 (0. 30) | 95 (46. 152) |
| Fatty fish | 2.3 (1.6. 2.8) | 1.9 (1.3. 2.8) | 1.4 (1.1. 2.4) | 1.3 (0.5. 2.5) | 1.3 (0.4. 2.3) | 2.2 (1.2. 2.9) | 2.4 (1.6. 3.2) | 0 (0. 90) | 135 (94. 178) |
| Red meat | 2.3 (1.5. 3.1) | 1.9 (1.5. 2.7) | 1.8 (1.3. 2.6) | 1.7 (1.1. 2.3) | 1.7 (1.2. 2.4) | 1.5 (1.1. 2.7) | 2.4 (1.7. 3.1) | 0 (0. 37.5) | 78 (43. 132) |
| P-value | 0.482 | 0.699 | 0.242 | 0.120 | **0.045** | **0.040** | 0.586 | 0.804 | **0.013** |
| *LDL-5-Triglycerides (mg/dL)* | | | | | | | | |  |
| Soy protein | 2 (1.6. 3.4) | 2 (1.5. 3) | 1.6 (0.8. 2.7) | 1.4 (0.6. 2.5) | 1.3 (0.5. 2.4) | 1.6 (1. 2.9) | 2.2 (1.7. 3.4) | 30 (0. 30) | 162 (125. 198) |
| Fatty fish | 1.8 (1.4. 2.9) | 1.7 (1.3. 2.9) | 1.6 (1. 2.7) | 1.4 (0.8. 2.9) | 1.8 (0.8. 2.8) | 2.2 (1.2. 2.9) | 2.2 (1.5. 3.3) | 30 (0. 120) | 112 (83. 207) |
| Red meat | 1.9 (1.5. 3.3) | 2.1 (1.5. 2.8) | 1.9 (1.3. 2.8) | 1.7 (1.3. 2.8) | 1.9 (1.6. 3) | 2 (1.2. 2.9) | 2.3 (1.9. 3.3) | 30 (0. 180) | 114 (61. 191) |
| P-value | 0.603 | 0.603 | 0.329 | **0.040** | **< 0.001** | 0.104 | 0.791 | 0.669 | 0.177 |
| *LDL-6-Triglycerides (mg/dL)* | | | | | | | | |  |
| Soy protein | 4.4 (3.8. 5.2) | 4.8 (4.1. 6) | 4.2 (3.2. 5.1) | 3.6 (2.6. 5.2) | 4 (2.9. 5.1) | 4.4 (3.9. 6.1) | 4.8 (4.1. 6.2) | 30 (22.5. 300) | 234 (176. 301) |
| Fatty fish | 4.3 (3.5. 5.2) | 4.5 (3.6. 5.4) | 4.8 (4.2. 6) | 4.8 (4.2. 6.1) | 5.3 (3.7. 6.2) | 4.4 (3.7. 5.7) | 5.3 (4.6. 7) | 150 (60. 180) | 221 (111. 342) |
| Red meat | 4.3 (3.7. 5.4) | 4.7 (3.9. 5.6) | 4.7 (3.8. 5.7) | 4.8 (3.7. 6) | 5.3 (4.3. 6.1) | 4.9 (4. 5.6) | 5.5 (4.6. 6.7) | 180 (105. 210) | 220 (101. 355) |
| P-value | 1.000 | 0.603 | **0.037** | **0.003** | **< 0.001** | 0.470 | **0.047** | 0.179 | 0.988 |
| *LDL-1-Cholesterol (mg/dL)* | | | | | | | | |  |
| Soy protein | 23.4 (19.7. 31.9) | 24.7 (17.8. 31.3) | 22.3 (17.9. 29.6) | 23.6 (18.7. 31.6) | 21.3 (16.8. 30.5) | 24.1 (18.1. 33) | 26.2 (20.2. 34) | 0 (0. 165) | 584 (388. 1014) |
| Fatty fish | 22.7 (19.2. 32.6) | 22 (18. 29) | 22.6 (18. 28.1) | 21.1 (16.8. 29.3) | 19.6 (16.9. 28.9) | 23.8 (19. 34.7) | 24.1 (20.3. 34.7) | 90 (0. 300) | 621 (417. 833) |
| Red meat | 26.9 (18.7. 32.8) | 25.7 (17.8. 32.1) | 25.1 (17.2. 31.7) | 25 (17.2. 31.2) | 24.6 (17.2. 30.9) | 22.7 (18.3. 32.2) | 28.3 (18.7. 34.7) | 0 (0. 165) | 512 (305. 773) |
| P-value | 0.646 | 0.317 | 0.164 | **0.007** | 0.081 | 0.470 | 0.917 | 0.091 | 0.095 |
| *LDL-2-Cholesterol (mg/dL)* | | | | | | | | |  |
| Soy protein | 21 (15.2. 33.1) | 24.4 (16.8. 31.5) | 25.8 (20.4. 34.6) | 28.9 (22.9. 37.6) | 26.6 (21.8. 33.4) | 22.3 (19.4. 28.9) | 30.4 (24.1. 38.3) | 120 (120. 180) | 1629 (922. 2028) |
| Fatty fish | 19.3 (14.4. 28.5) | 22.8 (18.9. 29.4) | 24.1 (20.5. 29.4) | 22.8 (20.2. 31.2) | 21.7 (17.8. 28.5) | 19.8 (14.1. 28.4) | 23.9 (21.6. 32) | 60 (60. 120) | 993 (647. 1310) |
| Red meat | 20.6 (17. 28.6) | 23.1 (18.9. 31.8) | 25.5 (18.5. 33.4) | 25.3 (18.1. 33.5) | 26.6 (20.2. 30.8) | 20.3 (17.9. 30.3) | 27.5 (20.4. 35.1) | 120 (60. 180) | 1155 (660. 1604) |
| P-value | 0.694 | 0.274 | 0.274 | **0.003** | **< 0.001** | **0.003** | **0.003** | **< 0.001** | 0.177 |
| *LDL-3-Cholesterol (mg/dL)* | | | | | | | | |  |
| Soy protein | 16.5 (13.5. 29.8) | 17.1 (13.3. 29.6) | 18.5 (15.2. 30.1) | 19.8 (15.4. 32.7) | 17.5 (14.9. 30.7) | 16.3 (12.4. 26.2) | 20.3 (15.9. 32.9) | 120 (60. 120) | 844 (432. 1331) |
| Fatty fish | 15.5 (12.9. 26.1) | 17.9 (13.6. 24.3) | 18.2 (14.7. 27) | 16.3 (11.8. 25.8) | 15.3 (11.6. 23.7) | 16 (12.1. 23.7) | 18.7 (15.3. 28.7) | 60 (22.5. 120) | 638 (480. 962) |
| Red meat | 18.7 (14.3. 24.5) | 19.1 (12.5. 26.8) | 20.3 (13.2. 28.6) | 17.9 (12.1. 26) | 17.4 (11.8. 26.2) | 16.1 (11.9. 27.5) | 20.4 (13.9. 29) | 30 (0. 60) | 455 (231. 919) |
| P-value | 0.522 | 0.646 | 0.274 | **0.004** | **0.001** | 0.301 | 0.151 | **0.012** | **0.040** |
| *LDL-4-Cholesterol (mg/dL)* | | | | | | | | |  |
| Soy protein | 13.4 (8.9. 20.2) | 15.8 (7.8. 20.8) | 11.4 (7.5. 19.8) | 15 (6.3. 20.4) | 11.5 (7. 20.6) | 12 (6.5. 19.3) | 18.1 (8.8. 22.3) | 30 (0. 120) | 510 (322. 1117) |
| Fatty fish | 13.1 (8.5. 16.8) | 10.8 (7.7. 18) | 9.7 (6.8. 17.3) | 11.7 (4.2. 17) | 8.1 (4.1. 18) | 11.3 (4.7. 19) | 14.9 (9.6. 21.4) | 30 (0. 120) | 643 (410. 1145) |
| Red meat | 11.8 (7.7. 21.4) | 11.5 (6.4. 20.9) | 11 (4.9. 19.7) | 10.7 (4.8. 17.8) | 11.1 (4.2. 17.1) | 10.5 (2.2. 18.3) | 14.9 (8.2. 22.6) | 30 (0. 37.5) | 604 (255. 831) |
| P-value | 0.590 | 0.301 | 0.590 | 0.117 | 0.108 | **0.040** | 1.000 | 0.510 | 0.847 |
| *LDL-5-Cholesterol (mg/dL)* | | | | | | | | |  |
| Soy protein | 12 (9.3. 18.5) | 14.7 (10.2. 21.5) | 13.2 (6.5. 19.7) | 11.8 (8.6. 17.9) | 13.5 (7.3. 19.6) | 15 (9.4. 19.6) | 15.3 (12.1. 22.5) | 120 (30. 300) | 1181 (674. 1401) |
| Fatty fish | 12.1 (6.5. 16.1) | 13.4 (10.5. 20.8) | 13.5 (10.5. 19.1) | 13.7 (9.2. 20.8) | 12.6 (8.6. 19.4) | 11.3 (6.3. 18.1) | 15.4 (11.8. 22.9) | 60 (30. 135) | 1090 (559. 2015) |
| Red meat | 11.3 (7.7. 15.6) | 13.8 (8.1. 19) | 12 (7.7. 19.7) | 12.4 (7.8. 19.4) | 13.1 (9.5. 17.8) | 11 (7.1. 18.7) | 15.7 (10.5. 22.7) | 90 (30. 180) | 960 (383. 1420) |
| P-value | 0.132 | 0.486 | 0.317 | **0.032** | 0.506 | 0.220 | 0.506 | 0.339 | 0.470 |
| *LDL-6-Cholesterol (mg/dL)* | | | | | | | | |  |
| Soy protein | 21.8 (17.6. 25.2) | 23.2 (20.1. 30.1) | 21.4 (14.9. 25.9) | 17.4 (11. 24.5) | 18.6 (12.6. 23.5) | 21.2 (17.7. 28.2) | 24.3 (21.3. 32.8) | 30 (30. 30) | 1565 (974. 2218) |
| Fatty fish | 22.3 (15.8. 25.2) | 23.2 (17.3. 26.4) | 24.1 (18.9. 29.2) | 24.1 (19.1. 28) | 24.2 (18. 30.2) | 21.2 (17.4. 28.6) | 26.2 (22.6. 33) | 120 (52.5. 180) | 1179 (790. 2171) |
| Red meat | 19.6 (16.9. 24.3) | 22.1 (17.8. 27.1) | 21.8 (16.5. 26.9) | 22.2 (15.4. 27.3) | 22.9 (17.4. 27.9) | 21.7 (15.4. 26) | 25.1 (22. 31.7) | 150 (30. 180) | 945 (694. 2061) |
| P-value | 0.799 | 0.859 | 0.132 | **< 0.001** | **< 0.001** | 0.791 | 0.095 | **0.007** | 0.853 |
| *LDL-1-Free Cholesterol (mg/dL)* | | | | | | | | |  |
| Soy protein | 7.6 (6.1. 9.7) | 8 (5.1. 9.3) | 7 (5.5. 8.9) | 7.3 (5.3. 9.4) | 6.3 (4.7. 8.9) | 7 (5.2. 9.6) | 8.4 (6. 10) | 0 (0. 210) | 214 (123. 460) |
| Fatty fish | 7.4 (5.5. 9.6) | 6.6 (5. 8.2) | 6.7 (5.4. 8) | 6.4 (4.8. 8.2) | 6.3 (4.6. 8.3) | 7.4 (5.4. 10.1) | 7.7 (6.2. 10.2) | 90 (0. 300) | 221 (156. 291) |
| Red meat | 7.8 (6.5. 9.3) | 7.9 (5.3. 8.7) | 7.7 (5. 9.1) | 7.7 (5.4. 8.7) | 7.3 (5.7. 8.9) | 6.9 (5.3. 9.4) | 8.4 (6.2. 10) | 0 (0. 165) | 168 (105. 377) |
| P-value | 0.184 | 0.191 | 0.164 | **0.032** | **0.027** | 0.151 | 0.988 | 0.084 | 0.120 |
| *LDL-2-Free Cholesterol (mg/dL)* | | | | | | | | |  |
| Soy protein | 6.6 (4.4. 9.1) | 8.1 (4.7. 8.9) | 8.1 (5.5. 10) | 8.6 (6.7. 10.8) | 7.6 (6.6. 9.8) | 6.7 (5.8. 8.5) | 9.1 (7.4. 10.8) | 120 (120. 180) | 457 (261. 723) |
| Fatty fish | 6.7 (4.4. 8.5) | 6.9 (5.5. 8.3) | 7.3 (6.5. 8.7) | 6.7 (6. 8.8) | 6.7 (5.3. 8.3) | 6.2 (4.8. 8.2) | 7.4 (6.7. 9.3) | 60 (60. 120) | 286 (200. 472) |
| Red meat | 6.6 (5. 8.2) | 7.3 (5.7. 9.2) | 8 (5.7. 9.7) | 8 (5.9. 10.1) | 7.8 (6.3. 9) | 5.9 (5. 8.6) | 8.3 (6.3. 10.4) | 120 (60. 180) | 359 (225. 551) |
| P-value | 0.646 | 0.422 | 0.646 | **< 0.001** | **0.003** | **0.012** | **0.002** | **0.004** | 0.590 |
| *LDL-3-Free Cholesterol (mg/dL)* | | | | | | | | |  |
| Soy protein | 6 (4.6. 8.5) | 6.3 (4.5. 7.9) | 5.8 (4.9. 8) | 5.5 (4.7. 8.4) | 5.1 (4.3. 7.8) | 4.7 (3.8. 6.4) | 6.2 (5.1. 8.6) | 30 (0. 75) | 221 (141. 361) |
| Fatty fish | 6 (4.5. 7.4) | 6 (4.7. 7.5) | 5.6 (4.8. 7.6) | 4.9 (3.8. 7) | 4.6 (3.6. 6.3) | 5.4 (4. 6.8) | 6.2 (5.1. 8.4) | 0 (0. 37.5) | 245 (171. 318) |
| Red meat | 6.5 (4.9. 7.4) | 5.9 (4.5. 7.9) | 6 (4.5. 8.2) | 5.8 (3.8. 7.5) | 5.5 (3.8. 7.3) | 4.8 (3.8. 7) | 6.7 (4.8. 8.4) | 15 (0. 60) | 150 (109. 319) |
| P-value | 0.859 | 0.988 | 0.694 | **0.001** | **0.034** | 0.052 | 0.853 | 0.082 | 0.205 |
| *LDL-4-Free Cholesterol (mg/dL)* | | | | | | | | |  |
| Soy protein | 3.9 (3.3. 6.4) | 4.8 (3.4. 6) | 4.1 (3. 5.9) | 4.5 (2.9. 6) | 4 (2.5. 6.1) | 3.9 (2.6. 5.6) | 5.1 (3.6. 6.7) | 30 (0. 120) | 154 (75. 368) |
| Fatty fish | 4.2 (3.1. 5.5) | 3.6 (3.2. 5.7) | 3.7 (2.9. 5.8) | 4.2 (2. 5.7) | 3 (2. 5) | 3.9 (2.5. 5.6) | 4.9 (3.6. 6.3) | 15 (0. 75) | 188 (134. 365) |
| Red meat | 4.2 (2.9. 6.4) | 4.5 (2.5. 6.2) | 3.8 (2.6. 5.9) | 3.5 (2.2. 5.4) | 3.8 (2.1. 5.2) | 3.5 (1.3. 5.5) | 4.9 (2.8. 6.9) | 30 (0. 37.5) | 164 (93. 303) |
| P-value | 0.316 | 0.141 | 0.808 | 0.110 | 0.061 | **0.014** | 0.724 | 0.502 | 0.470 |
| *LDL-5-Free Cholesterol (mg/dL)* | | | | | | | | |  |
| Soy protein | 3.9 (3.1. 5.6) | 4.2 (3.5. 5.9) | 3.7 (2.3. 5.6) | 3.3 (2.5. 5) | 3.7 (1.9. 4.8) | 4 (2.9. 5.3) | 4.4 (3.8. 6.4) | 30 (0. 120) | 242 (173. 382) |
| Fatty fish | 3.9 (2.4. 5.1) | 4 (3. 5.6) | 3.9 (2.9. 5.4) | 3.6 (2.5. 5.6) | 3.2 (2.3. 4.7) | 3.5 (2.2. 4.8) | 4.3 (3.5. 6.1) | 30 (22.5. 120) | 195 (146. 524) |
| Red meat | 3.9 (2.8. 4.7) | 4.3 (2.8. 5.6) | 3.6 (2.6. 5.6) | 3.5 (2.2. 5.2) | 3.6 (2.7. 4.7) | 3.5 (2.2. 5.1) | 4.5 (3.3. 6.4) | 30 (0. 60) | 198 (114. 346) |
| P-value | 0.132 | 0.466 | 0.646 | 0.590 | 0.917 | 0.205 | 0.470 | 0.436 | 0.470 |
| *LDL-6-Free Cholesterol (mg/dL)* | | | | | | | | |  |
| Soy protein | 5.3 (4.6. 6.7) | 6.3 (5.5. 7.3) | 5.8 (4.4. 7) | 5.5 (3.6. 6.8) | 5.6 (3.7. 6.7) | 5.9 (4.9. 6.9) | 6.4 (5.8. 8.1) | 60 (30. 300) | 311 (223. 554) |
| Fatty fish | 5.9 (4.2. 6.7) | 5.9 (4.5. 7.3) | 6.3 (5.1. 7.4) | 6.2 (4.9. 7.4) | 6.1 (4.5. 7.2) | 5.4 (4.2. 6.5) | 7 (5.7. 8.5) | 60 (30. 180) | 301 (191. 671) |
| Red meat | 5 (4.3. 6.8) | 5.5 (4.7. 7.3) | 5.7 (4.3. 7.3) | 5.8 (4.2. 7.4) | 5.4 (4.8. 7.5) | 5.6 (4.6. 6.9) | 6.8 (5.4. 8.5) | 120 (52.5. 180) | 225 (179. 650) |
| P-value | 0.859 | 0.522 | 0.646 | 0.590 | 0.633 | 0.258 | 0.470 | 0.685 | 0.406 |
| *LDL-1-Phospholipids (mg/dL)* | | | | | | | | |  |
| Soy protein | 14 (12.1. 17.5) | 13.7 (10.7. 17) | 13.2 (10.8. 16.5) | 13.8 (11.1. 17.3) | 12.8 (10.3. 16.5) | 14.1 (10.7. 18.2) | 15.6 (12.4. 19.1) | 0 (0. 165) | 322 (173. 490) |
| Fatty fish | 13.7 (11.5. 17.8) | 13.1 (10.8. 15.9) | 13 (10.9. 16.1) | 12.1 (10.1. 16) | 11.6 (9.7. 16) | 14.1 (11.6. 19.2) | 14.4 (12.1. 19.2) | 60 (0. 300) | 322 (254. 413) |
| Red meat | 15.6 (11.2. 18.2) | 14.5 (10.8. 18.3) | 14.5 (10.7. 17.3) | 14.6 (10.5. 17.2) | 13.9 (10.8. 17.4) | 13.6 (10.9. 17.9) | 16.2 (11.2. 19) | 0 (0. 300) | 266 (168. 346) |
| P-value | 0.646 | 0.316 | 0.054 | **0.001** | 0.332 | 0.470 | 0.756 | 0.162 | 0.205 |
| *LDL-2-Phospholipids (mg/dL)* | | | | | | | | |  |
| Soy protein | 11.9 (8.6. 17.8) | 13.5 (9.3. 16.9) | 14 (11.1. 18.3) | 15.5 (12.8. 19.9) | 14.4 (12.5. 17.7) | 12.7 (11. 15.7) | 16.2 (13.4. 20.3) | 120 (120. 180) | 791 (477. 975) |
| Fatty fish | 10.9 (8.2. 15.4) | 12.4 (10.8. 15.5) | 13.4 (11.4. 15.8) | 12.6 (11.3. 16.6) | 12 (9.8. 15.3) | 11.4 (8.5. 15.5) | 13.3 (11.8. 17) | 60 (60. 120) | 448 (317. 579) |
| Red meat | 12.1 (9.6. 15.6) | 12.6 (10.5. 16.8) | 13.6 (10.2. 17.5) | 13.7 (10. 17.8) | 14.4 (10.9. 16.4) | 11.2 (9.9. 16.2) | 14.8 (11. 18.4) | 120 (60. 180) | 532 (325. 765) |
| P-value | 0.603 | 0.219 | 0.191 | **< 0.001** | **< 0.001** | **0.004** | **0.001** | **0.005** | 0.129 |
| *LDL-3-Phospholipids (mg/dL)* | | | | | | | | |  |
| Soy protein | 9.8 (8.2. 15.9) | 10 (7.7. 15.7) | 10.5 (9. 15.8) | 11.1 (9. 17.3) | 10 (8.8. 16.3) | 9.4 (7.6. 14.1) | 11.4 (9.3. 17.3) | 120 (60. 120) | 400 (237. 656) |
| Fatty fish | 9.1 (8. 14.2) | 10.1 (7.9. 13.4) | 10.3 (8.6. 14.2) | 9.3 (7.2. 13.9) | 8.8 (6.8. 12.8) | 9.5 (7.5. 13) | 10.6 (8.8. 15.6) | 60 (22.5. 120) | 319 (232. 480) |
| Red meat | 10.9 (8.1. 13.6) | 10.9 (7.5. 14.6) | 11.3 (7.6. 15.2) | 10.3 (7. 14.1) | 10 (7.2. 14.2) | 9.4 (7.1. 14.8) | 11.4 (8.1. 15.6) | 45 (0. 60) | 215 (133. 443) |
| P-value | 0.522 | 0.486 | 0.317 | **0.004** | **< 0.001** | 0.234 | 0.220 | **0.038** | **0.011** |
| *LDL-4-Phospholipids (mg/dL)* | | | | | | | | |  |
| Soy protein | 7.9 (5.3. 10.6) | 9 (5. 11.5) | 6.2 (4.3. 10.8) | 8.4 (3.7. 11) | 6.6 (4.5. 10.8) | 7 (4.2. 11) | 10 (5.3. 12.1) | 15 (0. 37.5) | 253 (129. 539) |
| Fatty fish | 7.5 (5.5. 9.6) | 6.5 (4.5. 10) | 5.6 (4.4. 9.7) | 6.6 (3.1. 9.5) | 5 (3. 10) | 6.6 (3.6. 10.7) | 8.7 (5.9. 11.7) | 30 (0. 135) | 324 (242. 577) |
| Red meat | 6.9 (5.1. 11.9) | 7.3 (4.5. 11.2) | 6.4 (3.6. 10.8) | 6.3 (3.4. 9.3) | 6.2 (2.9. 9.2) | 6 (1.7. 10.1) | 8.6 (5.2. 12.2) | 30 (0. 37.5) | 304 (147. 481) |
| P-value | 0.433 | 0.194 | 0.323 | 0.108 | 0.061 | 0.085 | 0.917 | 0.388 | 0.791 |
| *LDL-5-Phospholipids (mg/dL)* | | | | | | | | |  |
| Soy protein | 6.9 (5.9. 10.5) | 8.3 (6.3. 11.2) | 7.5 (4.6. 10.9) | 6.9 (5.4. 10.1) | 7.4 (4.5. 10.8) | 8.5 (5.7. 10.7) | 8.7 (7. 12.4) | 120 (30. 300) | 572 (312. 729) |
| Fatty fish | 7 (4.3. 9) | 7.7 (6.1. 11.4) | 7.6 (6.1. 10.1) | 8 (5.5. 11.6) | 7.3 (5.5. 10.8) | 6.4 (4.1. 10.3) | 8.6 (6.9. 12.2) | 60 (30. 135) | 540 (239. 980) |
| Red meat | 6.5 (4.9. 9.3) | 7.8 (4.8. 10.3) | 7.4 (4.8. 10.7) | 7.1 (4.8. 10.7) | 7.6 (5.6. 10) | 6.5 (4.7. 10.2) | 9.1 (6.2. 12) | 120 (30. 180) | 465 (245. 656) |
| P-value | **0.040** | 0.422 | 0.486 | 0.060 | 0.506 | 0.220 | 0.177 | 0.543 | 0.590 |
| *LDL-6-Phospholipids (mg/dL)* | | | | | | | | |  |
| Soy protein | 12.8 (10.5. 15) | 13.1 (12.1. 16.5) | 12.4 (9.7. 15.4) | 11.6 (7.8. 14.8) | 12.2 (8.4. 14.5) | 13 (11.4. 16.7) | 13.6 (12.8. 18.4) | 30 (30. 300) | 776 (554. 1034) |
| Fatty fish | 13.3 (10.3. 14.5) | 13.2 (10.3. 15.2) | 13.6 (11.8. 16.3) | 14.5 (11.8. 16.3) | 14.4 (10.6. 16.5) | 13 (10.9. 16) | 15.2 (13.5. 18.7) | 120 (52.5. 180) | 636 (366. 1153) |
| Red meat | 11.5 (10.2. 15.1) | 12.6 (10.6. 15.9) | 13.1 (10.1. 15.5) | 13.2 (9.8. 15.3) | 13.4 (11. 16.4) | 13.1 (10.2. 15.7) | 14.8 (12.5. 18) | 180 (60. 210) | 544 (327. 1136) |
| P-value | 0.694 | 0.859 | 0.274 | **0.003** | **0.011** | 0.688 | 0.095 | 0.258 | 0.791 |
| *LDL-1-Apo-B (mg/dL)* | | | | | | | | |  |
| Soy protein | 14.4 (11.8. 16.5) | 13.8 (9.9. 15.8) | 12.6 (10. 15.9) | 13.2 (10.6. 16.4) | 12.5 (9.7. 15.6) | 13.6 (10.3. 17.3) | 15 (11.5. 18.4) | 0 (0. 165) | 317 (182. 437) |
| Fatty fish | 13.3 (10.5. 16.9) | 12.3 (10.2. 15) | 12.5 (10.7. 15.1) | 11.7 (9.6. 15) | 11.6 (9.6. 15.1) | 14 (10.7. 18) | 14.1 (11.5. 18) | 60 (0. 300) | 324 (234. 406) |
| Red meat | 14.9 (10.4. 16.7) | 14.1 (10.2. 17.3) | 14 (10.1. 16.1) | 13.8 (10.1. 16.1) | 13.5 (10.2. 16.8) | 13.1 (10.6. 17.1) | 15.7 (10.7. 17.9) | 0 (0. 300) | 239 (176. 355) |
| P-value | 0.422 | 0.603 | **0.011** | **< 0.001** | **0.001** | 0.348 | 0.853 | 0.413 | 0.470 |
| *LDL-2-Apo-B (mg/dL)* | | | | | | | | |  |
| Soy protein | 11.2 (8.8. 17) | 12.7 (9.8. 16.7) | 13.5 (11.7. 18.5) | 15.7 (13. 20.6) | 14.4 (12.7. 17.6) | 12.3 (10.8. 15.6) | 16 (13.8. 20.8) | 120 (120. 180) | 757 (444. 1051) |
| Fatty fish | 11.2 (8.8. 15.2) | 12.7 (9.9. 15.8) | 13.1 (11.4. 15.5) | 12.5 (11. 16.7) | 11.7 (10.2. 15.4) | 11 (7.9. 15.7) | 13.2 (11.9. 17) | 60 (60. 120) | 478 (293. 661) |
| Red meat | 11.5 (9.7. 15.3) | 12.2 (10.6. 16.7) | 13.4 (10.7. 18) | 14.1 (10.4. 17.7) | 14.8 (11.1. 16.7) | 11.6 (10.4. 16.3) | 15.2 (11.8. 18.5) | 120 (60. 180) | 564 (372. 811) |
| P-value | 0.603 | 0.317 | 0.091 | **< 0.001** | **< 0.001** | **< 0.001** | **< 0.001** | **< 0.001** | 0.095 |
| *LDL-3-Apo-B (mg/dL)* | | | | | | | | |  |
| Soy protein | 10.3 (8.6. 16.7) | 10.5 (8.6. 16.2) | 11.1 (9.1. 16.7) | 11.7 (9.2. 18.1) | 10.5 (9.2. 17.2) | 10 (7.7. 14.9) | 12.1 (10.2. 18.2) | 120 (120. 135) | 428 (240. 665) |
| Fatty fish | 9.5 (8.2. 15.1) | 10.4 (8.1. 14.1) | 10.7 (8.8. 14.7) | 9.6 (7.7. 14.8) | 9.4 (7. 13.1) | 10.3 (7.6. 13.6) | 11.3 (9.1. 16.4) | 60 (0. 120) | 344 (258. 561) |
| Red meat | 11.7 (7.9. 14.2) | 11.2 (7.6. 15) | 11.2 (7.6. 16.1) | 10.7 (7.2. 14.7) | 10.4 (7.6. 14.9) | 9.9 (7.1. 15.7) | 12 (8.6. 16.3) | 30 (0. 90) | 240 (134. 523) |
| P-value | 0.603 | 0.364 | 0.274 | **0.005** | **< 0.001** | 0.205 | 0.081 | **0.013** | **0.013** |
| *LDL-4-Apo-B (mg/dL)* | | | | | | | | |  |
| Soy protein | 8.4 (5.4. 12.5) | 9.5 (5.1. 12.5) | 6.9 (3.9. 11.5) | 8.8 (3.4. 11.6) | 7 (4.3. 11.7) | 7.6 (4. 12.2) | 11 (5.3. 13.6) | 30 (0. 135) | 354 (123. 665) |
| Fatty fish | 7.5 (5.6. 10.4) | 6.7 (4.2. 11.2) | 5.2 (4. 10.5) | 6.6 (2.8. 10.3) | 4.6 (2.4. 11) | 7 (3.6. 12.3) | 9.6 (6. 12.3) | 30 (0. 120) | 409 (297. 738) |
| Red meat | 7.4 (4.8. 13.6) | 8 (4.4. 12.1) | 6.6 (3.3. 11.7) | 7.1 (3.1. 10.3) | 6.6 (3.1. 10.6) | 6.6 (1.8. 11.8) | 8.9 (5.2. 13.6) | 15 (0. 37.5) | 343 (172. 486) |
| P-value | 0.433 | 0.116 | 0.470 | 0.108 | 0.108 | **0.034** | 0.593 | 0.512 | 0.761 |
| *LDL-5-Apo-B (mg/dL)* | | | | | | | | |  |
| Soy protein | 8.6 (6.9. 12.9) | 10.6 (7.8. 14.5) | 9.3 (5.5. 13.5) | 8.3 (6. 12.1) | 8.9 (5.3. 14.6) | 10.6 (6.7. 13.5) | 10.9 (8.6. 15.6) | 60 (0. 300) | 783 (490. 1005) |
| Fatty fish | 8.5 (4.8. 11.7) | 9.3 (7. 14.3) | 9.7 (6.9. 12.5) | 9.8 (6.6. 14.6) | 8.9 (6.3. 13.6) | 8 (4.6. 12.5) | 10.8 (8.6. 15.4) | 45 (30. 135) | 650 (395. 1372) |
| Red meat | 8.2 (5.5. 11.6) | 9.6 (5.3. 13.2) | 9 (5.7. 13.5) | 8.3 (5.3. 13.3) | 9.2 (7.1. 12.2) | 8.3 (5.6. 12.8) | 11.5 (7.7. 15.2) | 120 (30. 180) | 580 (276. 922) |
| P-value | 0.068 | 0.317 | 0.486 | 0.120 | 0.506 | 0.120 | 0.258 | 0.605 | 0.590 |
| *LDL-6-Apo-B (mg/dL)* | | | | | | | | |  |
| Soy protein | 17.6 (14.7. 20.5) | 19 (16.7. 25.1) | 17.6 (12.5. 20.6) | 13.9 (10.2. 20.1) | 15.8 (11.1. 19.8) | 18 (15.6. 22.4) | 20.1 (17.5. 26.2) | 30 (30. 300) | 1132 (810. 1684) |
| Fatty fish | 17.9 (13.4. 20) | 18.8 (14.1. 21.1) | 19.2 (15.6. 23.6) | 19.8 (15.7. 22) | 19.5 (14.4. 23.8) | 17.2 (13.9. 22.1) | 20.7 (18.2. 26.4) | 120 (52.5. 180) | 962 (591. 1670) |
| Red meat | 16.4 (14.3. 20.3) | 18.5 (14.9. 21.9) | 18.1 (14.1. 21.5) | 19.3 (13.8. 22.7) | 20.2 (16. 22.9) | 18.5 (15.3. 22.3) | 21.4 (19. 25.5) | 180 (60. 180) | 932 (593. 1748) |
| P-value | 0.799 | 0.694 | 0.148 | **0.002** | **< 0.001** | 0.180 | 0.348 | 0.097 | 0.853 |
| *HDL-1-Triglycerides (mg/dL)* | | | | | | | | |  |
| Soy protein | 5.5 (4.6. 6.5) | 5.2 (4.3. 6.5) | 5.1 (3.8. 6.3) | 5 (4.4. 6.4) | 4.9 (4. 6.6) | 5.5 (3.5. 7.1) | 5.7 (4.8. 7.2) | 180 (0. 300) | 185 (100. 342) |
| Fatty fish | 5.1 (3.5. 6.2) | 4.5 (3.7. 5.7) | 4.6 (3.3. 6.1) | 4.8 (4. 5.7) | 4.8 (3.9. 5.8) | 5.7 (4. 7.2) | 6 (4.6. 7.2) | 300 (105. 300) | 241 (93. 428) |
| Red meat | 4.8 (3.7. 6) | 4.6 (3.6. 6.3) | 5 (4. 6.2) | 5.7 (4.6. 6.5) | 5.9 (4.5. 7.4) | 5.5 (4.1. 7.2) | 6 (5. 7.8) | 240 (165. 300) | 248 (174. 409) |
| P-value | 0.422 | 0.219 | 0.164 | **0.002** | **0.002** | 0.081 | **0.018** | 0.240 | 0.791 |
| *HDL-2-Triglycerides (mg/dL)* | | | | | | | | |  |
| Soy protein | 1.96 (1.32. 2.3) | 2.05 (1.25. 2.31) | 1.98 (1.3. 2.32) | 2.2 (1.62. 2.6) | 2.35 (1.72. 2.63) | 2.3 (1.68. 2.58) | 2.51 (1.79. 2.76) | 180 (120. 300) | 109 (70. 154) |
| Fatty fish | 1.69 (1.19. 2.07) | 1.73 (1.51. 1.93) | 1.72 (1.44. 2.1) | 1.88 (1.54. 2.12) | 1.83 (1.44. 2.3) | 2.13 (1.64. 2.36) | 2.14 (1.8. 2.4) | 300 (180. 300) | 97 (60. 149) |
| Red meat | 1.84 (1.24. 2.13) | 1.84 (1.24. 2) | 1.93 (1.31. 2.15) | 2.07 (1.58. 2.46) | 2.16 (1.57. 2.57) | 2.14 (1.4. 2.45) | 2.23 (1.84. 2.7) | 180 (165. 300) | 121 (86. 151) |
| P-value | 0.486 | 0.134 | 0.366 | **0.048** | **0.004** | 1.000 | 0.205 | 0.124 | 0.385 |
| *HDL-3-Triglycerides (mg/dL)* | | | | | | | | |  |
| Soy protein | 1.82 (1.47. 2.3) | 1.87 (1.52. 2.32) | 1.94 (1.52. 2.18) | 2.11 (1.64. 2.34) | 2.06 (1.74. 2.56) | 2.26 (1.62. 2.44) | 2.36 (1.77. 2.61) | 180 (120. 300) | 71 (40. 89) |
| Fatty fish | 1.62 (1.46. 2.02) | 1.71 (1.32. 1.9) | 1.71 (1.38. 2.02) | 1.78 (1.35. 2.17) | 1.67 (1.28. 2.13) | 1.78 (1.51. 2.16) | 1.88 (1.59. 2.25) | 300 (105. 300) | 59 (36. 90) |
| Red meat | 1.68 (1.41. 2.12) | 1.67 (1.3. 1.98) | 1.76 (1.41. 2.07) | 1.85 (1.61. 2.5) | 1.97 (1.52. 2.65) | 1.98 (1.45. 2.64) | 2.07 (1.76. 2.8) | 180 (180. 300) | 90 (64. 126) |
| P-value | 0.921 | 0.433 | 0.242 | **0.009** | **0.003** | 0.853 | **0.018** | 0.942 | 0.301 |
| *HDL-4-Triglycerides (mg/dL)* | | | | | | | | |  |
| Soy protein | 3.3 (2.07. 3.65) | 3.17 (2.24. 3.73) | 3.18 (2.41. 3.8) | 3.18 (2.14. 3.87) | 3.13 (2.18. 3.74) | 2.9 (2.31. 3.78) | 3.41 (2.49. 4) | 120 (0. 180) | 63 (43. 105) |
| Fatty fish | 2.87 (2.5. 3.68) | 2.86 (2.49. 3.5) | 2.81 (2.22. 3.55) | 2.74 (2.2. 3.66) | 2.67 (2.03. 3.3) | 2.68 (2.36. 3.33) | 3.09 (2.78. 3.73) | 30 (0. 120) | 64 (50. 115) |
| Red meat | 2.71 (2.39. 3.14) | 2.7 (2.32. 3.29) | 2.77 (2.34. 3.3) | 2.84 (2.28. 3.45) | 2.69 (2.21. 3.35) | 2.9 (2.36. 3.62) | 3.29 (2.75. 3.62) | 180 (30. 300) | 79 (46. 111) |
| P-value | 0.316 | 0.522 | 0.076 | 0.323 | 0.151 | 0.988 | 0.590 | **< 0.001** | 0.988 |
| *HDL-1-Cholesterol (mg/dL)* | | | | | | | | |  |
| Soy protein | 27.1 (24.1. 34.6) | 28.4 (22.9. 37.2) | 27.9 (21.5. 34.4) | 27.4 (22.2. 38.1) | 26.6 (20.2. 36.4) | 28.3 (20. 38.3) | 29.9 (25.9. 40.8) | 120 (22.5. 210) | 867 (510. 2657) |
| Fatty fish | 28.9 (18.5. 35.8) | 27.2 (20. 34.8) | 27.2 (21.3. 36.2) | 29.1 (22.2. 37.1) | 28.6 (22. 35) | 30.2 (22.5. 39.5) | 31 (25.1. 41.4) | 240 (52.5. 300) | 1051 (708. 2908) |
| Red meat | 29.9 (24.1. 37.1) | 29.1 (22.6. 37) | 30.4 (22.9. 35.2) | 32 (23. 38.8) | 33.6 (24.2. 41.6) | 30.9 (20.4. 38.3) | 36.3 (24.6. 43.4) | 180 (30. 300) | 844 (493. 2622) |
| P-value | 0.091 | 0.059 | 0.422 | 0.301 | **0.018** | **0.032** | 0.406 | 0.325 | 0.917 |
| *HDL-2-Cholesterol (mg/dL)* | | | | | | | | |  |
| Soy protein | 10.2 (8.2. 11.6) | 10.6 (8.6. 11.5) | 10.5 (7.8. 11.6) | 10.8 (9. 12.1) | 10.6 (7.6. 12.1) | 10.7 (8.4. 11.7) | 11.7 (10. 12.6) | 120 (52.5. 180) | 502 (199. 824) |
| Fatty fish | 9.9 (7.5. 11.5) | 9.3 (8.1. 11.2) | 9.9 (8.3. 11.3) | 9.9 (8.8. 11.7) | 9.4 (8.4. 11.6) | 10.9 (8.1. 12.2) | 11 (9.4. 12.8) | 120 (30. 300) | 404 (206. 845) |
| Red meat | 9.8 (8.2. 11.5) | 10.3 (7.9. 11.5) | 10.9 (8.7. 11.6) | 10.7 (8.4. 12) | 10.5 (8.9. 12.5) | 10 (7.6. 11.6) | 11.8 (9.5. 13) | 120 (60. 180) | 403 (208. 815) |
| P-value | 0.317 | 0.603 | 0.752 | 0.470 | 0.301 | 0.590 | 0.177 | 0.748 | 0.917 |
| *HDL-3-Cholesterol (mg/dL)* | | | | | | | | |  |
| Soy protein | 10.4 (9.4. 11.6) | 10.9 (9.1. 11.8) | 9.6 (8.4. 11.7) | 10.1 (9.2. 11.1) | 9.9 (8.3. 11.4) | 10.7 (9. 11.5) | 11.5 (9.8. 12.1) | 45 (0. 300) | 299 (143. 628) |
| Fatty fish | 10.2 (8.3. 11.8) | 9.4 (8.7. 10.9) | 9.7 (8.7. 10.5) | 9 (8.2. 10.9) | 9 (7.8. 10.5) | 9.7 (8.7. 11.1) | 10.6 (9.5. 12.5) | 90 (22.5. 300) | 259 (223. 611) |
| Red meat | 10.2 (8.8. 11.1) | 9.8 (8.8. 11.2) | 10.1 (8.7. 10.9) | 9.6 (8.7. 10.6) | 9.5 (8.7. 11.1) | 9.5 (8. 10.5) | 10.9 (9.8. 11.8) | 30 (0. 90) | 310 (119. 473) |
| P-value | 0.106 | 0.522 | 0.799 | 0.204 | 0.258 | 0.095 | **0.040** | 0.425 | 0.791 |
| *HDL-4-Cholesterol (mg/dL)* | | | | | | | | |  |
| Soy protein | 17.3 (16.7. 21.6) | 18.2 (16. 22) | 16.8 (15.5. 22.1) | 17.1 (15.3. 20.2) | 16.7 (14.9. 20.7) | 17.4 (14.7. 21.7) | 18.4 (17. 22.5) | 30 (30. 60) | 309 (196. 433) |
| Fatty fish | 17.9 (15.3. 21.3) | 17.9 (16.2. 21.7) | 18.2 (15.8. 21.4) | 18 (15.7. 22) | 17.9 (15. 19.8) | 16.5 (14. 18.3) | 19.5 (16.3. 23.1) | 45 (30. 120) | 419 (217. 575) |
| Red meat | 17.3 (15.2. 20.2) | 17.4 (15.9. 20.5) | 16.6 (15.8. 20) | 16.3 (15.2. 19.7) | 16.7 (14.7. 18.5) | 15.5 (13.8. 17.9) | 18.5 (16.5. 20.9) | 30 (22.5. 37.5) | 248 (114. 494) |
| P-value | 0.220 | 0.859 | 0.603 | 0.406 | 0.470 | **0.001** | 0.227 | 0.192 | 0.151 |
| *HDL-1-Free Cholesterol (mg/dL)* | | | | | | | | |  |
| Soy protein | 6.9 (5.9. 9) | 7 (5.2. 9.3) | 6.9 (4.8. 8.4) | 6.8 (5.1. 9.6) | 6.8 (4.6. 9) | 7 (5. 8.8) | 7.8 (6.5. 10.3) | 90 (30. 300) | 228 (141. 733) |
| Fatty fish | 6.7 (5. 8.7) | 6.2 (5.2. 8.4) | 6.6 (5.3. 8.7) | 7.2 (5.7. 8.8) | 6.8 (6. 8.3) | 7.1 (5.6. 9.2) | 7.4 (6.7. 9.7) | 180 (52.5. 300) | 321 (172. 811) |
| Red meat | 7 (5.8. 8.8) | 7.7 (5.7. 9) | 7.4 (6.2. 8.4) | 7.3 (6.1. 9.2) | 7.7 (5.8. 10.4) | 7.6 (4.8. 8.9) | 8.3 (6.8. 10.5) | 60 (30. 180) | 244 (134. 719) |
| P-value | 0.236 | 0.220 | 0.988 | 0.506 | 0.205 | 0.151 | 0.470 | 0.482 | 0.683 |
| *HDL-2-Free Cholesterol (mg/dL)* | | | | | | | | |  |
| Soy protein | 2.5 (2. 2.8) | 2.3 (2.2. 2.7) | 2.2 (1.6. 2.6) | 2.3 (2. 2.7) | 2.5 (1.6. 2.8) | 2.6 (1.9. 2.9) | 2.7 (2.4. 3.1) | 180 (0. 300) | 90 (69. 257) |
| Fatty fish | 2.2 (1.7. 2.7) | 2.1 (1.6. 2.5) | 2.1 (1.8. 2.5) | 2.2 (1.9. 2.6) | 2.2 (1.8. 2.5) | 2.4 (1.9. 2.8) | 2.6 (2.2. 3) | 180 (52.5. 300) | 131 (78. 293) |
| Red meat | 2.2 (1.9. 2.6) | 2.3 (1.8. 2.7) | 2.1 (1.8. 2.5) | 2.3 (1.8. 2.5) | 2.2 (1.9. 2.7) | 2.4 (1.7. 2.6) | 2.6 (2.2. 3) | 30 (0. 300) | 110 (58. 226) |
| P-value | 0.141 | 0.339 | 0.694 | 0.089 | 0.590 | 0.312 | 0.301 | 0.114 | 0.590 |
| *HDL-3-Free Cholesterol (mg/dL)* | | | | | | | | |  |
| Soy protein | 2.3 (2. 2.7) | 2.4 (2.1. 2.9) | 2.2 (1.8. 2.7) | 2.2 (2. 2.6) | 2.1 (1.6. 2.6) | 2.3 (2. 2.6) | 2.6 (2.2. 3) | 30 (0. 75) | 105 (58. 192) |
| Fatty fish | 2.2 (1.9. 2.5) | 2.2 (2. 2.5) | 2.2 (1.9. 2.5) | 2 (1.7. 2.7) | 1.9 (1.6. 2.3) | 2 (1.6. 2.3) | 2.4 (2.2. 3) | 30 (22.5. 60) | 83 (67. 219) |
| Red meat | 2.2 (2. 2.6) | 2.3 (1.9. 2.7) | 2.1 (1.8. 2.6) | 2.1 (1.8. 2.3) | 1.9 (1.6. 2.3) | 2 (1.6. 2.3) | 2.6 (2.1. 2.9) | 30 (0. 30) | 101 (41. 175) |
| P-value | 0.603 | 0.076 | 0.317 | 0.406 | 0.406 | **0.032** | 0.109 | 0.142 | 0.791 |
| *HDL-4-Free Cholesterol (mg/dL)* | | | | | | | | |  |
| Soy protein | 3.7 (2.9. 4.3) | 3.6 (3.1. 4.5) | 3.2 (2.4. 4.1) | 2.9 (2.3. 3.7) | 2.7 (2.3. 3.7) | 3.3 (2.4. 3.8) | 3.8 (3.2. 4.5) | 15 (0. 30) | 131 (70. 159) |
| Fatty fish | 3.6 (3.1. 4.3) | 3.4 (3.1. 4.1) | 3.3 (2.6. 3.9) | 2.8 (2.4. 3.7) | 2.6 (2.2. 3.2) | 2.8 (2.2. 3.5) | 3.6 (3.1. 4.5) | 30 (0. 60) | 110 (86. 148) |
| Red meat | 3.4 (3.1. 3.7) | 3.3 (2.7. 3.7) | 3 (2.5. 3.5) | 2.8 (2.2. 3.3) | 2.5 (2. 3) | 2.8 (2.2. 3.1) | 3.6 (3.3. 3.9) | 0 (0. 30) | 123 (56. 179) |
| P-value | 0.054 | 0.317 | 0.606 | 0.110 | 0.069 | 0.089 | 0.177 | 0.154 | 0.988 |
| *HDL-1-Phospholipids (mg/dL)* | | | | | | | | |  |
| Soy protein | 36.2 (29.5. 43.2) | 34.9 (29.4. 44.5) | 33.5 (29.3. 41.2) | 34 (29.3. 44.4) | 34.1 (25.9. 44.9) | 36 (28.2. 47.4) | 39.1 (34.3. 48.5) | 180 (52.5. 300) | 1039 (486. 3078) |
| Fatty fish | 34.8 (24.1. 43.7) | 33.2 (23.9. 41) | 31.9 (26.2. 43.7) | 35.6 (27.1. 44.8) | 34.4 (28.1. 43) | 38 (29.2. 50) | 39.2 (31.5. 50.4) | 300 (120. 300) | 1246 (858. 3546) |
| Red meat | 37.3 (28.6. 44) | 34.7 (27.9. 45) | 38.9 (28. 43.4) | 39.2 (27.4. 48.3) | 42.2 (30. 51.2) | 38.2 (26.7. 48.9) | 44.3 (30. 53.6) | 180 (52.5. 300) | 1104 (520. 2886) |
| P-value | 0.059 | 0.132 | 0.317 | 0.220 | **0.034** | **0.032** | 0.506 | 0.441 | 0.151 |
| *HDL-2-Phospholipids (mg/dL)* | | | | | | | | |  |
| Soy protein | 16.1 (14.3. 18.6) | 15.5 (13.4. 18.8) | 16.7 (12.5. 18) | 16.7 (14.4. 19.2) | 17 (13.3. 19.3) | 17.4 (13.9. 19.9) | 18.2 (15.1. 20) | 180 (105. 300) | 587 (370. 1055) |
| Fatty fish | 15.3 (12.6. 17.7) | 14.6 (12.8. 16.4) | 15.1 (13.2. 17.4) | 15.6 (14. 18.2) | 14.8 (13.5. 18.2) | 17.6 (13.8. 18.8) | 17.8 (15.1. 19.8) | 180 (52.5. 300) | 503 (349. 1144) |
| Red meat | 15 (13.6. 18.8) | 16.2 (13. 17.8) | 17 (13.6. 18) | 16.4 (13.7. 19.1) | 16.6 (14.4. 19.7) | 16.2 (13.7. 19) | 17.5 (15. 20.4) | 180 (60. 300) | 441 (315. 1126) |
| P-value | 0.078 | 0.078 | 0.603 | **0.040** | **0.011** | 0.976 | 0.470 | 0.761 | 0.406 |
| *HDL-3-Phospholipids (mg/dL)* | | | | | | | | |  |
| Soy protein | 17.5 (15.4. 18.6) | 17 (14.3. 19.1) | 15.9 (14.1. 17.9) | 16.9 (15.3. 18.7) | 17.1 (14.5. 18) | 18 (15.8. 19.6) | 18.3 (16.8. 20.3) | 240 (0. 300) | 464 (284. 842) |
| Fatty fish | 16.1 (14.5. 18.6) | 15.9 (14.8. 16.8) | 15.5 (14.5. 17.5) | 15.5 (13.9. 18.4) | 15.2 (13.6. 17.3) | 16.5 (15. 18.3) | 17.3 (15.8. 20.5) | 210 (22.5. 300) | 393 (300. 828) |
| Red meat | 16.3 (14.9. 18.1) | 15.9 (14.3. 17.8) | 16.1 (14.6. 17.6) | 16 (14.9. 18.2) | 16 (15.1. 18) | 16.8 (14.8. 18.4) | 17.6 (16.2. 19.6) | 180 (0. 300) | 426 (220. 678) |
| P-value | 0.191 | 0.068 | 0.184 | 0.069 | **0.045** | 0.081 | **0.013** | 0.926 | 0.348 |
| *HDL-4-Phospholipids (mg/dL)* | | | | | | | | |  |
| Soy protein | 26.7 (23.6. 30.1) | 26.8 (23.3. 29) | 25.5 (22.7. 28.5) | 25.5 (22.8. 29) | 25.3 (23. 28.1) | 27.5 (22.9. 29.8) | 28 (24.5. 30.7) | 75 (22.5. 300) | 395 (222. 567) |
| Fatty fish | 25.6 (23.8. 30.3) | 25.4 (23.9. 29) | 25.3 (22.8. 29.1) | 25.6 (22.8. 28.5) | 25.5 (22.2. 27.6) | 24.1 (21.8. 27.2) | 26.9 (23.7. 30.9) | 30 (0. 75) | 241 (155. 643) |
| Red meat | 25.3 (24. 27.7) | 25.5 (23.8. 27.2) | 25.4 (23.1. 26.7) | 24 (22.8. 27.2) | 24.5 (22.8. 27) | 25 (23.1. 27.1) | 26.5 (24.4. 28.9) | 30 (0. 75) | 245 (192. 402) |
| P-value | 0.274 | 0.646 | 0.888 | 0.301 | 0.323 | **0.002** | 0.590 | **0.029** | 0.323 |
| *HDL-1-Apo-A1 (mg/dL)* | | | | | | | | |  |
| Soy protein | 46.5 (36.5. 58) | 44 (37.1. 60.1) | 45.4 (36.7. 57.4) | 46.2 (37. 62.8) | 45.8 (33.7. 62.9) | 47.3 (35.8. 61.6) | 50.9 (42.8. 67.1) | 180 (105. 300) | 1821 (812. 4412) |
| Fatty fish | 46.1 (27.1. 58.5) | 44.9 (30.6. 55.8) | 41.7 (33.6. 58.7) | 46.1 (35.7. 60.7) | 44.8 (35.8. 58.5) | 48.7 (37.1. 66.8) | 50.6 (40.2. 67.3) | 300 (165. 300) | 1818 (1363. 4964) |
| Red meat | 48.2 (36. 57.9) | 47.4 (37.1. 59.2) | 50.2 (39.8. 58.3) | 52.7 (36.7. 63.9) | 57.2 (40.6. 70.5) | 49.8 (32.3. 66.2) | 58.3 (40.6. 71.4) | 180 (120. 300) | 1737 (1048. 4372) |
| P-value | **0.025** | 0.236 | 0.486 | 0.258 | **0.007** | 0.120 | 0.655 | 0.608 | 0.633 |
| *HDL-2-Apo-A1 (mg/dL)* | | | | | | | | |  |
| Soy protein | 25 (21.3. 26.7) | 24.6 (21.8. 27) | 23.3 (20.8. 25) | 23.4 (21.2. 26.5) | 24 (18.5. 26.8) | 25.4 (21.9. 27.4) | 26.4 (23.8. 28.2) | 300 (30. 300) | 439 (329. 1329) |
| Fatty fish | 23.1 (20.1. 26) | 21.8 (20. 25.1) | 22.1 (20. 24.9) | 23.5 (19.4. 25.8) | 22.9 (19.9. 25) | 23.4 (20.9. 26.5) | 24.6 (22.9. 28) | 240 (52.5. 300) | 519 (369. 1360) |
| Red meat | 22.8 (21. 27.4) | 22.8 (19.7. 26.7) | 23 (21.7. 26.6) | 25.1 (20.2. 27.2) | 24 (20.3. 27.3) | 25 (20.5. 27.8) | 26.3 (22.7. 28.9) | 180 (22.5. 300) | 480 (319. 1127) |
| P-value | 0.132 | 0.141 | 0.364 | 0.238 | 0.051 | 0.470 | 0.076 | 0.299 | 0.506 |
| *HDL-3-Apo-A1 (mg/dL)* | | | | | | | | |  |
| Soy protein | 28.2 (24.1. 30.3) | 26.8 (24.1. 30.4) | 26.2 (23. 29.2) | 27.1 (24.2. 29.7) | 26.6 (24.1. 29.3) | 28.3 (24.8. 31.3) | 29.2 (26. 32) | 240 (22.5. 300) | 597 (368. 1091) |
| Fatty fish | 25.8 (23.3. 29.4) | 24.9 (23.9. 27.9) | 25.3 (23.4. 28.5) | 24.6 (23.4. 30.2) | 24.8 (22.9. 29.5) | 26.3 (24.9. 29.5) | 27 (25.4. 32.5) | 240 (30. 300) | 493 (364. 1192) |
| Red meat | 26.5 (23.8. 29.7) | 26.5 (23.3. 28.5) | 26.4 (23.9. 28.6) | 26.4 (24. 29.4) | 26.2 (24.3. 30) | 27.2 (24.1. 29.1) | 28 (26.1. 31) | 150 (0. 300) | 618 (244. 912) |
| P-value | 0.603 | **0.046** | 0.486 | 0.406 | **0.040** | 0.095 | **0.032** | 0.561 | 0.323 |
| *HDL-4-Apo-A1 (mg/dL)* | | | | | | | | |  |
| Soy protein | 69 (64.6. 79.1) | 69.7 (64.3. 78.7) | 67 (59.6. 77.6) | 66.8 (58.9. 74.3) | 64.8 (59.3. 73.6) | 68.9 (60.2. 75.9) | 72.8 (66.8. 80.7) | 30 (0. 37.5) | 1100 (761. 1312) |
| Fatty fish | 69.1 (62.1. 79.2) | 67.9 (62.7. 79.1) | 68.1 (59. 79.2) | 66.4 (60. 77.3) | 65.6 (58.8. 73.3) | 62.6 (56.8. 70.9) | 71.1 (64.5. 84.9) | 30 (22.5. 120) | 1050 (643. 1456) |
| Red meat | 66.8 (61.5. 73.2) | 66.5 (63.2. 71.9) | 66.1 (61.6. 70.8) | 64.8 (58.3. 69.8) | 62.8 (59.7. 69.8) | 63.8 (57.3. 69.6) | 69 (65.8. 73.5) | 30 (0. 30) | 769 (488. 1320) |
| P-value | 0.339 | 0.603 | 0.799 | 0.506 | 0.988 | 0.120 | 0.323 | **0.009** | 0.323 |
| *HDL-1-Apo-A2 (mg/dL)* | | | | | | | | |  |
| Soy protein | 4.9 (4. 5.8) | 4.9 (3.8. 5.6) | 4.3 (3.3. 5.4) | 4.6 (3.6. 5.8) | 4.5 (3. 6) | 5.3 (3.6. 6) | 5.5 (4.6. 6.5) | 300 (22.5. 300) | 159 (110. 472) |
| Fatty fish | 4.5 (3.3. 5.4) | 3.9 (3.1. 5.1) | 4.2 (3.1. 5.4) | 4.6 (3.5. 5.5) | 4.6 (3.7. 5.6) | 5.1 (3.7. 6.4) | 5.2 (4.2. 6.4) | 300 (120. 300) | 231 (143. 558) |
| Red meat | 4.7 (3.6. 5.9) | 5.1 (3.4. 5.7) | 4.9 (3.8. 5.6) | 5.1 (3.7. 6.1) | 5.3 (4. 6.7) | 5 (3.7. 6.5) | 5.9 (4.5. 7) | 180 (97.5. 300) | 190 (108. 460) |
| P-value | 0.078 | 0.106 | 0.402 | 0.301 | **0.013** | 0.301 | 0.406 | 0.689 | 0.470 |
| *HDL-2-Apo-A2 (mg/dL)* | | | | | | | | |  |
| Soy protein | 4.3 (3.5. 4.7) | 4.1 (3.5. 4.6) | 3.7 (2.9. 4.4) | 3.9 (3.4. 4.6) | 4 (2.9. 4.7) | 4.3 (3.2. 5) | 4.7 (3.9. 5.3) | 180 (0. 300) | 141 (105. 395) |
| Fatty fish | 3.8 (3.2. 4.5) | 3.4 (3. 4) | 3.5 (2.9. 4.2) | 3.7 (3.1. 4.3) | 3.8 (3. 4.3) | 4.4 (3.4. 4.9) | 4.4 (3.9. 5.2) | 300 (105. 300) | 189 (154. 498) |
| Red meat | 3.8 (3.2. 4.8) | 3.7 (3.3. 4.3) | 3.7 (3.3. 4.2) | 3.8 (3.3. 4.5) | 4.1 (3.4. 4.8) | 4.1 (3.5. 4.8) | 4.6 (4. 5.3) | 105 (0. 300) | 142 (117. 364) |
| P-value | **0.037** | 0.110 | 0.603 | 0.549 | 0.220 | 0.761 | 0.633 | 0.363 | 0.258 |
| *HDL-3-Apo-A2 (mg/dL)* | | | | | | | | |  |
| Soy protein | 5.9 (4.8. 7.4) | 6.1 (4.6. 7.2) | 6 (4.2. 7) | 5.9 (4.9. 7.4) | 5.8 (4.6. 7.2) | 6.3 (5.1. 6.8) | 6.6 (5.7. 7.6) | 60 (22.5. 300) | 249 (125. 493) |
| Fatty fish | 5.5 (3.8. 6.4) | 5.4 (4.5. 5.8) | 5.4 (4.7. 6.3) | 5.3 (4.4. 6.2) | 5.1 (4.3. 6.2) | 5.5 (4.7. 6.4) | 6 (5.4. 7.1) | 90 (22.5. 300) | 230 (161. 503) |
| Red meat | 5.9 (4.4. 6.5) | 5.7 (4.7. 6.1) | 5.5 (4.8. 6.3) | 5.7 (4.2. 6.4) | 5.6 (4.6. 6.5) | 5.2 (4.7. 6.6) | 6.4 (5.6. 7) | 60 (22.5. 300) | 246 (112. 429) |
| P-value | 0.242 | 0.106 | 0.646 | **0.034** | 0.120 | 0.170 | **0.040** | 1.000 | 0.590 |
| *HDL-4-Apo-A2 (mg/dL)* | | | | | | | | |  |
| Soy protein | 16.2 (14.4. 19.2) | 16.2 (13.6. 20) | 15 (12.4. 19.3) | 16.3 (12.5. 17.7) | 15.3 (12.1. 18) | 16.7 (12.2. 19.1) | 17.7 (14.7. 20.6) | 30 (0. 37.5) | 386 (281. 508) |
| Fatty fish | 16.6 (12.8. 17.8) | 16.1 (13.4. 18.5) | 15.8 (12.8. 18.9) | 15.1 (12.9. 19.8) | 14.4 (12.7. 18) | 13.3 (11.1. 15.8) | 17.3 (14.3. 20.7) | 30 (22.5. 75) | 337 (206. 537) |
| Red meat | 14.7 (13. 18) | 14.6 (12.8. 18.3) | 14.5 (12.3. 17.1) | 13.7 (12.1. 17.5) | 13.8 (12.9. 16.3) | 13.6 (11.1. 16.4) | 15.3 (14.5. 18.6) | 30 (0. 30) | 323 (166. 581) |
| P-value | 0.132 | 0.646 | 0.859 | 0.348 | 0.791 | **< 0.001** | 0.177 | 0.192 | 0.177 |
| All hypothesis teste are performed with Friedman Test. using the exact P-value. 1 Each individuals highest concentration regardless of time. 2 Time when the highest concentration occurred for each individual. 3. Incremental over the lowest value. | | | | | | | | | |

**Supplemental Table 4**. Postprandial IL-6 after 5h and AUC_min_ of lipid fractions after each isocaloric meal with different protein

|  | **Red meat meal effect** | **Fatty fish meal effect** | **Soy protein meal effect** |  |
| --- | --- | --- | --- | --- |
|  | Mean (95% CI) | Mean (95% CI) | Mean (95% CI) | P-value^1^ |
| Il-6^2^ (log10 (ng/L)) | 0.866 (0.653, 1.08) | 0.89 (0.688, 1.09) | 0.943 (0.74, 1.15) | 0.857 |
| Triglycerides^3^ (mg/dL) | 8 150 (6 300, 9 990) | 8 750 (6 980, 10 500) | 8 140 (6 350, 9 930) | 0.804 |
| VLDL-4 Cholesterol^3^ (Log10(mg/dL)) | 2.22 (2.13, 2.32) | 2.48 (2.39, 2.57) | 2.35 (2.26, 2.44) | < 0.001 |
| VLDL-4 Free Cholesterol^3^ (Log10(mg/dL)) | 1.96 (1.85, 2.08) | 2.34 (2.23, 2.46) | 2.09 (1.98, 2.20) | < 0.001 |
| VLDL-4 Phospholipids^3^ (Log10(mg/dL)) | 2.06 (1.96, 2.16) | 2.46 (2.36, 2.55) | 2.22 (2.12, 2.31) | < 0.001 |
| VLDL-4 Triglycerides^3^ (√(mg/dL)) | 15.5 (12.8, 18.2) | 23.4 (20.8, 26) | 18.6 (16, 21.2) | < 0.001 |
| VLDL Particle numbers^3^ (nmol/L) | 6 110 (4 300, 7 910) | 9 130 (7 400, 10 900) | 6 900 (5 150, 8 640) | 0.017 |
| VLDL Cholesterol^3^ (Log10(mg/dL)) | 2.96 (2.87, 3.05) | 3.18 (3.09, 3.26) | 2.99 (2.9, 3.08) | < 0.001 |
| VLDL Free Cholesterol^3^ (Log10(mg/dL)) | 2.60 (2.52, 2.69) | 2.75 (2.67, 2.83) | 2.61 (2.53, 2.69) | 0.004 |
| VLDL Phospholipids^3^ (Ln10(mg/dL)) | 6.70 (6.48, 6.92) | 7.23 (7.02, 7.44) | 6.78 (6.57, 6.99) | < 0.001 |
| VLDL Triglycerides^3^ (Log10(mg/dL)) | 3.73 (3.63, 3.82) | 3.81 (3.72, 3.9) | 3.75 (3.66, 3.84) | 0.383 |

^1^ P-value of comparisons among the estimated marginal means.

^2^ Analyzed by a linear mixed model with treatment and baseline value, and diet quality index as fixed effects and subject as random intercepts.

^3^ Analyzed by a linear mixed model with treatment and baseline value, and diet quality index, age, BMI, physical activity index and DAS28 as fixed effects and subject as random intercepts.

**Supplemental Table 5**. Postprandial IL-6 after 5h and AUC_min_ of lipid fractions between isocaloric meals with different protein, n = 24.

|  | **Red meat vs fatty fish** | | **Red meat vs soy protein** | | **Soy protein vs fatty fish** | |
| --- | --- | --- | --- | --- | --- | --- |
|  | Mean (95% CI) | P-value | Mean (95% CI) | P-value | Mean (95% CI) | P-value |
| Il-6^1^ (log10 (ng/L)) | 1.15 (-0.311, 0.262) | 0.865 | -0.0771 (-0.364, 0.21) | 0.592 | 0.0528 (-0.226, 0.331) | 0.704 |
| Triglycerides^2^ (mg/dL) | -603 (-2 770, 1 560) | 0.578 | 6.71 (-2 190, 2 210) | 0.995 | -610 (-2 740, 1 520) | 0.567 |
| VLDL-4 Cholesterol^2^ (Log10(mg/dL)) | -0.26 (-0.381, -0.14) | < 0.001 | -0.131 (-0.253, -0.00952) | 0.035 | -0.129 (-0.249, -0.00985) | 0.035 |
| VLDL-4 Free Cholesterol^2^ (Log10(mg/dL)) | -0.381 (-0.507, -0.254) | < 0.001 | -0.13 (-0.253, -0.00572) | 0.041 | -0.251 (-0.376, -0.126) | < 0.001 |
| VLDL-4 Phospholipids^2^ (Log10(mg/dL)) | -0.397 (-0.52, -0.273) | < 0.001 | -0.157 (-0.281, -0.032) | 0.015 | -0.24 (-0.363, -0.117) | < 0.001 |
| VLDL-4 Triglycerides^2^ (√(mg/dL)) | -7.87 (-10.6, -5.14) | < 0.001 | -3.09 (-5.88, -0.297) | 0.031 | -4.79 (-7.51, -2.06) | 0.001 |
| VLDL Particle numbers (nmol/L) | -3 020 (-5 160, -888) | 0.007 | -792 (-2 960, 1 380) | 0.467 | -2 230 (-4 330, -128) | 0.038 |
| VLDL Cholesterol^2^ (Log10(mg/dL)) | -0.218 (-0.32, -0.116) | < 0.001 | -0.033 (-0.136, 0.07) | 0.522 | -0.185 (-0.286, -0.0844) | 0.001 |
| VLDL Free Cholesterol^2^ (Log10(mg/dL)) | -0.147 (-0.244, -0.0505) | 0.004 | -0.00342 (-0.101, 0.0941) | 0.944 | -0.144 (-0.239, -0.0493) | 0.004 |
| VLDL Phospholipids^2^ (Ln10(mg/dL)) | -0.529 (-0.771, -0.287) | < 0.001 | -0.0804 (-0.325, 0.165) | 0.512 | -0.449 (-0.685, -0.212) | < 0.001 |
| VLDL Triglycerides^2^ (Log10(mg/dL)) | -0.08 (-0.202, 0.0424) | 0.195 | -0.0182 (-0.142, 0.106) | 0.769 | -0.0618 (-0.181, 0.0578) | 0.303 |

^1^Analyzed by a linear mixed model with treatment and baseline value, and diet quality index as fixed effects and subject as random intercepts.

^2^Analyzed by a linear mixed model with treatment and baseline value, and diet quality index, age, BMI, physical activity index and DAS28 as fixed effects and subject as random intercepts.
